# Validated Reactive Force Field Quantifies MXene Interfacial Properties, Mechanics, and Thermal Transport

**DOI:** 10.1021/acsnano.5c20046

**Published:** 2026-07-22

**Authors:** Isaac W. Armstrong, Joseph M. Slocik, Leo Beck, Kennedy Brown, Christopher Muratore, Dhriti Nepal, Cheng Zhu, Vikas Varshney, Hendrik Heinz

**Affiliations:** † Materials Science and Engineering Program, University of Colorado, Boulder, Boulder, Colorado 80309, United States; ‡ Department of Chemical and Biological Engineering, University of Colorado, Boulder, Boulder, Colorado 80309, United States; § Materials and Manufacturing Directorate, 97042 Air Force Research Laboratory, , Wright-Patterson Air Force Base, Ohio 45433, United States; ∥ Department of Chemical and Materials Engineering, 2824 University of Dayton, Wright-Patterson Air Force Base, Ohio 45469, United States

**Keywords:** MXene, force field, surface chemistry, mechanical properties, solid−liquid interfaces, thermal conductivity, molecular dynamics

## Abstract

MXenes combine rich surface chemistry, mechanical strength, and high conductivity for a multitude of emerging applications. Predictive modeling supports accelerated materials designs and has been limited by the absence of validated and transferable force fields. Here, we introduce an interpretable, reactive INTERFACE force field (IFF and IFF-R) for Ti_3_C_2_T_*x*_ MXenes that is trained based on chemical knowledge and achieves quantitative agreement with experiments across lattice parameters (<0.5%), density (<0.2%), liquid contact angles, Raman spectra, and the in-plane elastic modulus (∼320 GPa). The models cover surface terminations from hydroxyl (−OH) to fluorine (−F) groups and are extensible to other chemistries. We introduce pH-resolved surface chemistry and identify dopamine adsorption mechanisms at MXene–aqueous interfaces supported by QCM-D and UV–Vis experiments. The data reveal coplanar and perpendicular binding modes and concentration-dependent multilayer assembly. We predict previously inaccessible properties, including termination-dependent cleavage energies, interlayer shear moduli and dynamic shear failure, nanoindentation and brittle fracture, anisotropic in-plane and out-of-plane thermal conductivities, including the role of defects. Agreement with available experimental data is consistently close and exceeds DFT accuracy across the benchmark properties examined. The IFF/IFF-R model is compatible with CHARMM, AMBER, OPLS, and CVFF force fields for simulations of MXenes with diverse surface terminations, electrolyte interfaces, biointerfaces, and polymer composites without additional parameters. Parameter sets, 3D models, and analysis scripts are provided for community use. The validated, reactive, and transferable IFF framework facilitates predictive design of MXene-based films, membranes, sensing interfaces, and composites.

## Graphical abstract

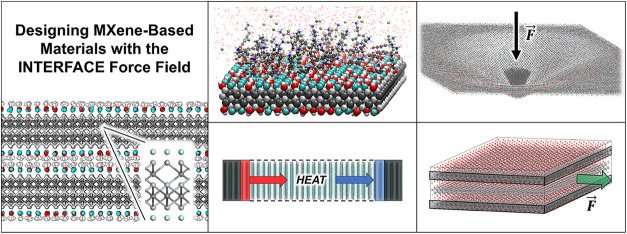

## 1 Introduction

Two-dimensional (2D) nanomaterials such as MXenes, graphene, hexagonal boron nitride (h-BN), and transition metal dichalcogenides (TMDs) have shown great promise toward a wide range of applications.^1–7^ MXenes are sheet-like early transition metal (M) carbides or nitrides (X = C, N) with the general composition of M_*n*+1_X*_n_* T_*x*_.^1^ They consist of covalently connected atomic layers, wherein the outer metal atomic layer contains surface groups (T) such as OH, F, or Cl (Figure 1).^8^ These 2D layers have a thickness of 8 to 20 Å and stack upon each other via Coulomb and van-der-Waals (vdW) interactions to form 3D lattices similar to clay minerals.

**Figure 1:**
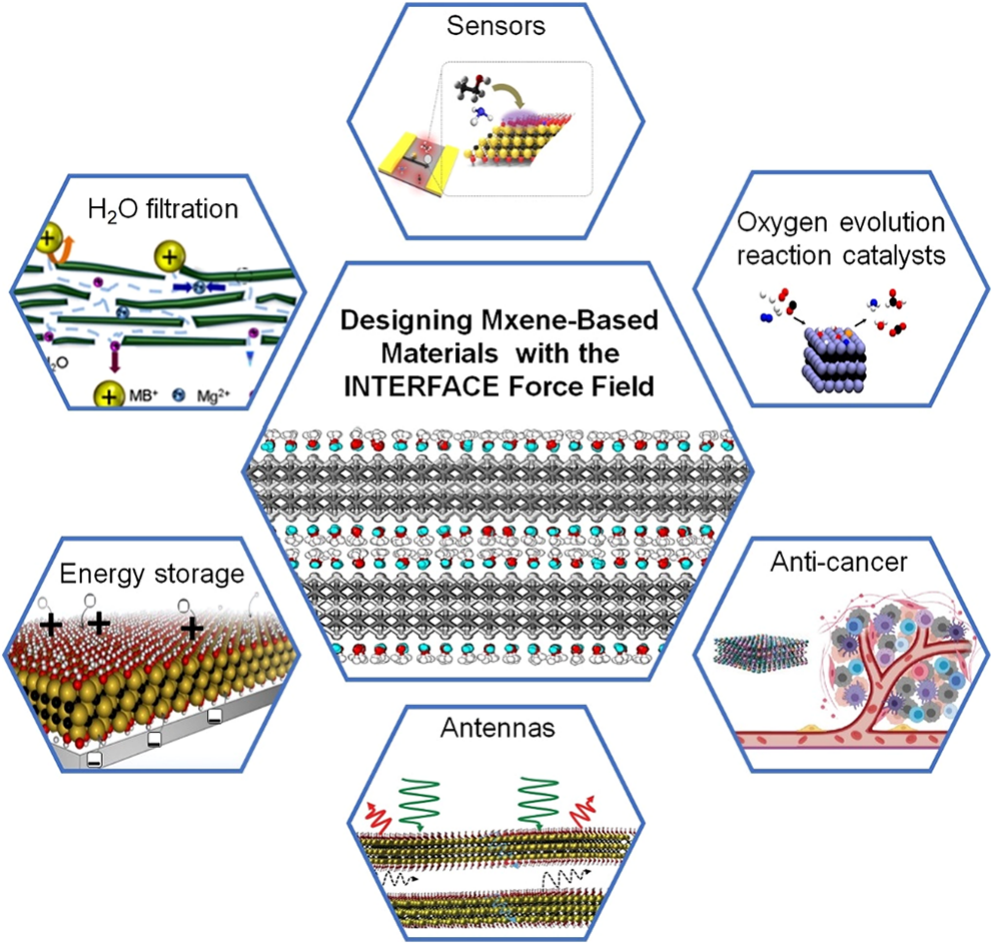
Opportunities for molecular dynamics simulations with the reactive INTERFACE force field (IFF-R) to codevelop applications of MXenes (Ti_3_C_2_T_*x*_) in multiple areas. Simulations with the chemically realistic models complement laboratory studies and guide in property prediction for new compositions, surface terminations, defects, electrolyte environments, and macromolecular assemblies. Several images were reused with permission. Catalysts: Reproduced with permission from ref 5 Copyright 2020 American Chemical Society. Anticancer: Reproduced with permission from ref 2 Copyright 2021 Taylor and Francis. Antennas: Reproduced with permission from ref 6 Copyright 2016 American Chemical Society. Filtration: Reproduced with permission from ref 4 Copyright 2015 American Chemical Society. Gas sensor: Reproduced with permission from ref 7 Copyright 2018 American Chemical Society. Energy storage and center image: self-prepared.

The large elemental space of transition metals (M) in combination with partially ionic and covalent bonding of surface termination groups (T) enables tailored properties. MXenes are electrical conductors or semiconductors and mechanically rigid like other carbides and nitrides, offering exceptional mechanical as well as electronic, optical, thermal, and organic assembly properties. Therefore, the combination of high 2D strength with desired electronic, biological, or catalytic functionality enables multifunctional applications (Figure 1).^9^ In comparison to graphene, MXenes remain expensive to produce and have stability limitations similar to TMDs. However, easy surface modification, high specific surface area and high electrical conductivity provide unique opportunities for the exploration of exciting hybrid materials.^1,10–12^

MXenes and MXene-based composite materials have been extensively characterized via X-ray diffraction (XRD), microscopy, spectroscopy, I/V curves, and binding properties in drug delivery.^2–7,9,13^ A challenge lies in understanding the role of atomic composition and surface functionalization for the assembly into macroscopic materials with tailored properties. Simulations can be helpful to increase the resolution relative to experiments, monitor dynamic processes, predict binding behavior, mechanical, and thermal properties from the atomic scale to the microscale, and quantify the impact of defects. Most simulation-based studies to-date have focused on density functional theory (DFT) methods due to easy availability.^14–17^ We appreciate the capabilities of DFT methods and higher-level quantum mechanical methods such as CCSD­(T) that come close to a ground truth. The studies have led to valuable insight into the electronic structure, vibration, and some thermal properties, however, they do not scale well to system sizes beyond 5 nm and extensive dynamics. Such scale is necessary to understand contact angles, electrolyte interfaces, molecular and polymer assembly, as well as shear stability, phonons, and defects. At the DFT level, we observe a broad distribution of computed properties relative to experiments, including lattice parameters, surface and elastic properties (Table 1).^18–20^ The interpretability of DFT results can be limited by assumptions about electron–electron correlations and differences to experimental data reach up to 100% depending on the density functional used.^21–23^ Furthermore, several prior studies employed ReaxFF,^24,25^ EAM,^25,26^ and COMB3 ^25,27^ potentials. These studies achieve mixed results and are difficult to scale for the simulation of larger systems such as electrolyte interfaces, biopolymer, and composite materials (Table 1). Specifically, the Universal Force Field (UFF)^28–30^ is known to yield unreliable or nonphysical results (Section S1 in the Supporting Information).^23^

**Table 1:** Key Properties of Ti_3_C_2_(OH, F)_2_ MXenes According to Experiments and Simulations with IFF-R, DFT, and Other Force Fields^f^

Property	Expt.	MD–IFF/IFFR	DFT	MD–ReaxFF	MD–EAM/PEF	MD–COMB3
Lattice parameter *a* (Å)	6.10 ^34^	6.06	6.16,^35^ 6.20,^36^ 6.22 ^37^	5.54, 6.32 ^25^	5.96, 6.06 ^25^	6.74,7.34 ^25^
*c* (Å)	19.86 ^34^	19.95	19.2 ^35^	20.14 ^24^	-	-
Density (g/cm^3^)	4.230 ^34^	4.244	4.29 ^35^	-	-	-
Bond length Ti_int_-C (Å)	2.123 ^34^	2.115	2.078,^38^ 2.088,^39^ 2.145 ^40^	-	-	-
Ti_ext_-C (Å)	2.143 ^34^	2.137	2.080,^38^ 2.195,^39^ 2.246 ^40^	-	-	-
Cleavage energy (0001) (mJ m^–2^)^a^	70–150^b^	118.5 ± 8	1500 ^41^	-	-	-
Water contact angle (°)^a^	48.5 ± 3 ^42^	48 ± 3	-	-	-	-
Elastic modulus *Y*_in‑plane_ (GPa)	330 ± 30 ^43^	319 ± 2.6	256,^15^ 316,^16^ 485 ^44^	386 ± 31 ^24^	502 ^26^	133 ^27^
Elastic Modulus *Y*_out‑of‑plane_ (GPa)^a^		36.7 ± 3.8	-	-	-	-
Shear Modulus, *G* (MPa)^a^	Unknown	18 ± 1^c^	-	-	-	-
Shear Strength, Τ (MPa)^a^	Unknown	153 ± 1^c^	-	-	-	-
Nanoindentation (Y/N)	Y	Y	N	Y (10x dev)^24^	Y	N
Thermal cond., *K*_in‑plane_ (Wm^1–^ K^–1^)	0.85–3 ^45^	2–7^d^	96.4,^46^ 108 ^47^	-	-	-
Thermal Cond., *K*_out‑of‑plane_ (Wm^1–^ K^–1^)	0.38 ^45^	0.56 ± 0.05	-	-	-	-
Compatibility with solvents (Y/N)	Y	Y	Limited	Limited	N	Limited^e^
pH-specific surface chemistry	Y	Y	N	N	N	N
Compatibility with (bio)polymers (Y/N)	Y	Y	N	N	N	Limited^e^
Number of parameters	NA	Low	High	High	High	High
Interpretability	NA	High	Low	Low	Medium	Medium
Relative computational cost for ∼10^5^ atoms	NA	1	∼10^12^ (DFT-MLFFs: ∼10^5^)	10^1^–10^2^	2 to 10	10^2^

a Mixed 50% OH and 50% F surface termination.

b Experimental data are not known. Expectation values are similar or higher than the surface tension of water (OH termination, 72 mJ m^–2^) due to hydrogen bonding and additional Ti polarity. Accordingly, 70–150 mJ m^–2^ is the expected range.

c For 100% OH termination.

d Low values were obtained in the presence of common defects.

e Requires additional parameter development.

f The agreement of IFF-R with experiment is ±0.3% for key structural properties, ±10% for surface properties, and ±10% for mechanical properties. Uncertainties with other methods including DFT are notably higher, up to 100%. DFT studies employed common density functionals (e.g., PBE, PBE-D2, PBE-D3, PBE + U).

In this work, we introduce the first validated and transferable force field for MXenes (Table 1), the reactive INTERFACE force field (IFF-R),^31,32^ and apply the model to predict a broad range of previously inaccessible properties. IFF and IFF-R combine comprehensive chemistry concepts in the parametrization with experimental validation from the atomic to the macroscopic scale,^31,32^ yielding up to an order-of-magnitude improvements in agreement with experiment for selected benchmark properties compared with earlier force fields and DFT/ML approaches (Table 1)^22,23^ This single transferable parameter set is intrinsically compatible with major biomolecular force fields.^33^ At low computational cost, parameter training and validation of lattice parameters, contact angles, and in-plane Young’s modulus enables predictions of other, to-date inaccessible properties. These properties include, and are not limited to, cleavage energies, contact angles with different solvents, adsorption mechanisms of biomolecules in aqueous solution, in-plane and out-of-plane elastic moduli, shear moduli and failure mechanisms, strain at break and failure mechanisms in nanoindentation, as well as directional thermal transport as a function of surface termination (-F vs −OH), in excellent agreement with experiments (Table 1). The results facilitate explanations of the observed trends and underlying mechanisms at the molecular scale. First, we introduce this model and rationale, then discuss surface properties, biomolecular binding, nanomechanical as well as thermal transport properties from the atomic to the micrometer-scale. The models can be extended to similar MXenes, unlimited materials combinations, and first examples of defective MXenes highlight their impact on thermal properties.

## 2 Results and Discussion

### 2.1 Derivation and Validation of Force Field Parameters

The derivation of force field parameters for MXenes (Figure 2a–e) followed the known IFF chemistry principles and workflow (Figure 2f),^31^ which have been extensively documented in earlier work on metals, oxides, liquids, gases, and other 2D materials (graphene, MoS_2_).^48,49^ The IFF energy expression is a Class I potential identical to CVFF,^50^ DREIDING,^51,52^ CHARMM,^53^ AMBER,^54^ and OPLS-AA,^55^ and includes full Class II compatibility (PCFF,^56^ CFF,^57^ and COMPASS^58,59^ ) (Figure S1 and Section S2 in the Supporting Information). Energy terms include quadratic bond stretching and angle bending, Coulomb interactions, as well as Lennard-Jones (LJ) parameters with 12–6 and 9–6 options (Table 2). Replacement of harmonic bonds by shifted Morse bonds in IFF-R with equivalent bond stiffness and bond dissociation energies adds reactivity to IFF and force fields with a compatible energy expression.^32^

**Figure 2:**
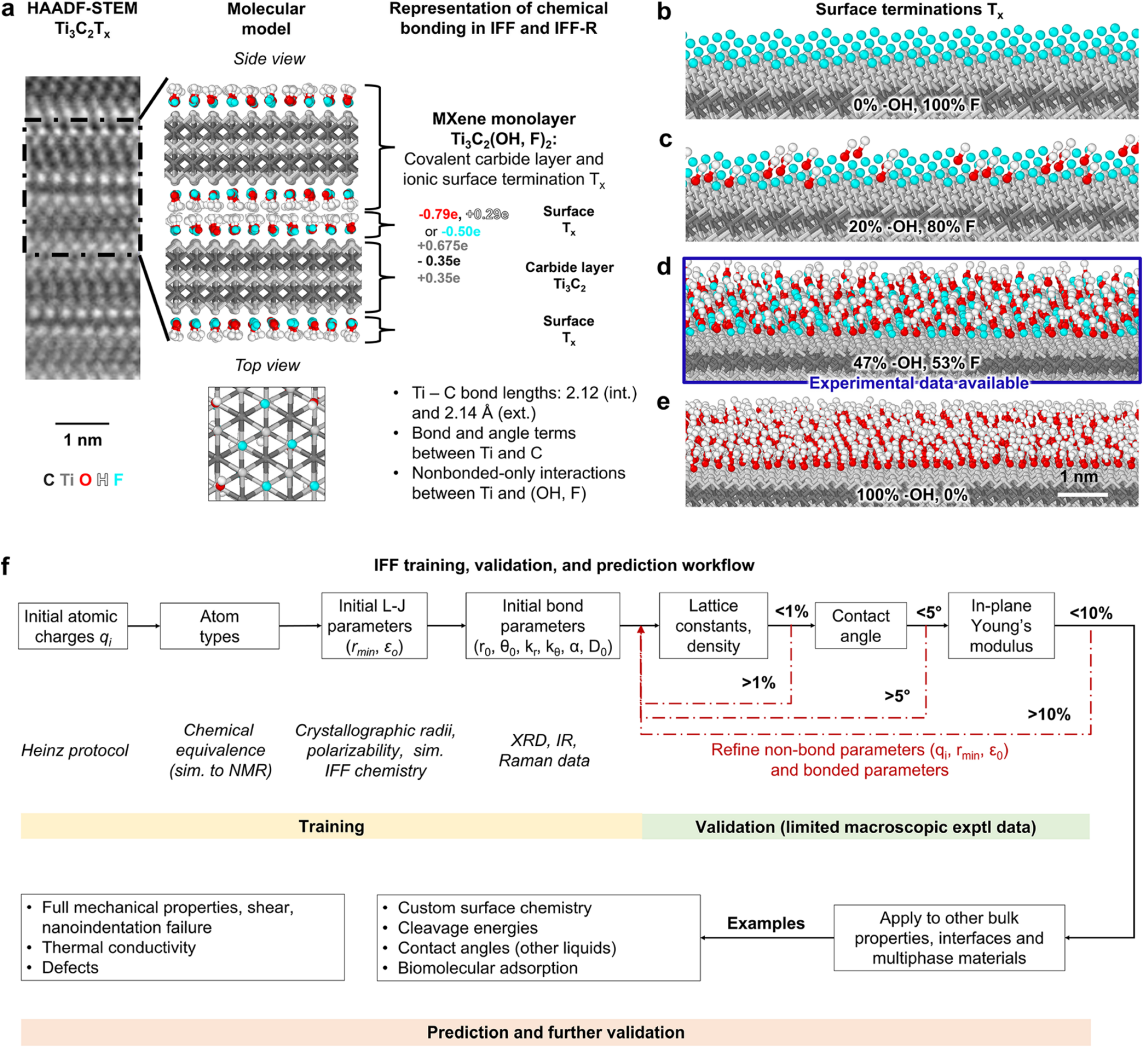
Structure of Ti_3_C_2_(OH, F)_2_ MXene and representation in IFF/IFF-R molecular models. (a) A scanning transmission electron microscope (STEM) image^60^ and the corresponding molecular model based on the X-ray structure from Shi et al. 2014.^34^ The assignment of force field parameters is based on the analysis of atomic charges *q*.^61^ The *q* values indicate a largely covalent inner Ti carbide layer and significantly ionic 4-electron-4-center bonds to surface groups, e.g., Ti_3_OH/Ti­(OH)_3_ units (top view). Each stoichiometric layer contains a five-atom thick Ti–C–Ti–C-Ti sequence, and the outer Ti layers carry Ti­(OH, F) groups.^62^ One type of reactive Ti–C bond describes the internal and external Ti–C bonds in the model, and several angle terms the near-octahedral geometry. Surface groups interact with Ti via nonbonded interactions due to significant polarity, and the nonbonded interactions enable easy exchange of surface modifications. (b–e) Illustration of MXene layer surface functionalities from fully fluorinated (-F) (b) to fully hydroxylated (−OH) (e). Typical etching protocols in experiments^62^ yield ∼47% OH and ∼53% F as shown in (d).^42^ (f) The workflow of IFF/IFF-R model training, validation, and prediction adapted for MXenes (see refs 23,31,32 for details).

**Table 2:** IFF and IFF-R Force Field Parameters for Ti_3_C_2_T_*x*_ ^d^

		Lennard-Jones Potential				
		*r*_min_ (Å)			ε (kcal mol^–1^)	
Nonbond parameters	Atomic charges *q* (e)	12–6: CVFF, OPLS-AA	12–6: AMBER, CHARMM	9–6: PCFF, CFF, COMPASS	12–6	9–6
Ti_int_	0.35	2.25	2.16	2.30	0.2	0.16
Ti_ext_	0.675	2.15	2.06	2.20	0.3	0.24
C	–0.35	3.80	3.80	3.90	0.07	0.06
F	–0.5	3.26	3.26	3.40	0.17	0.14
O	–0.788	3.45	3.45	3.60	0.19	0.16
H	0.288	1.085^a^	1.085^a^	1.098	0.015^a^	0.013
**Bonds**	***r***_***0***_ **(Å)**	***K***_***r***_ **(kcal mol** ^**–1**^ **Å** ^**–2**^ **)**				
Ti–C (all)	2.157	285				
**Reactive bonds** ^32^	***r***_***0***_ **(Å)**	***α*** **(Å** ^**–1**^ **)**			***D*** **(kJ mol** ^**–**^ **)**	
Ti–C (all)	2.157	2.0			60	
**Bond angles**	**θ**_**0**_ **(°)**	***K***_***θ***_ **(kcal mol** ^**–1**^ **rad** ^**–2**^ **)**				
Ti_ext_-C_*x*_-Ti_int_ ^b^	90	0				
Ti_ext_-C_*x*_-Ti_ext_	93.5	67				
Ti_int_-C_*x*_-Ti_int_	91.6	67				
C_*x*_–Ti-C_*y*_ ^c^	90	40				
C_*x*_-Ti_int_-C_*x*_ ^c^	180	67				

a LJ parameters for H can be chosen as zero (e.g., *r*_min_ = 0.001 Å and ε = 0.001 kcal/mol) with marginal changes in computed properties.

b A total of 4 carbon atom types are represent all unique bond angles (C_*x*_, *x* = 1, 2, 3, 4).

c For carbon types, *x*, *y* = 1, 2, 3, 4, *x* ≠ *y*. Ti refers to any titanium atom. See full list of bonds and bond angles in Table S1 in the Supporting Information.

d The complete topology of bonds and bond angles is shown in Table S1 and Figure S2 in the Supporting Information.

For training (Figure 2f), the MXene structure (Figure 2a) from X-ray diffraction^34^ was analyzed to identify bonding types and atomic charges. Atomic charges were derived using the Extended Born model introduced by Heinz et al.^61,63^ This approach takes into account atomic charges from experimental X-ray deformation electron densities, dipole moments, atomization energies, ionization energies, bond lengths, coordination numbers, and chemically similar compounds already validated in IFF (e.g., titania, CO_2_, organic compounds^31^). Accordingly, the inner 6-fold coordinated TiC carbide layer is predominantly covalent with net atomic charges of ±0.35*e*, and the outside Ti layers in contact with OH or F groups feature 3-fold carbide and 3-fold oxide/fluoride coordination that leads to higher polarity and Ti atomic charges of +0.675e. OH^–^ and F^–^ groups are sensitive to the interlayer and solvent environment and carry atomic charges of −0.788e (O), +0.288*e* (H), and −0.5*e* (F), equal to approximately half ionic and half covalent bonds (Figure 2b–e). The final values of the atomic charges were obtained in conjunction with Lennard-Jones parameters upon validation of contact angles with H_2_O and CH_2_I_2_ (Figure 2f). The H charges in OH are in the typical range for AlOH groups (point of zero charge 8 to 9 with +0.25*e*),^23,64^ TiOH groups (pzc ∼6.0 and +0.30*e*)^64^ and more acidic SiOH groups (pzc ∼3.0 and *H* charge of +0.40*e*) in previously validated IFF models.^65^

The bonded parameters follow the X-ray crystal structure^34^ and Ti–C bond dissociation energies.^66^ Two types of Ti–C bonds, internal and external (Figure 2a), are represented by one Ti–C bond parameter *r*_0_ = 2.157 Å that reproduces the two environmentally dependent bond lengths of 2.11 Å and 2.13 Å in X-ray data in molecular dynamics (MD) simulations (Tables 1 and 3). DFT does not recognize this difference of 0.02 Å^38^ or inflates itto 0.10 Å.^39,40^ Bond angles θ_0_ follow the octahedral coordination pattern (Table 2). The harmonic bond stretching constants *K*_*r*_ and angle bending constants *K*_θ_ were tuned to reproduce experimentally observed IR and Raman spectra (Figures S2, S3, and Section S3 in the Supporting Information). Some values of *K*_θ_ were set to zero to avoid overspecification of angles and excessive angle stiffness (Table 2).^48^ The parameters *K*_*r*_ and *K*_θ_, along with nonbonded parameters, reproduce the in-plane modulus of MXenes within the experimental uncertainty of ±10%. For reactive simulations with IFF-R, we employ the same bond length parameter *r*_0_ and a value of α = 2.0 that matches the same bond vibration properties (Table 2).^32^ The Ti–O bond dissociation energy *D* is ∼60 kJ/mol^66,67^ according to experimental values for chemically similar bonds within ±10% uncertainty. The value is also consistent with the heat of formation of titanium carbide (TiC), −180 kJ/mol,^67^ equal to one-third as a per-bond contribution in the octahedral geometry.

**Table 3:** Comparison of Lattice Parameters, Ti–C Bond Lengths, and Density of Ti_3_C_2_OH_0.5_F_0.5_ MXene According to X-ray Diffraction and IFF Molecular Dynamics Simulation^b^

	Lattice Parameters					Ti–C_ext_ bond length	Ti–C_int_ bond length	Density
Method	a, b (Å)	c (Å)	α (°)	β (°)	γ (°)	(Å)	(Å)	(g/cm^3^)
XRD^34^	6.10	19.86	90.00	90.00	120.00	2.143	2.123	4.230
IFF MD	6.06	19.95	90.23	89.79	119.90	2.137	2.115	4.2442
Diff. to expt.	0.66%	0.45%	0.21%	0.23%	0.08%	0.27%	0.37%	0.33%
Diff. to expt. using DFT	0.96%^35^	3.32%^35^	NA^a^	NA^a^	NA^a^	2.42%^39^	1.65%^39^	1.46%^35^

a Not reported or artificially constrained.

b Errors with best-performing density functionals relative to experiments are clearly higher.

Initial Lennard-Jones (LJ) parameters were estimated from the IFF database by chemical analogy. The Ti equilibrium nonbond diameter *r*_min_ of 4.5 Å is similar to Co (4.5 Å),^68^ Al (4.6 Å)^69,70^ and Si (4.15–4.5 Å)^65,71^ in similar chemical environments. The well depth *ε* of 0.20 kcal/mol is slightly larger than for the comparison chemistries (oxides/hydroxides) due to lower Ti atomic charge and higher polarizability, similar to V_2_O_3_.^72^ The LJ parameters for carbon are typical hydrocarbon and diamond parameters,^22,49^ and O and H are typical OH^–^ group parameters (e.g., phosphate, silicate, carbonate, titanate).^31,65^ LJ parameters for F^–^ were obtained by analogy to N and O using Halgren’s rule.^31,73^ Accordingly, F^–^ is smaller than O in OH^–^ (3.26 vs 3.45 Å) and expected to have slightly larger polarizability (ε) at equal charge (smaller ε of 0.17 vs 0.19 kcal/mol due to lower negative charge) (Table 2). Parameters were initially derived and validated for IFF-CVFF, OPLS-AA, and subsequently extended and tested for the other energy expressions.

For validation (Figure 2f), the initial parameters were tested in MD simulations to reproduce a stable structure (lattice parameters), energy differences (known water contact angle as a proxy for unknown surface energies), and energy derivatives (Young’s modulus).^31^ The initial parameters worked well with deviations of ∼3%, ∼10°, and ∼20%. Optimization of this training set to reproduce these key experimental properties was carried out via iterations following a three-step approach: (1) Refinement of LJ parameters to match the lattice parameters, density, and atomic positions at equilibrium relative to X-ray data; (2) Refinement of atomic charges of surface groups to match the experimental contact angles; (3) Refinement of bond and angle constants to match experimental Raman vibrational frequencies and in-plane Young’s modulus. Grounding in detailed chemistry knowledge and experimental data from the atomic scale to the macroscale only allowed variations within small physically reasonable ranges of the parameters and no further empirical fit parameters (see Section S2 in the Supporting Information). In total, we carried out over 1000 test calculations, including some out-of-range (unphysical) parameters for verification, resulting in the listed parameters (Table 2) and an extensible set of surface models for various MXene surface chemistries (Figure 2b–e).

The ability to reproduce key macroscopic experimental data using these interpretable parameters provides strong validation. The IFF parameters for the Ti_3_C_2_T_*x*_ MXene correlate well with previously validated parameters for similar chemistries.

The following subsections illustrate predictions and further experimental validation (Figure 2f) of structural, energetic, biomolecular binding, mechanical, and thermal properties. The results surpass the accuracy of current simulation methods by 3 to 100 times (Table 1). The inclusion of solvents, electrolytes, organic molecules, and (bio)­polymers utilizes compatibility with existing force fields and requires no additional parameters.^31^ Paired with leading computational speed in all-atom simulations, this force field is a practical choice to access large systems for a diverse set of applications.

### 2.2 MXene Crystal Structure

The crystallographic structure from X-ray diffraction data by Shi et al.^34^ is reproduced with average deviations of 0.3% in lattice parameters and 0.3% in density (Table 3). The root-mean-square difference in all atomic positions in the supercell was 0.012 Å relative to experiment. Shi et al. assumed statistical surface termination by – F and – OH across surface sites and we included randomly distributed hydroxyl (−OH) and fluoride (-F) groups in a 47/53 split in our model (Figure 2d). DFT results, assuming the best-performing functionals, show 1% to 3% errors in lattice geometry and density, compared to <0.5% errors in IFF and IFF-R (Table 3). Similar X-ray structures, e.g., with bridging -O- terminations and other surface chemistries can also be covered by our model.^74^ The benefit of IFF and IFF-R models increases further when simulating electrolyte, organic, or inorganic interfaces where the accuracy is maintained while density functionals for transition metals face trade-offs for electrolytes and organic compounds in the same simulation.

### 2.3 Surface Energy and Solid–Liquid Interfacial Properties as a Function of Surface Terminal Group

The (0001) surface energy, or layer cleavage energy (Figure 3a), is a key property that determines how MXene surfaces interact with their environments, e.g., adsorption, electrolyte dynamics, and shear properties.^48,70,75,76^ The surface energy, in the absence of surface reconstruction upon cleavage, is also related to the static contact angle (θ) of a droplet of probe liquid.^77^ Computations of the cleavage energy with our model show major differences depending on surface chemistry, such as 131 ± 4 mJ/m^2^ for 100% OH termination, 119 ± 4 mJ/m^2^ for 50% OH/50% F termination, and 74 ± 5 mJ/m^2^ for 100% F termination (Figure 3b). Accordingly, OH terminated surfaces have significantly higher cohesion than F-terminated surfaces, and 50% OH content is already sufficient to closely approach the maximum surface energy. The number of MXene layers (1, 2, 3, 4) plays no discernible role since the thickness of a single electroneutral layer of 1.0 nm is sufficient to cover most van-der-Waals and Coulomb interactions (Figure 3b).

**Figure 3:**
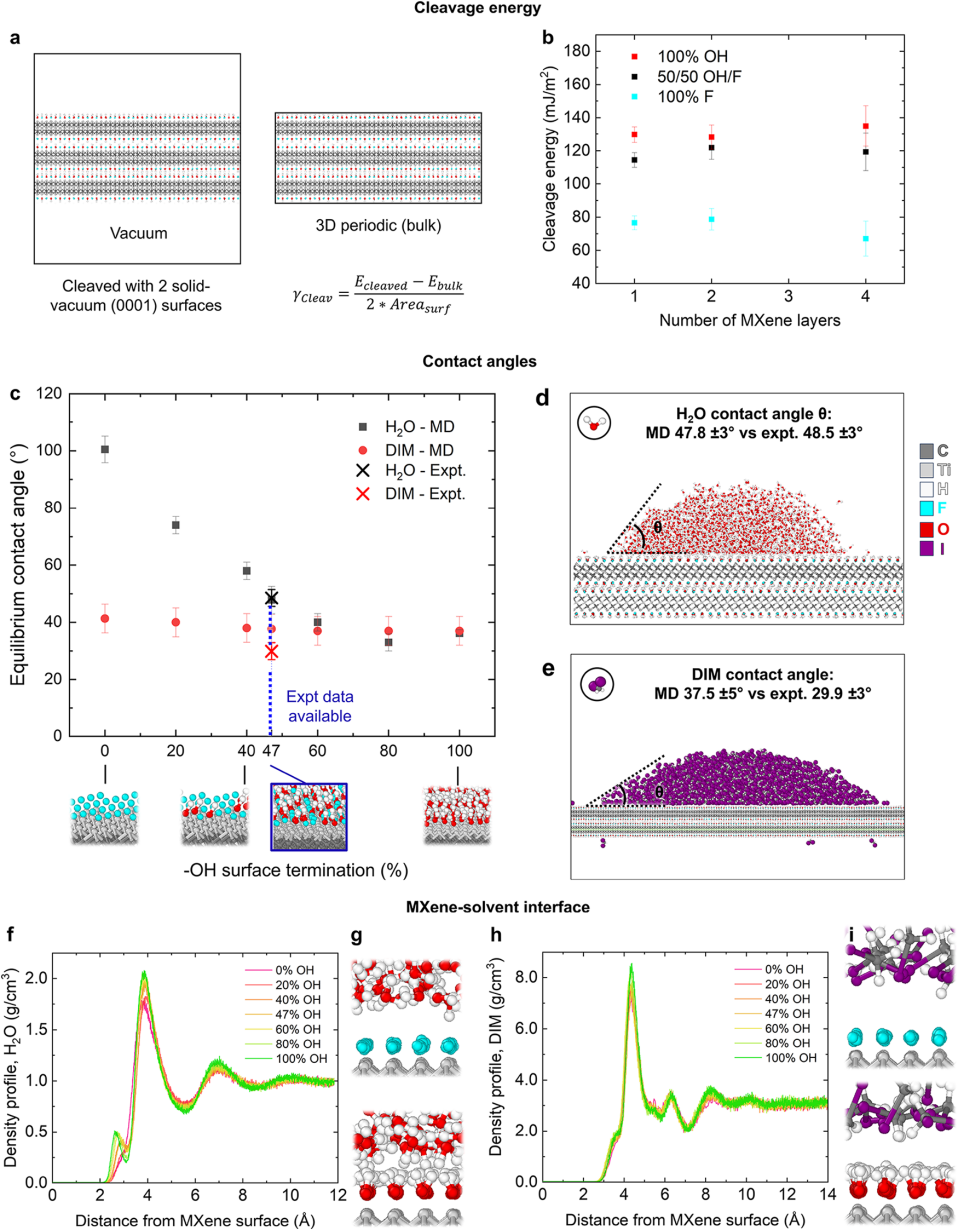
Surface and solid–liquid interfacial properties of Ti_3_C_2_(OH, F)_2_ from MD simulation and experimental data. (a) Calculation of the (0001) cleavage energy, i.e., the (0001) surface energy upon formation from the bulk. (b) Surface energy as a function of terminal group and layer thickness. (c) Equilibrium contact angles of water (H_2_O) and diiodomethane (DIM, CH_2_I_2_) on MXene surfaces as a function of surface functional groups from 100% -F, 0% −OH to 0% -F, 100% −OH. Simulation results agree with available experimental data for 47% −OH coverage.^42,79^ MD simulations predict that water contact angles to increase up to ∼100° for full -F termination and DIM contact angles are not sensitive to surface groups. (d, e) Equilibrium snapshots of water and DIM droplets on MXene with 47% OH coverage from MD simulations and available experimental data.^42,79^ (f) Equilibrium density profiles of H_2_O on MXene surfaces for different surface treatments. Increasing −OH% on the surface leads to closer contact of H_2_O via hydrogen bonds at 2.7 to 3.0 Å distance. (g) Visualization of the interfacial structure for full F and full OH coverage. Major differences in no hydrogen bonding vs strong hydrogen bonding are seen. (h) Equilibrium density profiles of DIM on MXene surfaces for different surface treatments are similar. (i) Visualization of the interfacial structure for full F and full OH coverage shows structural differences, but no hydrogen bonding in either case.

Contact angles θ have been measured in experiments, further characterize the surface properties and are suitable to validate the force field (Figure 3c–e). H_2_O and diiodomethane (DIM) are common probe liquids due to their highly polar and dispersive nature, respectively. Experimental measurements on MXene thins films with 47% hydroxyl surface coverage identified 48.5° for water and 30° diiodomethane (CH_2_I_2_)^42^ with typical uncertainties of ± 3°.^78^ Measurements by other groups have also reported water contact angles of 45–55° and DIM contact angles of 20–25° (see Section S4 and Figure S4 in the Supporting Information).^79^ MD simulations reproduce these benchmarks well, leading to 48 ± 3° for water (flexible SPC model) and 37.5 ± 3° for CH_2_I_2_ (IFF model^48^) (Figure 3c–e).

Across a broader range of surface chemistry, we predict a strong dependence of the water contact angle on the fraction of OH groups, with a maximum of 100° on fully fluorinated surfaces, a minimum of 33° for 80% OH-terminated surfaces, followed by a slight increase to 36° for 100% OH-terminated surfaces (Figure 3c). The diiodomethane contact angle was hardly affected by the surface termination and ranged from 42° for 100% F termination to 37° for 50% or higher OH termination (Figure 3c). Since no quantitative data has been available for different surface terminations to-date, these trends can be useful to design wetting properties of MXene surfaces. The observations are consistent with data for other fluorinated and hydrophilic surfaces such as functionalized polymers and carbon films that exhibit increased hydrophobicity at high F content.^80,81^ Under laboratory conditions, possible oxidation over time can modify MXene surface chemistry and contact angles (Figure S4 in the Supporting Information).^79^

The computed density profiles of water for different surface terminations show further details (Figure 3f). A stronger first peak at 2.7 to 3.0 Å distance and a taller second peak at ∼3.8 Å distance are seen for high OH content, related to directional hydrogen bonding and higher interfacial packing density (Figure 3g). The density profiles for DIM at the interface are less sensitive to the surface termination (Figure 3h), however, higher OH coverage also leads to increased density at ∼4.3 Å distance from the interface (Figure 3i). Furthermore, OH-containing MXene surfaces undergo pH-dependent protonation and deprotonation reactions in water, which will be discussed in the following.

### 2.4 pH-Sensitive Surface Chemistry and Mechanisms of Biomolecular Adsorption

Ti_3_C_2_T_*x*_ MXenes have shown promise in the detection of dopamine^82^ and formation of thin films^83^ for biosensors. Understanding of the binding mechanisms can be obtained by MD simulations and requires insight into MXene surface chemistry at specific pH values (Figure 4a,b). Superficial OH groups undergo protonation and deprotonation reactions as a function of pH with a point of zero charge (PZC) of 6 ± 1, similar to rutile and anatase phases of titania.^64,84^ F, Cl, and other nonprotic surface groups are less affected by changes in pH value. The pH value for full OH coverage of 6 ± 1 is an approximation by analogy to Ti-oxides^64,84^ since measurements for Ti_3_C_2_T_*x*_ MXenes have not yet been reported. The PZC relates to surface charge neutrality and the isoelectric point (IEP) to electrokinetic neutrality, whereby the IEP is typically the same as the PZC unless protic ions other than H_3_O^+^ and OH^–^ would be present in the electrolyte.^64^ According to data from surface titration experiments, up to ∼15% of Ti_3_(OH) groups form positively charged Ti_3_(OH_2_)^+^ Cl^–^ groups as the pH value decreases from 6 to 1, and up to 15% negatively charged Ti_3_O^–^ Na^+^ groups as the pH value increases from 6 to 11 (Figure 4a).^85–88^ Accordingly, the area density of ionic surface groups in MXenes surpasses +0.5 per nm^2^ or −0.5 per nm^2^ as pH values deviate from the PZC by several units.^85–88^ As described earlier in the IFF surface model database^31^ for silicate,^65,89,90^ phosphate,^91–93^ and aluminate surfaces,^23,69,94^ the quantitative representation of the charge state is critical to predict binding properties of solvents, solutes, and polymers. We include modified atomic charges for protonated OH_2_^+^ surface groups that accommodate the positive charge (see Section 2 in the Supporting Information and Supporting Files).^65^

**Figure 4:**
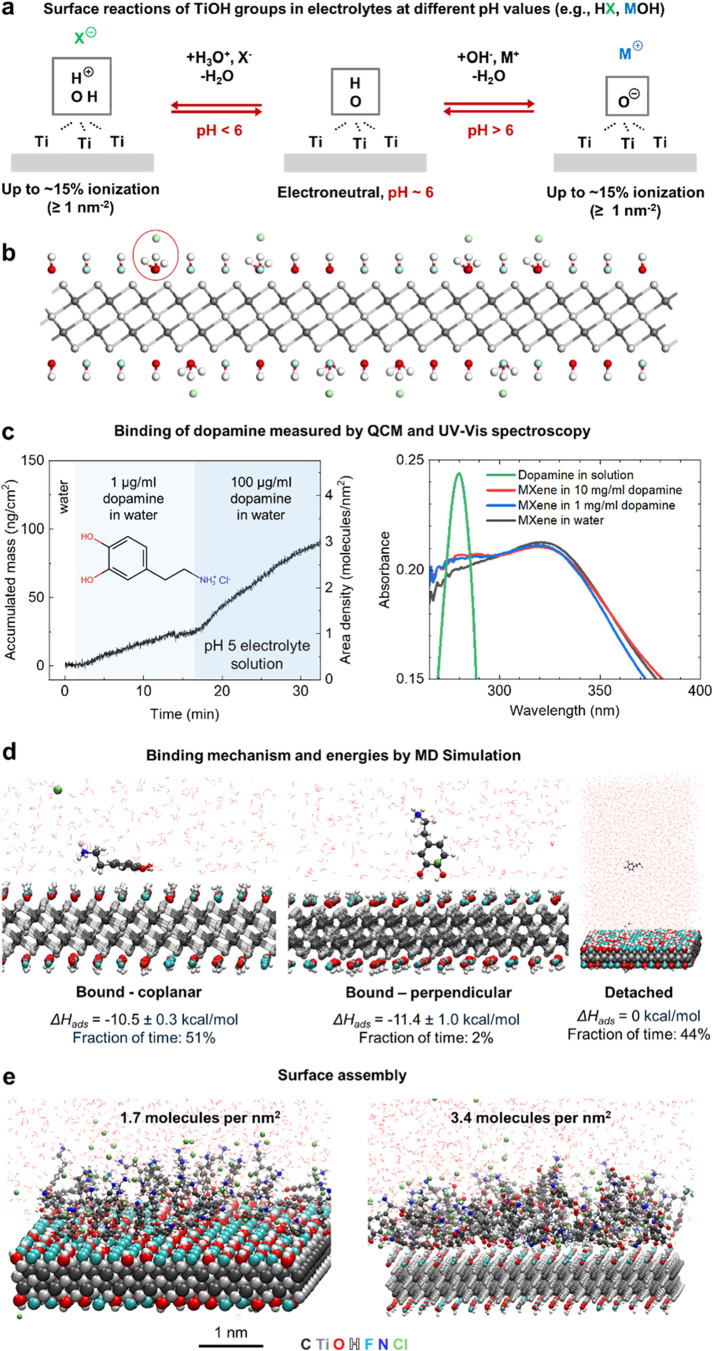
pH-resolved surface models for MXenes and dopamine binding mechanism. (a) Surface models for MXene with OH terminal groups as a function of pH value. At pH values lower than 6, we expect partial protonation and at pH values higher than 6, partial deprotonation. (b) Illustration of a 53% F, 47% OH terminated MXene surface when exposed to an electrolyte at pH 5, showing some Ti_3_(OH_2_)^+^ Cl^–^ surface groups (highlighted). (c) Measurements of adsorption of dopamine hydrochloride onto MXene by QCM-D and UV–vis spectroscopy. (d) Binding mechanisms and orientational preferences of a single dopamine hydrochloride ion pair onto the MXene surface. (e) The assembly of 40 and 80 dopamine molecules, respectively, occurred randomly and increased perpendicular orientations at higher coverage. The 3D periodic model size was 48.808 × 47.5524(∼80) Å^3^.

The adsorption of dopamine chloride on MXene films was studied experimentally in deionized water using a quartz-crystal microbalance (QCM-D) and UV spectroscopy (Figure 4c). Hereby, the QCM chip was coated with a solution-processed MXene film. The solution assumed a pH value of ∼5 due to partial deprotonation of the alkylammonium group of dopamine in water (p*K*_1_ = 8.4).^95^ At pH 5, we also deprotonated some of the OH groups on the (OH, F) terminated MXene surface in the corresponding 3D model (Figure 4b) for simulations of dopamine hydrochloride single molecule adsorption and self-assembly of films (Figure 4d,e and Table 4). Experiments with the QCM-D show an uptake of dopamine hydrochloride by the MXene to about 1 molecule per nm^2^ at 1 μg/mL concentration, and to about 3 molecules per nm^2^ at 100 μg/mL concentration (Figure 4c). In total, the QCM-D binding responses yielded a margin of error of 4.3% and 1.4% for 1 μg/mL and 100 μg/mL, respectively. This associated error is in agreement with previous QCM binding studies of small molecule amino acids and demonstrates the accuracy of detection for dopamine binding on solution-processed MXene coated QCM crystals.^96^

**Table 4:** Binding Enthalpy and Fraction of Time of Different Configurations of a Single Molecule of Dopamine Hydrochloride (<10% Surface Coverage) in Contact with the MXene Surface in MD Simulation^c^

Orientation^a^	Binding enthalpy (kcal/mol)	Fraction of time (%)	Binding free energy^b^ (kcal/mol)
Coplanar, bound (<6 Å)	–10.5 ± 0.3	50.8 ± 0.4	–0.5
Perpendicular, bound (<6 Å)	–11.4 ± 1.0	2.0 ± 0.1	+2
Nearby surface (two or few water layers away, 6–12 Å)	+5.7 ± 1.0	3.4 ± 0.1	+1.5
Detached (>12 Å away)	0.0 ± 0.3	43.9 ± 0.4	0

a For the definition of bound, nearby, and detached states, see Section S5 and Figure S5 in the Supporting Information.

b The binding free energy Δ*G* was calculated from the equilibrium constant *K* of time “bound” in a certain state versus the time detached in solution (Δ*G* = −*RT ln K*). The uncertainty of the average values is ±0.5 kcal/mol.

c The data are averages over 100 ns simulation time.

The laboratory data suggest an ordering process on the surface with relatively slow kinetics, indicative of favorable adsorption but not immediate, unconditional binding. To further verify dopamine binding, UV measurements showed a typical peak at 280 nm for dopamine hydrochloride in water (Figure 4c, green curve). The same peak emerged when redispersing MXene films in water that were previously exposed to dopamine (Figure 4c, red and blue curves). A clearly higher absorbance at 280 nm was then seen relative to pure MXene in water (Figure 4c, black curve), reaffirming the binding affinity of dopamine hydrochloride to MXene for two initial concentrations (1 and 10 mg/mL).

IFF is compatible with standard solvent parameters and biomolecular force fields such as CHARMM (see explanation in refs 31,33 ). MD simulations using IFF-CHARMM36 confirm the dopamine chloride binding affinity relative to water on the MXene surface with two preferred conformations (Figure 4d). Coplanar bound conformations were most common and had a binding enthalpy of −10.5 kcal/mol, followed by perpendicular conformations with slightly stronger binding enthalpy of −11.4 kcal/mol but lower fractions of contact time (entropically less preferred) (Table 4). In both instances, the OH groups in dopamine are in contact with the MXene surface and the ammonium group avoids direct contact, remaining at least one water layer away (Figure 4d). The aromatic ring was located at ∼3.2 Å distance from (**O**H, F) groups in the coplanar state. Chloride ions associated with dopamine below pH 8 for charge neutrality, were delocalized within 2 nm distance from the surface in the bound state and maintained a distance of at least one water layer. Another metastable, transient binding state few water layers (6–12 Å) away from the equilibrium binding distance was observed at +5.7 kcal/mol (Table 4). The energy penalty originates from a disruption of the H-bonded network of the intermediate water layer by the aromatic ring and alkyl chain. Dopamine mostly binds in between the first and second water layers at 3.2 and 5.7 Å distance (Figure 3f). This position minimizes interference with hydrogen bonds of water to the surface (Figure 3g). Free energies of binding Δ*G*_ads_, computed from the time fractions bound versus detached in simulations over 100 ns, are only between 0 and −1 kcal/mol (Table 4, *K*_ads_ = *t*_bound_/*t*_detached_, Δ*G*_ads_ = −*RT ln K*_ads_ = Δ*H*_ads_–*T*Δ*S*_ads_). Accordingly, the entropy of binding is negative (−*T*Δ*S*_ads_ ∼ +10 kcal/mol) and likely related to reduced mobility of the affected MXene surface groups, loss of conformational flexibility of bound dopamine, and small gains in entropy of water molecules that are displaced from the surface upon binding.^89^

Simulation of the assembly of multiple dopamine molecules indicates random side-by-side orientations (Figure 4e). Randomness at a coverage of 1.7 molecules per nm^2^ intensifies through the chloride ions, which maintain electroneutrality of the ammonium groups and shorten the average molecular distance as the packing density increases. At 3.4 dopamine hydrochloride molecules per nm^2^, bound molecules prefer a perpendicular orientation (Figure 4d and Table 4) that is entropically better supported in this environment. The results are consistent with the QCM-D measurements that indicate attraction and concentration-dependent random layer growth. Hereby, the laboratory time scale is minutes in comparison to microseconds in the simulations. Ordered assembly could not be achieved in the simulations: even when biasing initial orientations of dopamine, orientations randomized and a fraction of dopamine re-entered the equilibrium between detached states versus bound states. Alternatively, condensation reactions of Ti_3_OH groups on the MXene surface with Ph–OH groups in dopamine could lead to covalent Ti_3_O-Ph bonds (or Ti_3_O–C–C-OTi_3_ geminal bonds) and support the growth of ordered dopamine films. The stability of such bonds and their sensitivity to hydrolysis as a function of pH values is yet uncertain^83^ and could be analyzed in follow-up simulations of OH^–^ versus OR^–^ attachment.

### 2.5 Prediction of Anisotropic Tensile and Compression Properties

Due to their layered 2D structure, MXene sheets exhibit elastic moduli dependent on the direction and number of MXene layers.^97,98^ We trained the force field to reproduce the experimental in-plane tensile modulus of 330 ± 30 GPa (Table 1 and Figure 2f)^43^ and used it to predict the anisotropic mechanical properties for multiple surface chemistries (Figure 5).

**Figure 5:**
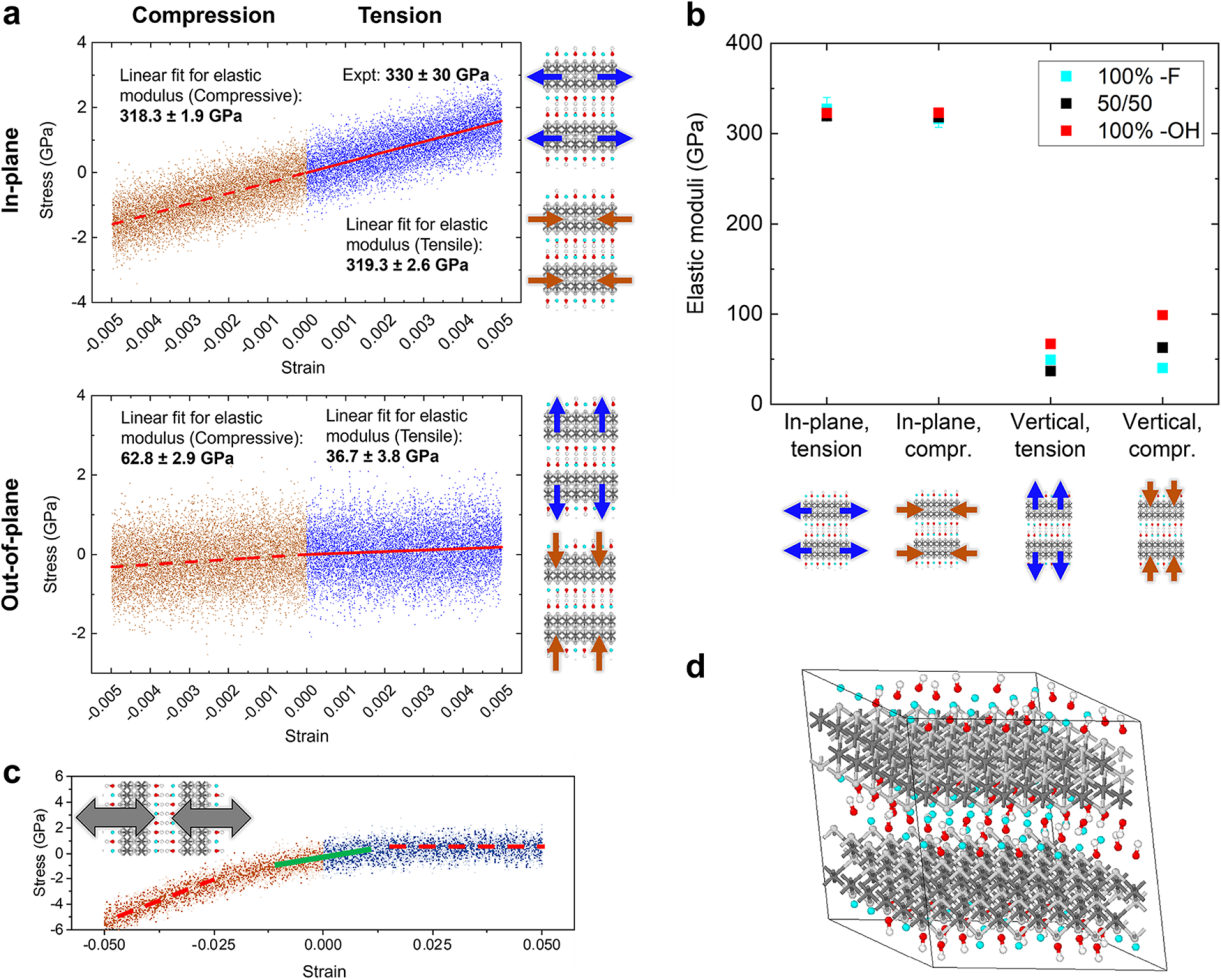
Tensile and compressive mechanical properties of Ti_3_C_2_T_*x*_ MXenes according to IFF MD simulations and experimental data for the in-plane modulus. (a) Stress–strain curves and elastic moduli for two load directions in bilayer MXene with 50/50 hydroxyl/fluorine surface termination, including the experimental reference value.^43^ (b) Dependence of in-plane and vertical out-of-plane moduli on −OH vs −F surface termination. In-plane elastic moduli vary little across surface treatments while vertical moduli are distinctly higher for 100% hydroxyl surface termination, particularly upon compression. (c) Large strain (>1%) for out-of-plane deformation shows nonlinear stress–strain responses. The green line indicates the linear elastic region and red dashed lines indicate higher stiffness upon large compression as well as reduced stiffness upon large tension (beginning layer cleavage). (d) Model of the MXene supercell with OH/F surface termination used in stress–strain simulations.

MXenes have much higher stiffness in the *xy* plane than in the vertical direction due to the covalently bonded Ti–C lattice versus weaker nonbonded interlayer interactions (Figure 5a). For small deformations up to ± 0.5% in-plane, we obtain a linear stress–strain correlation under both tension and compression with a modulus of 319 ± 3 GPa. This value was not contingent on the type of surface termination within the uncertainty (Figure 5b). The vertical, out-of-plane stress–strain curves also follow a linear regime for small deformations up to ±0.5%. Hereby, the tensile modulus for mixed OH, F surface termination was much smaller at 37 ± 4 GPa than the compressive modulus of 63 ± 3 GPa (Figure 5a). Vertical expansion weakens layer–layer interactions while compressive stress leads to interpenetration of the van-der-Waals radii of the surface groups and causes a stiffer response. For large strains beyond 1%, the local linear fit indicates a tensile modulus <5 GPa and a compressive modulus up to 125 GPa equal to layer collision, and failure by amorphization may occur (Figure 5c,d). The out-of-plane elastic responses are also sensitive to the type of surface group, especially upon compression (Figure 5b). The vertical tensile modulus at low strain was 67 GPa for a 100% OH-terminated MXene, 37 GPa for 50/50 OH and F termination, and increased to 49 GPa for 100% F-terminated surfaces. Accordingly, disorder of surface groups considerably weakens the vertical tensional stiffness. There is no direct correlation of the moduli with the cleavage energy (Figure 3b) as the modulus equals the second derivative of the layer interaction energy with respect to distance. The vertical compressive modulus at low strain had the highest value of 99 GPa for 100% OH termination, 63 GPa for 50/50 termination by OH and F, and 40 GPa for 100% F termination (Figure 5b). Accordingly, hydrogen bonds between fully OH terminated surfaces are most resistant to compression, due to the least interlayer space (Figure 2a–e), and mixed compositions still lead to compression of partial hydrogen bonds. Fully F terminated surfaces interact extensively through van-der-Waals forces, which lead to a softer compressive response.

Experimental reference data on anisotropic mechanical properties have been limited and inconclusive regarding out-of-plane moduli (see Section S6, Table S2, and Figure S6 in the Supporting Information).^97,98^ The computed compressive moduli for OH and mixed OH/F termination (Figure 5b) agree with the reported range of values of 80 to 100 GPa for OH, F, and O termination for 40 nm thick MXenes by Firestein et al.^97^ Fully F-covered surfaces were unlikely to form in experiments, and higher reported values relate to 5 μm thick samples.^98^ These samples may include stacking defects. DFT calculations on small unit cells reported in-plane elastic moduli from 256 GPa^15^ to 485 GPa,^44^ and values for vertical moduli have not been reported to-date (Table S2 in the Supporting Information).

### 2.6 Estimation of Shear Properties and Shear Failure

Shear slip between layers is the primary failure mechanism in 2D materials such as MXenes, graphene and clay minerals.^99–102^ The activation energy for shear failure is low as no covalent bonds are broken. Limitations in laboratory measurements of interlayer shear properties provide an opportunity to quantify these processes by simulations (Figure 6).

**Figure 6:**
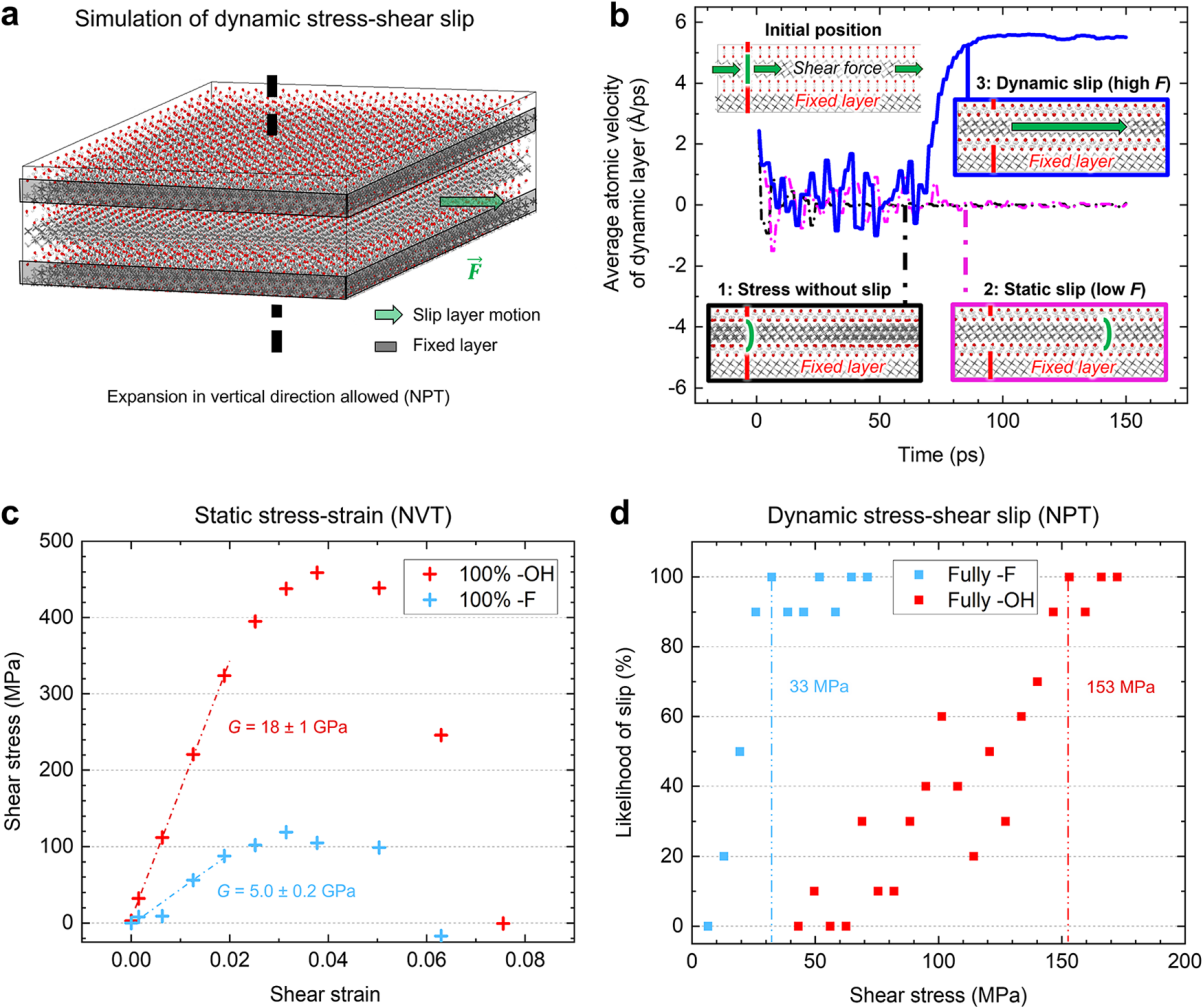
Estimation of shear properties in multilayer Ti_3_C_2_T_*x*_ MXene via IFF MD simulations. (a) Model setup to examine dynamic shear slip of a mobile MXene layer sandwiched between layers with fixed atomic positions and mobile surface groups. (b) The shear rate (slip) as a function of time for different layer pulling forces *F*. Scenario 1 describes linear elastic deformation without slip, Scenario 2 static slip, and Scenario 3 dynamic slip with a steady-state shear rate. (c) Shear stress-shear strain curves up to failure, showing a static shear strength of 460 and 120 MPa for full OH and F termination, respectively. The calculations involve small layer displacements, yield a “barrier” strength, and strains >0.05 lead to interlayer reconstruction. (d) Elucidation of the effective shear strength from dynamic sliding simulations. Steady-state slip of fully hydroxylated (−OH) surfaces develops between 50 and 153 MPa. Fully fluorinated (-F) surfaces dynamically slip between 15 and 33 MPa (at the higher values, the MXene layer always slid). >10 independent simulations for each OH and F termination were carried out to estimate the average shear strengths and associated uncertainties.

We utilized a sandwich model with a MXene layer with fixed internal positions and flexible surface groups next to an adjacent mobile MXene layer in a 3D periodic structure (Figure 6a) to ramp up shear forces and monitor the resulting slip regimes including shear strain and shear rate (Figure 6b). The layers were allowed to vertically expand and contract in the process to mimic layer mobility comparable to shear experiments. A reversible elastic response (stress without slip) was obtained when the applied shear stress was small, and the average atomic velocity over time (= shear rate) remained zero (Scenario 1 in Figure 6b). At higher shear force, static slip occurred and lead to stepwise layer displacements, i.e., a “plastic” deformation (Scenario 2). The shear force then created temporary nonzero velocities of the dynamic layer, however, without a sustained sliding motion. Further increase of the shear stress finally led to dynamic slip (Scenario 3 in Figure 6b). The flexible layer then reached a steady state sliding velocity (i.e., shear rate).

This dynamic assessment of shear moduli and sliding regimes was sensitive to the applied forces. For example, small forces could lead to negligible strain and just slightly higher applied forces could cause the dynamic MXene layer to slide 1 to 3 Å into the next stable configuration and relax the shear stresses. Therefore, we employed an additional method to study static shear stress-shear strain curves (Figure 6c). This protocol involved small stepwise deformations of a 3D periodic 2-layer MXene model (Figure 5d) by less than 1 Å (strain <0.10) and tracking changes in shear stresses under NVT conditions, i.e., not allowing layer expansion or contraction (Figure 6c). The resulting static shear stress-shear strain curves indicate an initial linear elastic response up to a shear strain of about 2% before static or dynamic slip occurs (Figure 6c). The associated shear modulus *G* strongly depends on the surface termination, amounting to 18 GPa for 100% OH termination versus 5.0 GPa for 100% F termination. Accordingly, hydrogen bonds across the interlayer space increase the shear stiffness of OH-terminated MXene by three times compared to predominant van-der-Waals interactions in fully fluorinated MXene (Figure 6c). The stress–strain curves also indicate a transition into plastic shear deformation past 2% shear strain and a “static” shear strength of ∼460 MPa and ∼120 MPa, respectively. For larger deformation, a drop in shear stress is observed and the MXene layers reach new stable conformations with relaxed surface terminal groups (see further information in Section S7 and Figure S7 in the Supporting Information).

The analysis of shear failure was then continued by the dynamic model (Figure 6a,b,d) by increasing the shear stress (and shear force) in the simulations and monitoring the corresponding layer motion at 298 K under NPT conditions (Figure 6d). This protocol identified the ranges of “dynamic” shear stress that increase the probability of slip from 0% to 100%. For fully fluorinated MXene surfaces, a dynamic shear stress of ≥15 MPa induced discontinuous slip, and 33 to 50 MPa induced dynamic slip (Figure 6d). In contrast, the fully hydroxylated surfaces required ∼50 MPa to initiate discontinuous slip and 150 to 180 MPa for dynamic slip. Accordingly, dynamic shear failure would require shear forces of ∼150 MPa for 100% OH termination and ∼30 MPa for 100% F termination. The “static” shear strength of 460 and 120 MPa under fixed shear strain (Figure 6c) represents an upper bound under constant volume.

In summary, the dynamic shear strength for the onset of sliding under constant pressure is 15 to 30 MPa for the fully fluorinated (-F) MXene and 50 to 155 MPa for the fully hydroxylated (−OH) MXene. While strongly dependent on surface groups and associated interlayer cohesion, the shear strength remains much lower than the in-plane tensile strength. Simulations with IFF and IFF-R are suited for systematic exploration of shear properties for other surface treatments, including bridging ligands that covalently connect adjacent MXene layers. The results can be utilized in modeling the mechanics of MXene-based composites up to the macroscale using FEM, DEM, and other higher level approaches.^103^

### 2.7 Nanoindentation and Failure Mechanism

Nanoindentation of MXenes using Atomic Force Microscopy (AFM) has been previously carried out to characterize force–displacement curves, fracture depth, and the in-plane modulus of ∼330 GPa.^43^ MD simulations, especially with the reactive IFF-R parameters,^32^ enable the reproduction of AFM experiments in all-atom resolution (Figure 7). The setup included MXene monolayer coverage of a pore plate as in experiment, including flexible circular cavities with 15, 60, and 170 nm diameter and fixed atoms in outer regions (Figure 7a,b). The tip material and shape can be customized. We chose a model of a diamond AFM tip with a 30° cone shape terminated by a single carbon atom. Computed force–displacement curves are dependent on the size of the open pore and agree well with the experimental AFM data, which utilize a larger pore size of 820 nm (Figure 7c).^43^ For a diameter of 15 nm, small displacements generate deflection angles larger than in experiments and, correspondingly, higher forces. The agreement of computed force–displacement curves with AFM data is very good for 60 and 170 nm pore diameters (Figure 7c).

**Figure 7:**
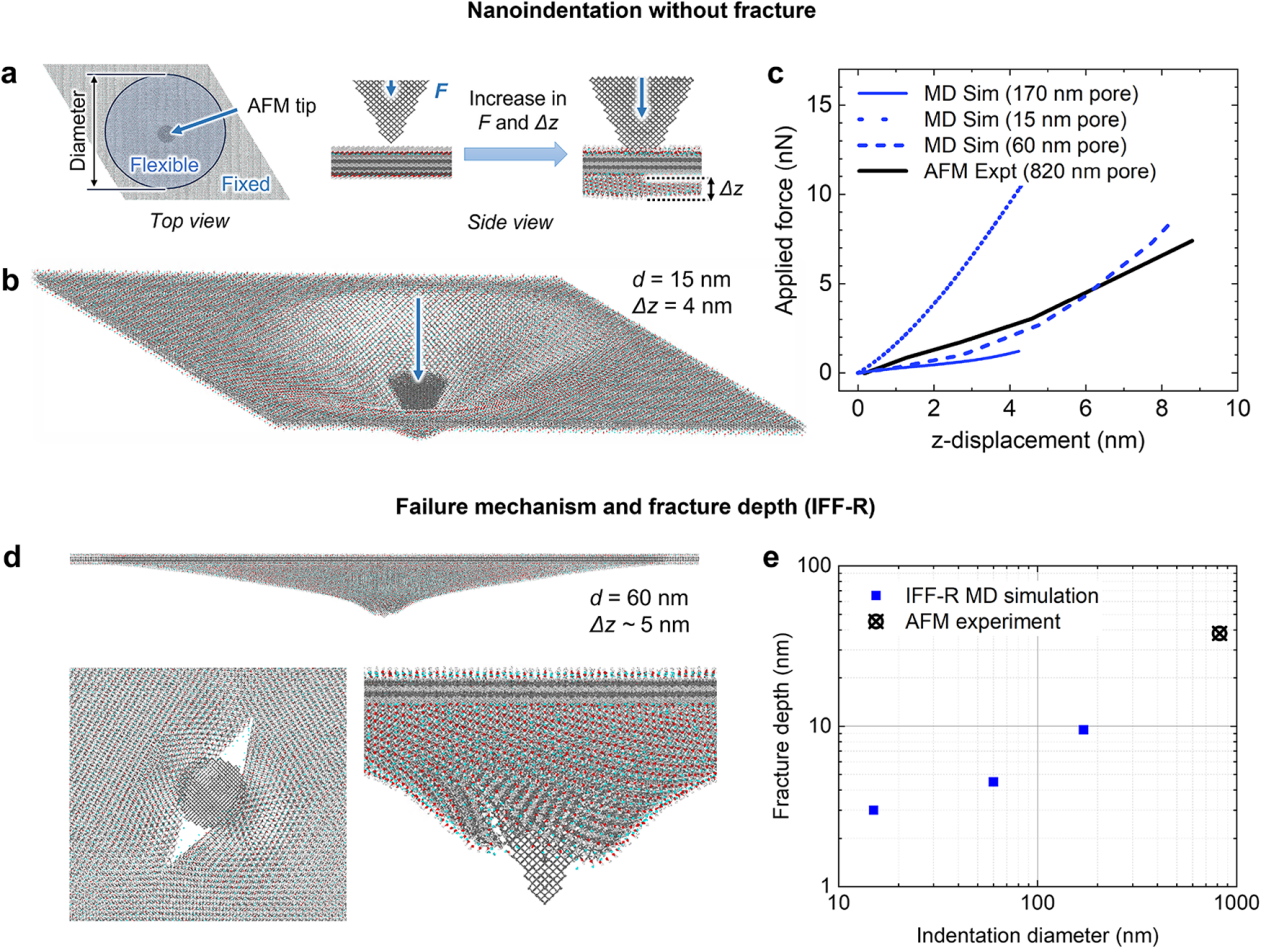
Nanoindentation and failure analysis of Ti_3_C_2_T_*x*_ MXenes via IFF-R MD simulations and comparisons to AFM measurements. (a) The setup for nanoindentation MD simulations involved a constrained MXene monolayer with a circular flexible region, similar to the plate-and-pore setup in AFM experiments. The AFM tip was generated by trimming a diamond cubic lattice into a cone with a 30° tip angle, and the tip was initially placed 4 Å above the center of the MXene monolayer. (b) Visualization of an indent (without bond breaking). (c) Force–displacement curves from MD simulations are in good agreement with AFM measurements,^43^ including a steeper slope for small pore diameter (15 nm). (d) Reactive IFF-R simulations indicate brittle failure with crack formation and propagation, consistent with macroscale experiments.^104^ (e) Fracture depth vs indentation diameter. Simulation results for small diameters of 15, 60, and 170 nm extrapolate well to experimental data for 820 nm pore diameter.^43^ Force–displacement and fracture results are 20× more accurate with IFF-R than prior ReaxFF models,^24^ enabling predictive simulations for complex setups and geometries.

Reactive simulations with IFF-R allow to further monitor the failure mechanism (Figure 7d) and the fracture depth (Figure 7e). At penetration depths of multiple nm, the Ti–C bonds break and form cracks that propagate with increasing penetration. We observed brittle failure in the simulations, consistent with recent experiments.^104^ Broken covalent bonds (Ti–C) do not reconnect, consistent with known failure mechanism for carbides and ceramics, and predominantly electrostatic bonds (Ti–OH, Ti–F) often reconstruct in a disorderly fashion once dissociated.^71^ The fracture depths in MD simulations are in very good agreement with AFM measurements, predicting a consistent trend from smaller sizes in simulation (15, 60, 170 nm) to 820 nm in experiments (Figure 7e). The fracture depth increased from 2 to 40 nm as the pore diameter increases, and the corresponding MXene deflection angles at fracture decreased from 22° for 15 nm diameter to 6° at 170 and 820 nm diameter (Section S8 in the Supporting Information, including limitations and possible extensions).

In comparison, recent simulations of MXene nanoindentation with ReaxFF MD^24^ reported forces of 20 nN at 1 nm displacement that are 20× higher than ∼1 nN in experiment (Figure 7c), and also indicated notable ductility instead of brittle failure. The ReaxFF findings thus do not align well with experiments^43,104^ and point to weaknesses in the ReaxFF model.^24^

The IFF-R models can be utilized to predict load–displacement curves and fracture depth for various multilayer and polymer composite geometries including defects.

### 2.8 Analysis of Nanoscale Thermal Conductivity

Ti_3_C_2_T_*x*_ MXenes have low thermal conductivity, which can be attractive in combination with electrical conductivity and high mechanical strength.^45,105,106^ We computed the in-plane and out-of-plane thermal conductivities as a function of surface termination (Figure 8a,b), including the role of defects.^107^ The results are in close agreement with experimental measurements and reveal design trends (Figure 8c–e).

**Figure 8:**
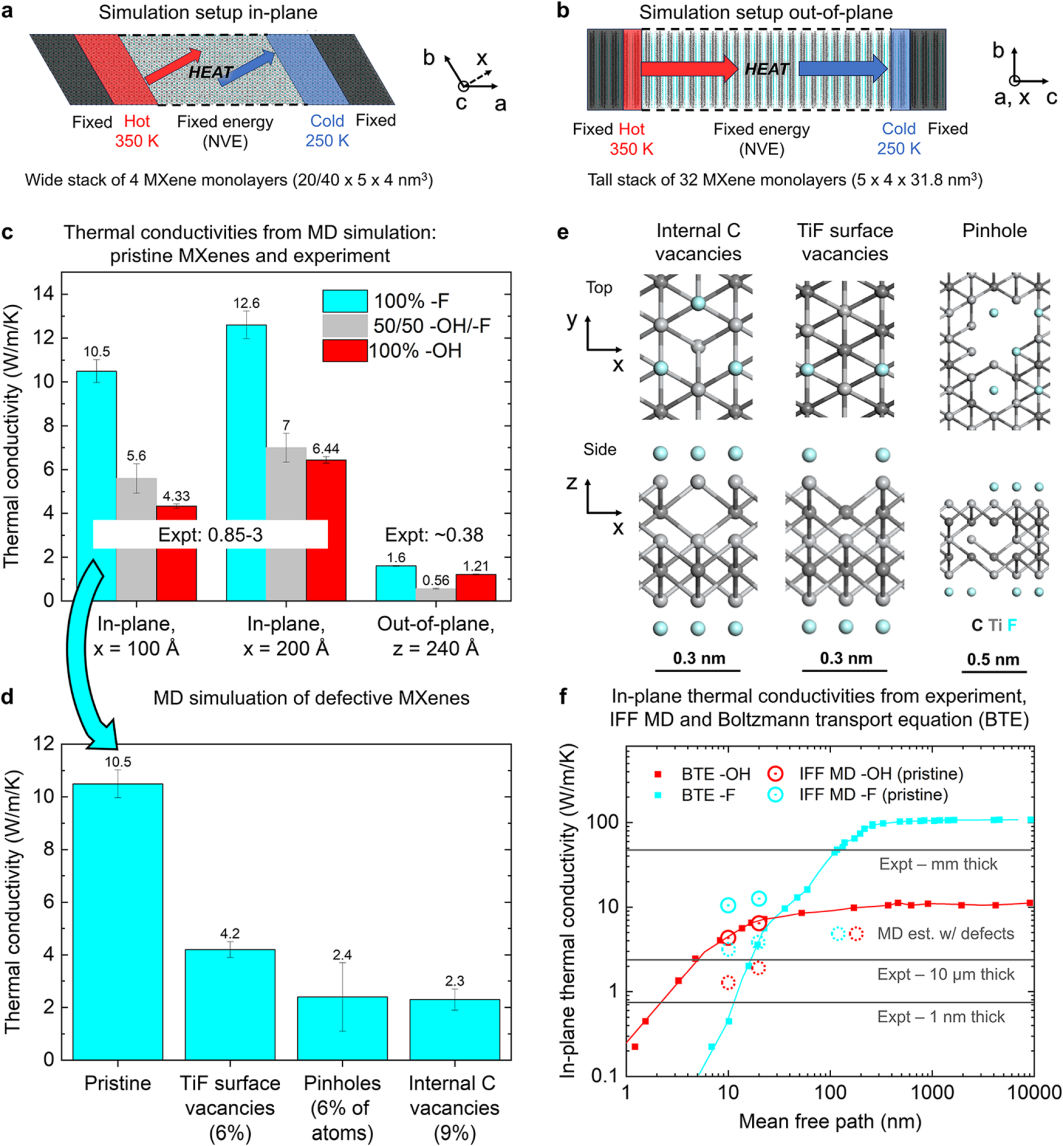
Prediction of nanoscale thermal conductivity in Ti_3_C_2_T_*x*_ MXenes as a function of surface groups and defects by MD simulation in comparison to experimental data and the iterative Boltzmann transport equation (BTE). (a, b) Visualization of the model setups to compute the in-plane and out-of-plane heat flow. Fixed temperatures were maintained at 350 and 250 K in the outer hot and cold slabs, respectively. MD simulations were carried out in the NVE ensemble to form a steady-state thermal gradient in the main slab of length *x* and *c*, respectively, to calculate the thermal conductivity. (c) Computed thermal conductivities in-plane and out-of-plane for three surface terminations in pristine MXene layers, including two different slab lengths for the in-plane values. The in-plane conductivity of 100% F-terminated MXenes was about twice as high as for 100% OH and 50 OH/50 F mixed termination. Out-of-plane conductivities were much lower, with lowest values for 50/50 mixed surface terminations. The results are consistent with experimental measurements^45^ considering the absence of defects. (d, e) Impact of common defects on the simulated in-plane thermal conductivity for fully fluorinated MXene. The three types of defects in (e) lower thermal conductivities by a factor of 2.5 to 4 and lead to excellent agreement with experimental measurements.^45^ Internal C vacancies have a stronger effect than vacancies of TiF surface groups. The decrease in thermal conductivity correlates with the percentage of atoms removed under consideration of stoichiometry and charge neutrality. (f) Comparison of results from the iterative Boltzmann Transport Equation (BTE) for monolayers^47^ with in-plane IFF MD thermal conductivities for multilayers and experimental data (horizontal lines for multiple thicknesses).^45^ The BTE method (1 nm thickness) tends to overpredict experimental values^45^ at mean free paths larger than 10 nm up to 1 order of magnitude. MD results are closer to laboratory observations, and dotted circles indicate estimates for multilayers with defects.

The in-plane thermal conductivity for pristine MXenes of 4-layer thickness and 10 to 20 nm length was computed between 6 and 13 W m^–1^ K^–1^, whereby the OH-substituted MXene had the lowest values around 6 W m^–1^ K^–1^, a 50/50 OH/F terminated MXene a slightly higher value of 7 W m^–1^ K^–1^, and an F-terminated MXene 13 W m^–1^ K^–1^ (Figure 8c). A modest increase of 20–50% in thermal conductivity was observed when increasing the system length from 10 to 20 nm, consistent with the inclusion of longer-wavelength phonon modes. The inclusion of common defects^107^ drastically reduced heat conduction, as tested for 100% F covered surfaces at 10 nm length (Figure 8d,e). 9% of randomly missing carbon atoms in the internal TiC layer lowered the thermal conductivity from 10.5 to 2.3 W m^–1^ K^–1^, showing excellent agreement with experimental data. A similar reduction was seen by pinhole defects (6% of total atoms), and less reduced thermal conductivity of ∼4 W m^–1^ K^–1^ was obtained for 6% missing superficial TiF groups (Figure 8d,e).

Experimental data for the in-plane thermal conductivity exhibit a strong dependence on film thickness, starting at 0.85 W m^–1^ K^–1^ for near-monolayers^45^ to 2.84 W m^–1^ K^–1^ for a 10 μm thick film^105^ and up to 55.8 W m^–1^ K^–1^ for 3 mm thick flakes (Figure 8d).^106^ Laboratory samples typically include a mix of defects across scales such as missing C atoms, surface groups, pinholes (Figure 8e), grain boundaries, broken ends, layer twists, and residual solvent, leading to lower thermal conductivities than for pristine MXenes. Surface terminations were not controlled and likely a mix of OH, F, or -O- groups. Computed in-plane thermal conductivities near 7 W m^–1^ K^–1^ for pristine MXenes of multilayer thickness with mixed surface termination appear to be quite accurate in comparison to experimental data of 0.85 to 3 W m^–1^ K^–1^ for monolayers to multilayers containing common defects (Figure 8c,d and Table 1).^45^ DFT and DFT/ML calculations reported values of 108 ^47^ and 96.4 W m^–1^ K^–1^.^46^ These results are nearly 2 orders of magnitude larger than measurements and less consistent with experimental observations than IFF MD values.

Out-of-plane thermal conductivities of pristine MXenes from MD simulations are highest at 1.6 W m^–1^ K^–1^ for fully F terminated MXenes, lowest at 0.56 W m^–1^ K^–1^ for OH/F mixed MXenes, and again higher at 1.21 W m^–1^ K^–1^ for fully OH-terminated MXenes (Figure 8c). Accordingly, heat transport across a stack of MXene layers is sensitive to the thickness of the interlayer and the nonbonded forces that translate thermal motion. The computed out-of-plane thermal conductivities of pristine OH/F mixed MXenes of 0.56 W m^–1^ K^–1^ agree well with experimental measurements of ∼0.38 W m^–1^ K^–1^ (Figure 8c).^45^ Defects in real laboratory samples such as vacancies, intercalated ions, or layer twist are expected to increase the effective gap distances and lower the thermal conductivity. Therefore, values of ∼0.56 W m^–1^ K^–1^ for pristine MXenes are in excellent agreement with measurements of ∼0.38 W m^–1^ K^–1^.

The experimental and computational data indicate that mechanisms of thermal transport differ in-plane and out-of-plane. The in-plane thermal conductivity is multiple times larger due to thermal vibrations throughout the Ti–C lattice. Hereby, monatomic F termination supports about twice as much momentum and heat transfer along parallel layers compared to diatomic OH termination via a more orderly interlayer structure with coordinated vibrations. Increasing OH termination, such as 50/50 OH/F and 100% OH, lowers the in-plane conductivity due to random orientations, less coordinated vibrations, and hydrogen bonds that diffuse some thermal energy vertical to the layers (Figure 8c). For heat transport vertically to the layer direction (out-of-plane), monatomic F termination also leads to highest conductivity as the atoms in regular surface positions transfer heat more effectively. The out-of-plane thermal conductivity is approximately 25% lower for OH-terminated MXene where the diatomic OH groups exhibit orientational disorder and mainly interact through hydrogen bonds across the interlayers in various directions, resulting in less effective heat transfer. The lowest vertical heat transmission was observed for mixed OH/F terminations (Figure 8c), associated with maximum surface disorder, fewer H bonds across the interlayer, and more randomized transport paths. Pairing OH terminal groups with F terminal groups across the interlayer thus weakens out-of-plane momentum transfer.

A prior semiclassical model to describe in-plane thermal conductivities in monolayers is the iterative Boltzmann Transport Equation (BTE) (Figure 8f).^47^ BTE-based modeling combines phonon frequencies and scattering rates from DFT (PBE) simulations with iterative solutions of the nonequilibrium phonon population to predict in-plane thermal conductivities with long phonon free paths. The BTE results suggest that MXenes terminated by OH groups are more conductive than MXenes terminated with F groups for mean free paths up to ∼ 30 nm, and F terminations become more thermally conductive than OH terminations for larger mean free paths (Figure 8f).^47^ The gap in BTE results between F and OH terminated surfaces grows very large at >100 nm mean free path. However, being applicable only to idealized monolayers (1 nm thick), the BTE results^47^ are substantially larger than experimental measurements of ∼1 W m^–1^ K^–1^,^45^ indicating in-plane thermal conductivities of ∼105 W m^–1^ K^–1^ for F terminations and ∼11 W m^–1^ K^–1^ for OH terminations (Figure 8f, Section S9 in the Supporting Information). The magnitude of the discrepancy cannot be readily explained by defect concentration alone.

In summary, predictions of thermal transport for pristine and defective MXenes using IFF MD show excellent agreement with available experimental data. This level of agreement exceeds that of DFT and BTE approaches, while relying on simpler workflows and significantly lower computational cost. The MXene compositions and defects studied here (Figure 8c–e) can be further expanded in future work (Section S9 in the Supporting Information). Extending system sizes beyond the phonon mean free path (Figure 8a,b) toward micrometer scales will enable direct simulation of macroscale transport and phononic crystal effects.^108^

### 2.9 Application to Other MXene Chemistries and Limitations

The models and methods can be extended to other surface terminations, defects, pH values, solvent, biomolecular, and inorganic interfaces due to compatibility with IFF, CHARMM, AMBER, CVFF, OPLS-AA, PCFF, and COMPASS parameters (see refs 31–33 ). For example, we can include surface terminations like bridging oxygen (-O−) by locally replacing pairs of OH groups (−0.5e effective charge each) with one -O- group with a charge to −1.0*e* for electroneutrality. The chemistry of the main M_*n*+1_X*_n_* phase and T_*x*_ surface groups can be more extensively modified using the IFF workflow, existing parameters for other metals, carbides, nitrides, and functional groups, and further X-ray crystal structure data. Chemical bonding and physically realistic ranges of parameters will be similar to existing validated chemistry space in IFF and CHARMM (Table 2).

As a limitation, IFF neglects the full electronic structure. Electronic structure details and investigations into reaction mechanisms require QM/MM or separate quantum mechanical simulations, preferably at the CCSD­(T) level, along with experimental validation or analogy. IFF­(-R) parameters can include more detailed representations of local electron density when needed,^49,109^ and such extensions are most effective when guided by interpretability, high-level quantum mechanical insights, and the standard workflow based on validation against experimental data from the atomic scale to the macroscale (Figure 2f). The models rely on simplified interatomic interactions that can be represented in common all-atom energy expressions for nonreactive and reactive molecular simulations. Regarding thermal conductivity, uncertainties in MD simulations include missing electronic contributions for in-plane transport. These contributions are partly compensated for by interlayer Ti–C bonds with validated vibration frequencies (Figure S3 in the Supporting Information and Section S10 for further discussion of trade-offs and opportunities^110^).

## 3 Conclusion

We present a transferable and validated force field for MXenes, Ti_3_C_2_T_*x*_, with flexible surface terminations (−OH, -F), compatibility with solvent and polymer mixtures, and extensibility to other chemistries. The model is trained on atomic-level chemical knowledge following the INTERFACE Force Field (IFF/IFF-R) workflows and fine-tuned to reproduce lattice parameters, Raman spectral peaks, contact angles for one known surface chemistry, and in-plane Young’s modulus according the experimental data. The model then predicts layer cleavage energies, contact angles of water and diiodomethane, directional tensile elastic moduli, shear moduli, shear mechanisms, shear strength, nanoindentation and failure, as well as directional thermal properties for a wide range of −OH/-F surface chemistries in excellent agreement with available experimental data. We explain aqueous surface chemistry and the mechanism of dopamine binding toward sensor applications. The model is part of IFF, IFF-R, and compatible with other major force fields for molecular dynamics simulations (CVFF,^50^ CHARMM,^53^ DREIDING,^51^ AMBER,^54^ and OPLS-AA^55^) that add functionality for organic and biological materials. The accuracy, versatility, and low computational cost exceed the capabilities of prior modeling techniques. Namely, the ability to incorporate a variety of liquids, pH dependent surface chemistry, surface ligands, polymers and inorganic solids without added parameters enables property predictions of billions of unknown MXene hybrid materials at scales up to micrometers.

We identified significant differences up to 2x in surfaces energies, contact angles, and out-of-plane moduli related to different surface functional groups and cross-layer interactions. Interlayer shear moduli amount to 5 GPa (-F) and 18 GPa (−OH), with shear strengths (onset of slip) of 10 and 50 MPa, and fully dynamic slip at about 33 and 153 MPa, respectively, suggesting major stability gains from higher −OH termination in composite materials. Computed force–displacement curves in nanoindentation and fracture depths for MXene layers up to 170 nm diameter correlate closely with AFM experiments and reveal a brittle failure mechanism. The accuracy is clearly higher than prior reactive simulations and suited for predictions of failure of MXene-based materials under directional loads. Predicted in-plane thermal conductivities illustrate a large impact of common defects and excellent correlation with experimental data. Pristine MXenes have 2.5x to 4x larger in-plane thermal conductivity compared to structures with common levels of defects (few percent of atoms). -F termination leads to about 2x the in-plane thermal conductivity compared to mixed -F/–OH or −OH termination. Out-of-plane thermal conductivities are also in very good agreement with experiment, showing ∼0.6 W m^–1^ K^–1^ for −OH/-F mixed surfaces, ∼1.2 W m^–1^ K^–1^ for 100% -F termination, and ∼1.6 W m^–1^ K^–1^ for 100% −OH termination.

Dopamine adsorption on MXene surfaces was characterized by QCM-D and UV/vis measurements and IFF MD simulations. The binding mechanisms involved displacement of superficial water in coplanar and perpendicular orientations with about −10 kcal/mol binding energy. Assembly into multilayers led to a mix of flat-on and tilted orientations. Follow-up studies as a function of electrolyte, pH values, incorporation of polymers, proteins, and DNA are feasible using integration of IFF into CHARMM, CVFF, OPLS-AA, and other force fields.^31,33^

Our models provide a platform for nanoscale property predictions in Ti_3_C_2_T_*x*_ MXenes and organic composite materials, extensible to other surface chemistries and covalent surface modifications, chemical reactions, and MXene chemistries based on nitrogen and other transition metals. We expect utility in screening complex materials for structural, sensor, catalytic, thermal, and multifunctional applications.

## 4 Computational and Experimental Methods

A concise overview of the methods is provided here. A complete description is available in Section S10 in the Supporting Information.

### 4.1 Model Construction and Force Field Development

3D periodic all-atom models of Ti_3_C_2_T_*x*_ MXenes were constructed using crystallographic coordinates from X-ray diffraction (space group *P*6_3_/*mmc*).^34^ Surface terminations were represented by statistically distributed −OH and −F groups (typically 47%/53%) and varied as needed from 0–100% −OH coverage. Force field parameters were developed for the INTERFACE Force Field (IFF) and its reactive extension (IFF-R), following established workflows for the assignment of atomic charges, Lennard-Jones parameters, and bonded terms (Figure 2).^31^ Charges were determined using the Extended Born approach^61^ and refined to reproduce water and diiodomethane contact angles, while bonded constants were adjusted to match X-ray bond lengths, vibrational spectra, and the in-plane elastic modulus. For IFF-R, harmonic Ti–C bonds were replaced with shifted Morse potentials to enable bond dissociation while retaining compatibility with CHARMM, AMBER, OPLS, and related fixed-charge force fields.^32^ All parameters were verified to be interpretable and consistent with those for similar chemistries in IFF.

### 4.2 Molecular Dynamics Simulations

Simulations were performed primarily using LAMMPS, as well as NAMD for biomolecular adsorption, and Materials Studio (Discover) for test runs. Unless otherwise stated, systems were equilibrated at 298.15 K and 1 atm using 1 fs timesteps, 12 Å Lennard-Jones cutoffs, and PPPM electrostatics with high accuracy of 10^–4^ to 10^–6^. Equilibration employed NPT ensembles followed by production runs in NPT, NVT, and NVE ensembles as appropriate for each property, and typical simulation times were between 10 and 50 ns. Thermostats involved Nose–Hoover chains or velocity rescaling, and barostats Nose-Hoover chains or the Parrinello–Rahman method. System sizes ranged from 6 × 6 × 1 to >600 × 600 × 1 supercells, equal to side lengths of 2 to 200 nm, with thickness and lateral dimensions selected to ensure negligible finite-size effects. Uncertainty estimates were obtained through block averaging and independent replicate simulations.

### 4.3 Structural and Interfacial Properties

Lattice parameters, density, and bond lengths were obtained from MD simulations in equilibrium using the NPT ensemble and compared directly with experimental X-ray data. Cleavage energies were determined as a difference between equilibrium energies of bulk MXene stacks and equivalent slabs with added vacuum, normalized by surface area. Equilibrium contact angles of water (TIP3P or flexible SPC model) and diiodomethane were computed using droplet-on-surface simulations combined with analytical relations between droplet center of mass and macroscopic contact angle.

### 4.4 Mechanical Properties and Nanoindentation

Elastic moduli were determined from linear fits to stress–strain responses obtained under applied in-plane and out-of-plane tension and compression. The interlayer shear response was evaluated using both static shear deformation and dynamic sliding protocols, enabling determination of shear modulus, static slip thresholds, and dynamic shear strength. Nanoindentation simulations used rigid diamond cones positioned above supported MXene membranes with circular free regions (15–170 nm in diameter). Force–displacement curves and fracture behavior were evaluated using both nonreactive IFF and reactive IFF-R simulations.

### 4.5 Thermal Conductivity

In-plane and cross-plane thermal conductivities were computed using nonequilibrium MD simulations. Hot and cold slabs at 350 and 250 K, respectively, were created at opposite boundaries, while the central region was analyzed in steady-state in the NVE ensemble. The resulting linear temperature gradients and steady-state heat fluxes were analyzed to calculate thermal conductivity via Fourier’s law.

### 4.6 Biomolecular Adsorption and Experimental Validation

Dopamine adsorption was studied experimentally using QCM-D and UV–Vis spectroscopy and computationally using IFF-CHARMM36 parameters for dopamine with explicit water. pH-dependent surface protonation was included using data on surface charge from experiments. Binding enthalpies, residence times, and preferred orientations were obtained from simulations over long time sclaes and compared with QCM-D uptake and UV–Vis signatures.

## Supplementary Material





## Notes

Prior MXene characterization and simulation (S1); IFF/IFF-R energy expression and parameters (S2); vibrational spectra (S3); contact angles and surface energy (S4); dopamine binding (S5); elastic properties (S6); shear properties (S7); nanoindentation (S8); thermal conductivity and discussion of defect models (S9); detailed computational and experimental methods (S10); IFF/IFF-R energy terms (Figure S1); MXene structural detail and atom types (Figure S2); Raman/IR comparison (Figure S3); contact angles (Figure S4); dopamine binding regimes (Figure S5); extended elasticity (Figure S6); shear-response independence (Figure S7); complete IFF/IFF-R Ti_3_C^_2_^T^_x_^ MXene force field parameters for multiple force fields (Table S1); elastic-moduli compilation (Table S2) (PDF)
IFF and IFF-R force field for MXenes, 3D models of MXenes and MXenes with defects ready for simulation, examples of the calculations reported, run scripts and analysis scripts (ZIP)
