# Supplementary material for: Validated Reactive Force Field Quantifies MXene Interfacial Properties, Mechanics, and Thermal Transport
Source: ACS Nano. 2026 Jul 22;20(30):20979–97. doi: 10.1021/acsnano.5c20046 (PMC13450432; doi:10.1021/acsnano.5c20046)
Supplement: Supplementary file 1 [file nn5c20046_si_001.pdf]

Supporting Information

for

Validated Reactive Force Field Quantifies MXene Interfacial Properties, Mechanics, and Thermal Transport

Isaac W. Armstrong,[1, 2] Joseph M. Slocik,[3] Leo Beck,[1, 2] Kennedy Brown,[3] Christopher Muratore,[4] Dhriti Nepal,[3] Cheng Zhu,[2] Vikas Varshney,[3*] Hendrik Heinz[1, 2*]

[1] Materials Science and Engineering Program, University of Colorado, Boulder, Boulder, CO 80309

[2] Department of Chemical and Biological Engineering, University of Colorado, Boulder, Boulder, CO 80309

[3] Materials and Manufacturing Directorate, Air Force Research Laboratory, Wright-Patterson Air Force Base, OH 45433

[4] Department of Chemical and Materials Engineering, University of Dayton, Dayton, OH 45469

[*] Corresponding authors: vikas.varshney.2@afrl.af.mil, hendrik.heinz@colorado.edu

This file contains:

Sections S1-S10:

S1. Prior MXene Characterization and Simulation: Brief overview of MXene structure, anisotropy, surface chemistries, limitations of prior DFT/MD models, and the need for a validated interpretable IFF/IFF-R framework.

S2. IFF/IFF-R Energy Expression and Parameters: Description of IFF potential (Class I), Extended-Born charges, bonded and LJ parameter optimization, shifted-Morse reactive bonds; complete parameter sets for $Ti_3C_2T_x$ (Table S1); workflow details; use of IFF with multiple energy expressions

S3. Vibrational Spectra: VACF/FFT protocol for Raman/IR calculations and agreement of Ti–C and angle-bending modes with experiment (Figure S3).

S4. Contact Angles and Surface Energy: Experimental variability and pH sensitivity; MD droplet methods; connection to Young/OWRK formulations; representative contact-angle fluctuations and dependence on droplet size in simulations (Figure S4).

S5. Dopamine Binding: Definitions of attached, nearby, and detached regimes used for adsorption analysis (Figure S5).

S6. Elastic Properties: Comparison of experimental, DFT, and MD elastic moduli; benchmark selection; extended stress–strain results for different surface terminations (Table S2, Figure S6).

S7. Shear Properties: Static and dynamic shear protocols; independence of orthogonal shear components; onset of slip and friction-related comparisons (Figure S7).

S8. Nanoindentation: Reactive and nonreactive simulations across multiple pore sizes; validation against AFM fracture measurements.

S9. Thermal Conductivity and Defect Models: Non-equilibrium steady-state method, length effects, comparison with BTE/DFT, discussion of defect models, experimental variability and anisotropy.

S10. Detailed Computational and Experimental Methods: Comprehensive protocols for model construction, force-field development, MD settings, lattice/density, Raman/IR, cleavage energy, contact angles, dopamine adsorption (QCM-D, UV–Vis, MD), elasticity, shear, nanoindentation, and thermal transport; tradeoffs in FF development; availability of parameters, input files, and scripts (Sections S10.1–S10.15).

Supporting References

Figures: S1–S7: S1 (IFF/IFF-R energy terms), S2 (MXene structural fragment), S3 (Raman/IR comparison), S4 (contact angles), S5 (dopamine binding regimes), S6 (extended elasticity), S7 (shear-response independence).

Tables: S1–S2: S1 (full $Ti_3C_2T_x$ IFF/IFF-R parameters), S2 (elastic-moduli compilation).

Supporting Files (separate part of SI): IFF/IFF-R parameter sets, 3D MXene bulk and surface models, LAMMPS/NAMD input files, MatLab/Python/OVITO analysis scripts, sample data.

S1. Prior MXene Characterization and Simulation

S1.1. MXene Synthesis and Characterization. 2D MXene nanosheets, or nanolayers, are synthesized by etching and exfoliation of three-dimensional MAX phases such as Ti_3AlC_2 to remove group 13 or 14 ("A") elements. MXenes have strong polar covalent bonds within individual 2D layers, especially within the carbide framework, whereas inter-layer interactions between two MXene layers are much weaker and mediated by non-bonded interactions including hydrogen bonds. Consequently, like other 2D materials, MXenes exhibit a high anisotropy between in-plane and out-of-plane properties. For example, MXenes have lubricating properties due to low shear resistance between layers and various tunable surface modifications (while etching or post-etching) can modulate the degree of anisotropy in mechanical properties towards tailored applications.

S1.2. Structure and Nanoscale Properties of $Ti_3C_2T_2$ MXenes. Our simulations focus on $Ti_3C_2T_2$, the first stable MXene discovered, and the introduced IFF/IFF-R parameters are scalable to similar chemistries. $Ti_3C_2T_x$ layers can be written as $T_{x/2}$-Ti-$C_{0.5}$($C_{0.5}$Ti$C_{0.5}$)$C_{0.5}$-Ti-$T_{x/2}$ (for x = 2) and exhibit electrical conductivity with common surface groups (T = F, OH) that adds to multifunctional properties when used as a reinforcing additive in composite materials.[1] The structural stability of $Ti_3C_2T_x$-based MXenes is limited due to low shear strength and delamination. However, the exploration of different surface functionalities, better understanding of MXene-polymer interfaces and interlayer MXene bridging via covalent or electrostatic links is likely to broaden the applicability in composites.[1]

The experimental characterization of organic assembly and interlayer shear properties remains challenging due to the nanoscale dimensions of MXenes. These difficulties provide opportunities for computational modeling in atomic resolution, especially when the simulation methods can

account for surface functionality, electrolyte and polymer interfaces, as well as mechanical properties. Molecular dynamics simulations with the INTERFACE force field (IFF) can provide such high-fidelity screening as they account for surface modification, solvent and (bio)polymer interfaces,[2] and exceed the accuracy of density functional theory (DFT) at orders of magnitude lower computational cost.[3-5]

S1.3. The INTERFACE Force Field and Prior Simulation Studies. IFF is a force field that collects chemical knowledge for consistent simulations of compounds across the periodic table, and has a track record of compatibility with organic and biomolecular force fields.[2] IFF covers metals, oxides, minerals, gases, solvents and polymers, as well as 2D materials such as graphene[6] and MoS_2[7] in high accuracy.[3-5] IFF has been extensively used to examine bulk and hybrid materials containing metals, alloys, oxides, electrolyte, protein, DNA interfaces, as well as polymer composite materials. Extensions for modeling of chemical reactions were recently introduced in IFF-R to extend IFF for chemical reactions while maintaining full compatibility.[8]

Computational literature focusing on MXene modeling has been centric on quantum mechanical methods, primarily Density Functional Theory (DFT). DFT is an established *ab initio* technique that uses molecular structures as inputs and can use over one hundred different density functionals to supplement sparse experimental data. A recent review by Zhu et al.[9] summarized over 300 DFT studies since 2011 to predict mechanical, electrochemical, and physical properties of MXenes. For example, Fu et al.[10] reported in 2016 that the choice in MXene functional groups greatly impacts surface absorption energies and can benefit shear performance. A study by Kazemi et al.[11] examined these trends for a series of $Ti_{x+1}C_x$ and $Ti_{x+1}N_x$ MXenes, identifying oxygen as a favorable termination group to increase strength, along with thicker 2D layers.

While DFT studies provide significant guidance for designing novel MXenes, utilization is limited to extremely small periodic systems (often unit cells of ~1 nm dimension)[12] and the accuracy lags behind state of the art force fields (Table 1). Large-scale computational resources are needed as the presence of *d*-orbitals in transition metals ('M') in MXenes requires many basis functions and unknown or approximate dispersion terms. DFT results can vary more than an order of magnitude for the same structural inputs depending on choice of density functional, for example, due to choices in handling long-range dispersion, many adjustable parameters, and limited interpretability of deviations relative to experimental reference data.[2, 13-16] A large share of DFT studies is carried out at temperatures of 0 K, which is far removed from experimental conditions, and involves extremely limited dynamics on the order of picoseconds.

In contrast, molecular dynamics simulations with interpretable interatomic and reactive potentials are 10^6 to 10^7 times faster and often more accurate than DFT methods (comparable to CCSD(T)), allowing simulations of larger and practically more relevant systems from atoms to micrometers. Access to dynamics over microseconds also allows visualization of adsorption and assembly processes, paired with predictions of microscopic or macroscopic mechanical and thermal properties.[2]

At the same time, the accuracy of properties calculated via MD simulations remains dependent on the suitability of the potential energy function, interpretable force field parameters, and good simulation practices. It is desirable that force field-based atomistic frameworks are transferable to allow similar calculations for a diverse range of MXene compositions, surface functional groups, electrolyte, inorganic, and polymer interfaces (Table 1).

Only a small number of MD studies have been reported to-date, primarily focusing on $Ti_3C_2T_x$. For example, Plummer et al.[17] employed the ReaxFF force field to model nano-indentation of single layer MXene sheets for predicting in-plane Young's modulus. The calculated values (~455 GPa) notably deviate from experimental values (~330 GPa) measured by Lipatov et al.,[18] and the force-displacement data from nanoindentation simulations deviate approximately 20 times from experimental data (see main text). Borysuik et al. [19] calculated the elastic properties of Ti_3C_2 without surface groups (T_x) along with experimental validation. Hatam Lee et al. [20] calculated in-plane Young's moduli (~188 GPa) with the COMB3 potential and suggested that $Ti_3C_2T_x$ MXene may be significantly weaker than previously reported values. These data contradict experimental measurements and mechanical properties known for other 2D materials (e.g., clay minerals) that are more compliant than carbides.[21] Several studies also modeled the mechanical behavior of MXene-containing composites. Zhou et al. coupled an OPLSS-AA MD graphene model with a rigid-body MXene model to simulate fracture in a simplified graphene-MXene composite.[22] Other MD studies specifically parameterized models for targeted functionalities. For example, Meidani et al.[23] employed the Atomic Simulation Environment, a Python package for MD simulations, to investigate the effectiveness of different pore sizes within rigid-body MXene nanosheets towards diffusion vs. exclusion of anions for seawater desalination. Ding et al.[24] explored $Ti_3C_2T_x$ for use in gas separations using the Universal Force Field (UFF), which is known to yield unreliable or nonphysical results.[4, 25] Zhang et al.[26] simulated interactions between fully-oxygen terminated MXene layers and calculated friction coefficients, although without validation of the surface properties against experimental reference data. In summary, there has been no reliable and transferable force field for MXenes to-date, despite the importance of computationally assisted

exploration of their properties. This work introduces a robust, accurate, and interpretable model that surpasses the capabilities of prior MD models.

S1.4. Advances in This Work. The gaps closed in MD simulations of MXenes and MXene-based materials in this work include (1) mobility of all atoms as opposed to fixed atoms in prior studies, (2) inclusion of a variety of surface functional groups versus limited terminations in earlier studies, (3) thorough validation against multiple experimental properties (lattice parameters, contact angles, in-plane modulus), and verified predictions of non-fitted properties (thermal, mechanical, AFM nanoindentation, failure mechanism), (4) quantitative insights into the anisotropy of mechanical and thermal properties for different surface terminations in support of structural and thermal applications, (5) first tests of the impact of defects on thermal conductivity, (6) compatibility with CHARMM, OPLS-AA, CVFF, PCFF, and other force fields without added parameters to study interactions with solvents and organic molecules, (7) a low number of interpretable parameters and low computational expense versus high number of parameters and high computational expense in ReaxFF and COMB3 models, (8) exceptional accuracy (Table 1).

Prior to the inclusion of MXenes, IFF and IFF-R covered 100+ distinct compounds and unlimited mixtures, including chemically similar metal oxides and hydroxides, carbon nanostructures, and clay minerals.[2, 7] IFF model parameters are trained by quantitative descriptions of chemical bonding (Extended Born Model)[2, 13] supported by atomic-scale experimental data on atomic charges, internal multipoles, atomic sizes, and polarizabilities. A well-defined workflow correlates parameter refinements with validation of macroscopic properties (Figure 2f). The interpretable theory and workflow provide a consistent modeling strategy for compounds across the periodic table, enabling MXene models with diverse surface groups and transferability for inorganic-electrolyte and inorganic-organic interactions.

S2. Details of the Energy Expression and IFF/IFF-R Force Field Parameters

S2.1. Energy Expression and Parameter Assignment. IFF uses a class I potential energy expression for the MXenes (Figure S1a). This energy expression has strong foundations in quantum mechanics, full compatibility with the energy expression of other force fields (CHARMM, AMBER, OPLS-AA, CVFF, DREIDING with a 12-6 LJ potential) and maximizes speed for large scale MD simulations.[2] IFF also has full compatibility with class II potential energy functions, including PCFF, CFF, and COMPASS with a 9-6 LJ potential. Compared to the full spectrum of energy terms shown in Figure S1a, MXenes only require bonded terms for bond stretching and angle bending, as there are no torsional degrees of freedom in the carbide framework:

$$E_{pot} = \sum_{ij\ bonded} K_{r,ij}(r_{ij} - r_{0,ij})^2 + \sum_{ijk\ bonded} K_{\theta,ijk}(\theta_{ijk} - \theta_{0,ijk})^2 +$$

$$+ \frac{1}{4\pi\varepsilon_0\varepsilon_r} \sum_{\substack{ij\ nonbonded \\ (1,2\ and\ 1,3 excl)}} \frac{q_i q_j}{r_{ij}} + \sum_{\substack{ij\ nonbonded \\ (1,2\ and\ 1,3 excl)}} \varepsilon_{ij} \left[\left(\frac{r_{min,ij}}{r_{ij}} \right)^{12} - 2 \left(\frac{r_{min,ij}}{r_{ij}} \right)^{6} \right], \quad \text{(S1)}$$

$$with \quad r_{min,ij} = \sqrt{r_{min,ii} r_{min,jj}}\ and\ \varepsilon_{ij} = \sqrt{\varepsilon_{ii}\varepsilon_{jj}}$$

$$and\ the\ LJ\ potential\ sometimes\ equivalently\ shown\ as:$$

$$\varepsilon_{ij} \left[\left(\frac{r_{min,ij}}{r_{ij}} \right)^{12} - 2 \left(\frac{r_{min,ij}}{r_{ij}} \right)^{6} \right] = \frac{A}{r_{ij}^{12}} - \frac{B}{r_{ij}^{6}}$$

$$A = \varepsilon_{0,ij} r_{min,ij}^{12} \quad , \quad B = 2\varepsilon_{0,ij} r_{min,ij}^{6} \quad , \quad r_{min,ij} = \sqrt[6]{\frac{2A}{B}}.$$

The 12-6 LJ potential can use different combination rules (geometric r_{min} vs arithmetic r_{min}), and also be substituted by a 9-6 LJ potential with Waldmann-Hagler combination rules.[2]

Atomic charges were assigned based on the Extended Born model and validated values for similar titania, silica, and metal carbide chemistries in IFF.[2, 13] Initial Lennard Jones non-bond constants (r_{min}, ε), as well as bond and angle constants were taken from existing IFF parameters based on chemical similarity. Refinements of the initial parameter sets were carried out following a 3-step approach to replicate key experimental values: lattice parameters, contact angles known from experiment as a proxy for unknown cleavage energies from experiments, and vibrational spectra (Figure 2f).

In the IFF-R variant, the nonreactive energy term for the harmonic bond energy was replaced with a shifted Morse potential (Figure S1b).[8] Hereby, bond lengths and vibration constants remain the same. The representation of bond vibrations changes from the constant $K_{r,\,ij}$ in the non-reactive force field to an equivalent value of α_{ij}, and a bond dissociation energy D_{ij} is added (Table 2):

$$E_{Morse-bond} = \sum_{ij\ bonded}(D_{ij}[1 - e^{-\alpha_{ij}(r_{ij}-r_{0,ij})}]^2 - D_{ij}) \quad \text{(S2)}$$

This modification is only needed for bonds where (1) IFF uses bonded terms (as opposed to nonbonded terms only), and (2) the bonds are considered breakable. For example, when bonds are represented by nonbonded terms as in the attachment of surface functional groups (OH, F) to Ti, IFF is already reactive and requires no shifted Morse potential for these bonds. For MXenes, therefore, only the Ti-C bonds (and the O-H bonds, if desired) require switching of the harmonic bond potential (Equation (S1)) for a shifted Morse potential (Equation (S2) and Table 2). The bond dissociation energy D for Ti-C bonds is a critical quantity for simulating reactivity and fracture. The chosen value of 60 kJ/mol was obtained from the tabulated heat of formation of titanium carbide, -180 kJ/mol,[27] divided by 3 to account for a per-bond contribution (coordination number

of 6, equal to 3 Ti-C bonds per TiC formula unit). The value *D* = 60 kJ/mol was further supported by bond dissociation energies in similar chemical environments and has a ±10% error bar.[28] *D* likely changes for successive bond breaking steps at the same atom, which we do not further consider here. Further adaptation to the chemical environment can be implemented using chemical theory, data from CCSD(T) quantum calculations, and other reliable source data if necessary.

The parameters are listed in Table 2. Table S1 includes the parameters again with some further details and a complete list of the instances of all individual bonds and bond angles. Bond lengths, angles, bond stretching and angle bending constants are defined by the X-ray crystal structure data and IR/Raman spectra, and therefore not adjustable (or minimally adjustable) paremeters. Physically reasonable ranges for adjustability in other force field parameters were approximately ±0.05e in atomic charges, ±5% in LJ r_{min}, and ±10% in LJ ε values (validation range in Figure 2f).

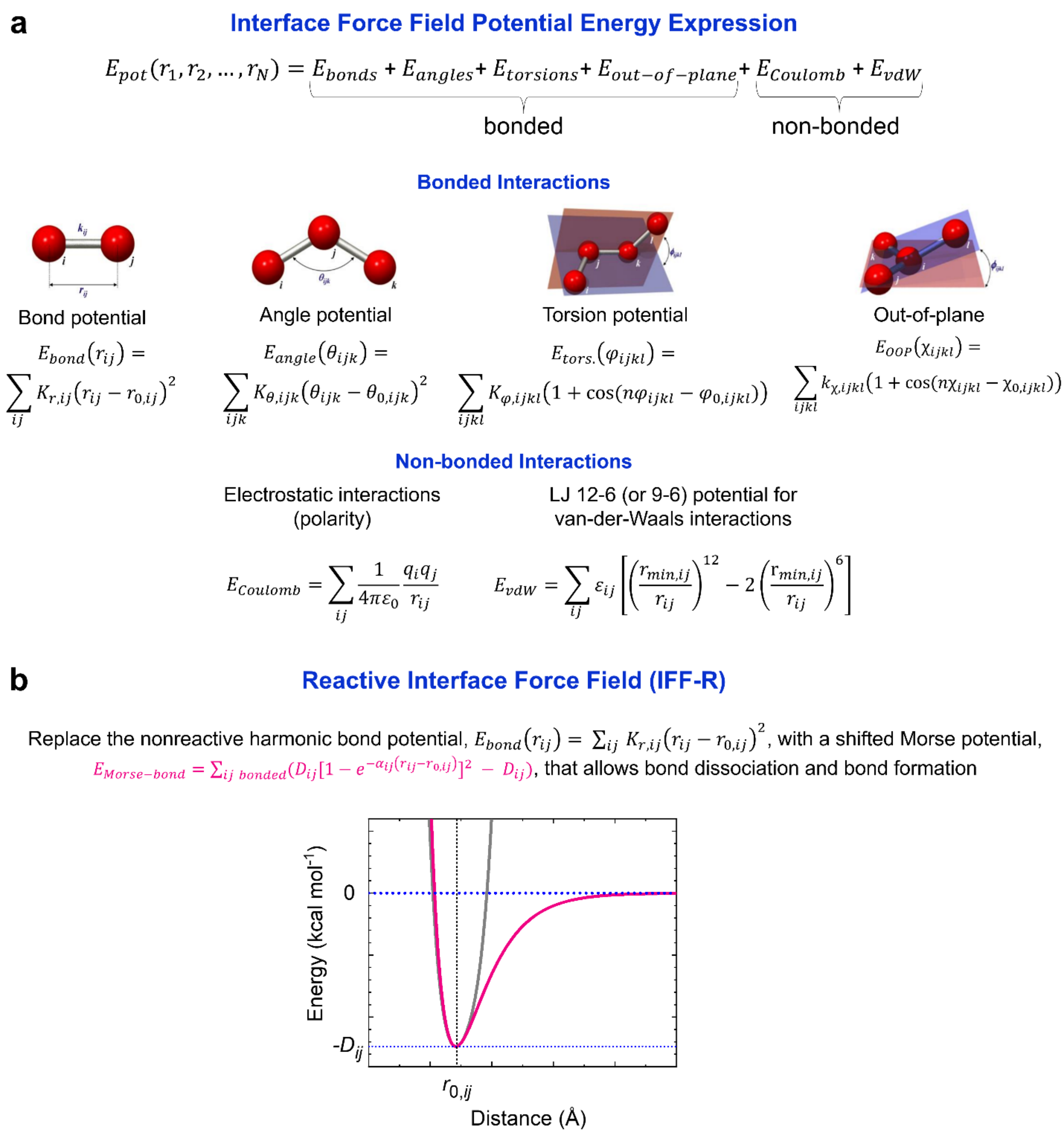

Figure S1. Energy expression of IFF and IFF-R. (a) The Interface Force Field (IFF) has been developed for simulations of compounds across the periodic table and uses the energy terms shown above. IFF is based upon interpretable parameters that accurately represent chemical bonding and reproduce structural as well as the energetic properties of included compounds under standard conditions relative to experimental data and theory. The accuracy exceeds DFT methods up to an

order of magnitude.[2, 5, 8, 13, 29] IFF is designed to be transferable and compatible with existing harmonic force fields for organic molecules and biopolymers by using the same potential energy terms, including 12-6 and 9-6 options for the Lennard-Jones potential.[2] IFF covers, for example, metals, minerals, oxides, 2D materials, common water models, solvents, gases, organic compounds, and performs exceptionally well for mixtures, solid-electrolyte interfaces, and hybrid materials. Lattice parameters and densities of pure phases usually deviate <0.5%, surface and hydration energies <5%, and mechanical properties <10% from reference data. This accuracy is about one to two orders of magnitude higher than other force fields for the same compounds.[2, 5, 13, 29] The reliability exceeds common electron density functionals[30-33] especially with regard to simulating interfaces.[7, 29] IFF is compatible with biomolecular force fields such as CHARMM,[34] AMBER,[35] OPLS-AA,[36] as well as PCFF[37, 38], and COMPASS,[39] extending access to protein, DNA, lipid, and carbohydrate based multifunctional materials.[8] (b) The reactive INTERFACE force field (IFF-R) enables reactive simulation with minimally modified parameters.[8] The energy term for harmonic bond stretching is substituted by a shifted Morse potential with zero energy at large bond distance. The reactive bonds retain the equilibrium bond length as in the harmonic representation, obtain a different representation for the force constants (α_{ij} instead of $K_{r,\,ij}$), and an added bond dissociation energies D_{ij} from experiment or high-level quantum mechanical calculations. All other parameters in the force field remain the same and do not affect the results, as demonstrated in ref. [8]. The shifted Morse potential is implemented in LAMMPS and can be added as a custom energy term in other MD programs (NAMD, GROMACS, OpenMM).

Table S1. Full $Ti_3C_2T_x$ MXene IFF model parameters, including nonbond parameters and bonded parameters for all distinct bonds and angles. The IFF and IFF-R parameters are compatible with CVFF,[40] OPLS-AA,[36] CHARMM,[34] AMBER,[35] DREIDING[41] (12-6 LJ), as well as PCFF,[37, 38] CFF,[42] and COMPASS (9-6 LJ).[43, 44] Differences in use with these energy expression only include the LJ parameters as shown. Bonds and bond angles reflect the MXene topology according to X-ray data. Two atom types of Ti and four atom types of C were used to represent the Ti-C framework (Figure S2). We classified one bond type with 8 specific instances and five angle types with 28 specific instances. Multiple instances of each type are necessary to define the MXene topology.

**I. Nonbond**	**Atomic charge *q* (e)**	**r_{min} (Å)**			**Well depth ε (kcal mol^{-1})**	
		12-6 LJ: IFF-CVFF, OPLS-AA (geometric $r_{min,\ ij}$)	12-6 LJ: IFF-CHARMM, AMBER (arithmetic $r_{min,\ ij}$)	9-6-LJ: IFF-PCFF, CFF, COMPASS	12-6 LJ (all)	9-6 LJ (all)
Ti_{int} (ti1)	+0.35	**2.25**	**2.16**	2.30	0.20	0.16
Ti_{ext} (ti2)	+0.675	**2.15**	**2.06**	2.20	0.30	0.24
C (cx1, cx2, cx3, cx4)	-0.35	3.80	3.80	3.90	0.07	0.06
O (omx)	-0.788	3.45	3.45	3.60	0.19	0.16
H (hoy)	+0.288	1.085[a]	1.085[a]	1.098	0.015[a]	0.013
F (fx)	-0.5	3.26	3.26	3.40	0.17	0.14

[a] Can also be set to zero, or 0.001, with negligible loss in accuracy.

**II. Bonds**	$r_{0,ij}$ **(Å)**	$K_{r,\,ij}$ **(kcal mol^{-1} Å^{-2})**
Ti – C<br>(ti1-cx1, ti1-cx2, ti1-cx3, ti1-cx4, ti2-cx1, ti2-cx2, ti2-cx3, ti2-cx4)	2.157	285
Same bond lists for reactive bonds in IFF-R	2.157	α = 2.0 Å^{-1}, D = 60 kJ mol^{-1}

**III. Angles[a]**	$\theta_{0,ijk}$ **(°)**	$K_{\theta,\,ijk}$ **(kcal mol^{-1} rad^{-2})**
ti1 – cx* – ti2<br>(ti1-cx1-ti2, ti1-cx2-ti2, ti1-cx3-ti2, ti1-cx4-ti2)	Varies	0[b]
ti2 – cx* – ti2<br>(ti2-cx1-ti2, ti2-cx2-ti2, ti2-cx3-ti2, ti2-cx4-ti2)	93.5	67
ti1 – cx* – ti1<br>(ti1-cx1-ti1, ti1-cx2-ti1, ti1-cx3-ti1, ti1-cx4-ti1)	91.6	67
cx* – ti1 – cx**<br>(cx1-ti1-cx2, cx1-ti1-cx3, cx1-ti1-cx4, cx2-ti1-cx3, cx2-ti1-cx4, cx3-ti1-cx4)	90.0	40
cx* – ti2 – cx*	90.0	40

(cx1-ti2-cx1, cx1-ti2-cx2, cx1-ti2-cx3, cx1-ti2-cx4, cx2-ti2-cx2, cx2-ti2-cx3, cx2-ti2-cx4, cx3-ti2-cx3, cx3-ti2-cx4, cx4-ti2-cx4)		
cx1 – ti1 – cx1	180.0	67
cx2 – ti1 – cx2	180.0	67
cx3 – ti1 – cx3	180.0	67
cx4 – ti1 – cx4	180.0	67

[a] Angles were optimized to replicate equilibrium atomic positions in X-ray data during MD simulations under standard conditions. Cx* denotes any carbon type, Cx** denotes a different carbon type of any type (Cx* cannot equal Cx**). Using the atom types (two for Ti, four for C), a total of 28 distinguishable angles were identified. [b] These angles made no contribution to the potential energy: (i) by setting certain angle vibration constants zero, we avoid many superimposed angle constraints that could artificially increase stiffness, (ii) this concept, as used earlier in IFF for MoS_2,[7] can circumvent the definition of additional force field types, especially when some angles could assume multiple values (e.g., both 180 and 90 degrees) and make no essential contribution to the potential energy.

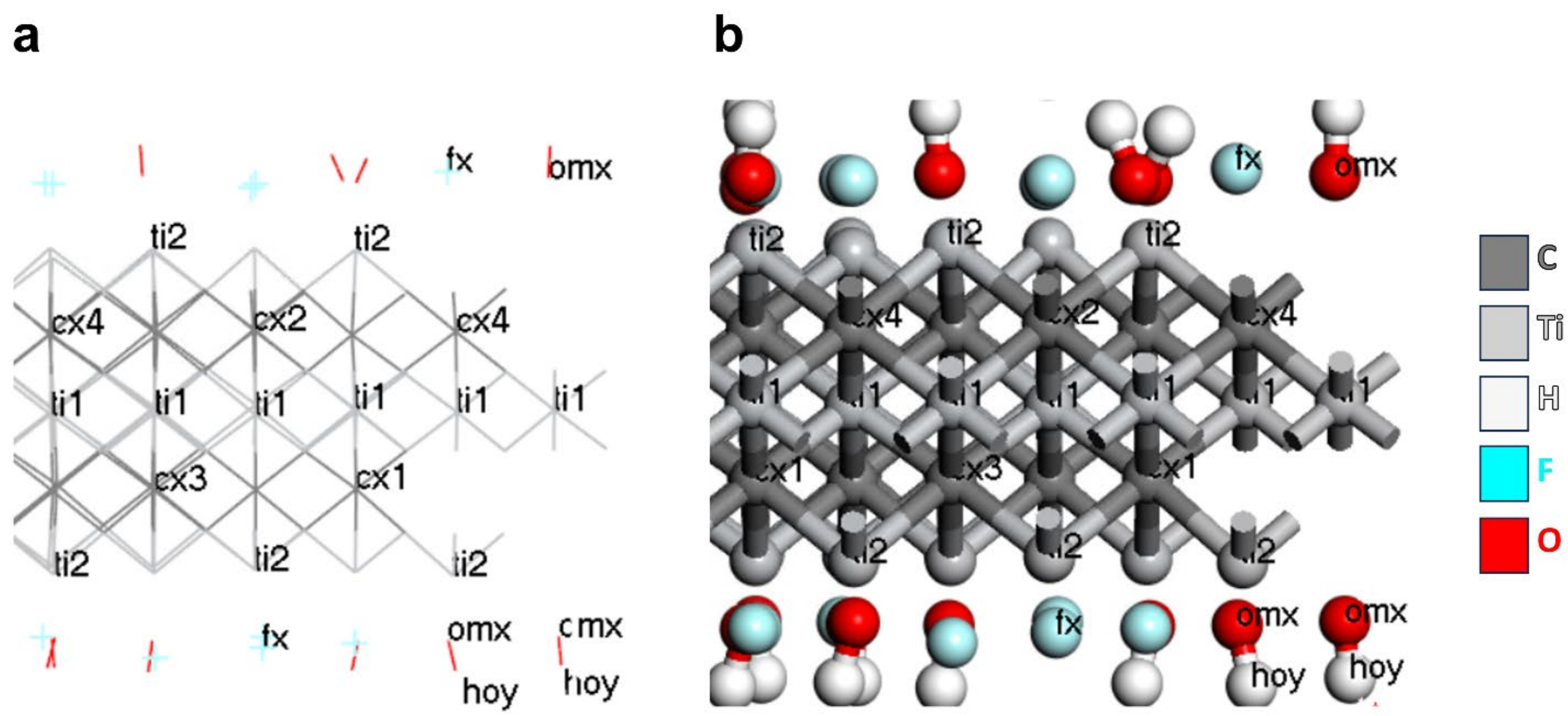

Figure S2. Structural fragment of $Ti_3C_2T_x$, indicating the atom types assigned in IFF and IFF-R. The topology aids in understanding the definition of unique bonds and angles in Table S1. (a) Line view. Note the repeating pattern of four carbon atom types in the lattice and the difference between interior (ti1) versus exterior titanium atoms (ti2). (b) The same structural fragment in ball-and-stick view.

S2.2. Representation of Surface Terminal Groups. The protocol was augmented to incorporate different surface terminal groups, -F and -OH, including mixed surface chemistries. The OH^- and F^- (partially covalent) ions can be easily substituted by other anions and ethers such as O-R without requiring new IFF parameters. The bonds of these surface terminal groups *T* to Ti in $Ti_3C_2T_x$ are substantially weaker when compared to interior Ti-C MXene bonds and were modeled as non-bonded Lennard-Jones interactions that superimpose with the bonded representation in the Ti-C plane (Figure 2 and Figure S2). This nonbonded representation of surface groups is consistent with at least 50% ionic character of these bonds and offers three key

advantages. First, it positions the surface groups naturally in the center of 3 Ti atoms for a 4-electron-4-center bond (2 valence electrons from Ti plus 2 valence electrons from OH^- of F^-). Otherwise, numerous bond and angle terms would be required. Second, the nonbonded representation greatly simplifies the process of substituting surface functional groups for others and creating mixed surface terminations. Third, the nonbonded setting enables rapid, modular parameter changes during training and optimization, for example, when modifying parameters for a different MXene composition to align with the experimental geometry.

For extensions to new surface terminal functional groups, it is only required to examine the balance of atomic charges of the anchoring atom in the new surface functional group and adjust its LJ parameters to represent the bonding geometry. It is not necessary to add bonded parameters for the interaction of new T groups with Ti, i.e., no equilibrium bond lengths, bond angles, and force constants as shown in Equation (S1) need to be specified.

Furthermore, we analyzed the sensitivity of surface -OH groups to protonation and deprotonation equilibria as a function of solution pH values in analogy to previously studied Si-OH group on silica, P-OH groups in phosphate species, and aluminate/Al-OH species in cement minerals (Figure 4a, b). $Ti_3(OH)_2^+$ groups at pH values <6 were represented together with a chloride anion for charge neutrality, and involved modified charges of the OH_2 groups only (-0.4e on O and +0.45e on H, leaving a net +0.5e charge on the Ti surface and on the protonated group). Charge distributions that shift the total charge of +1.0e onto the 3 surrounding Ti atoms (+0.675e --> +0.842e for each) and create a neutral bound water molecule OH_2 (O: -0.82e and H: +0.41e) can also be explored.

S2.3. Workflow for Parameter Training, Validation, and Property Prediction. The workflow from training data to validation and predictions is shown in Figure 2f. We do not directly use QM, especially not DFT, to assign or validate parameters in IFF due to large associated uncertainties and lack of interpretability. We obtain atomic charges and initial LJ parameters from experimental atomic-scale data, e.g., X-ray electron deformation densities, dipole moments, the extended Born model, atomic/crystallographic radii and polarizabilities (as described in prior publications, refs. [2, 13]). We use experimentally reported bond dissociation energies in reactive Morse bonds (ref. [8]). These training data focus on understanding chemical bonding across the periodic table, are based on reproducible experiments, have low error margins and low likelihood for changes over time.

The IFF approach yields more reliable atomic charges (±0.1e or <±10%) for particle-based simulations than QM methods as it removes dependence on basis sets, density-functional, and partitioning methods.[2, 13, 29] The QM methods are more suited for field-based representations. In summary, the training set includes initial parameters that reflect the above atomic-scale properties and narrow physical ranges for the FF parameters (<10% in Ti, C and O charge, <10 % in LJ params for O, C and F, <2% in bond lengths and angles, <5% in vibration constants).

Properties for validation, or refinement of training (Figure 2f), include lattice parameters, key vibration frequencies compared to Raman spectroscopy (limited due to lack of explicit electron density in IFF), contact angle with water (since cleavage energy not available), and in-plane Young's modulus relative to experiment.

The prediction set and further validation (non-fitted properties) constitute most of this contribution. We computed cleavage energies for different surface terminations, contact angles with another liquid (CH_2I_2) and for other surface terminations (OH, F) relative to experimental

data (Figure 3), aqueous interface and organic binding (dopamine) relative to experiment (Figure 4), directional mechanical properties including nanoindentation and failure relative to experiments in (Figures 5, 6 and 7), thermal conductivity and role of defects relative to experiment (Figure 8).

While there is limited experimental data to validate predictions, we examine a broad set of properties up to the large nanometer scale. Such broad comparisons have not been feasible with other computational techniques. Our work is not designed as a fitting exercise from training set to validation set using residuals or other "empirical" metrics. At every level (Figure 2f), we focus on interpretability and critical reasoning of the parameters. This protocol facilitates reliable performance outside the training/validation range and positions the "parameters" (Table 2) as descriptors for a wide range of physical-chemical properties of MXenes and related multiphase materials. Our benchmark is to "be the best interpretable model possible and allow reproducible property exploration in high quality" using a QM-consistent energy function rather than to "minimize a customized residual". The validated parameters were then applied across different energy expressions, as discussed below.

S2.4. Use of IFF and IFF-R Parameters with Multiple Energy Expressions. The atomic charges and the bonded parameters (including reactive Morse bonds) are identical for all energy expressions. Small differences arise in the nonbonded parameters as shown in Table 2 and Table S1. When incorporating these minor changes, the lattice parameters, densities, and cleavage energies are identical within the errors bars for all energy expressions used.

Very few modifications are required when switching between IFF-CVFF, OPLS-AA and IFF-CHARMM, AMBER (see bold highlights in Table S1). The change in combination rules for r_{min} slightly lowers the optimum values for Ti in CHARMM and AMBER. 9-6 LJ potentials use a different mathematical form and therefore required slightly more modifications (Table S1).

Even if the IFF-CVFF, OPLS-AA nonbond parameters are used in PCFF without change, the average deviation in lattice parameters remains <0.7% (although 2% compression in the *z* direction) and the cleavage energy increases by +13%. Using the appropriate nonbond parameters as indicated in Table S1 marginalizes the differences below the default uncertainties, i.e., <0.5% for all lattice parameters and <5% difference in cleavage energy relative to experiment or each other. Some tradeoffs in some mechanical properties may be observed using the 9-6 LJ potentials (the performance is equal to 12-6 LJ for bonded models and typically lower in nonbonded models due to weaker repulsion).

Different scaling of nonbond interactions between 1, 4 bonded atoms in 12-6 LJ potentials has minor or negligible effect for the MXene models. 1, 4 bonded interactions do not occur near nonbonded surface groups and are only found within the internal TiC bonded framework. The structure and energy differences in the heavily bonded TiC framework are almost exclusively maintained by bonded and angle terms, and the impact of atomic charges on 1,4 bonded atoms is rather low due to the small magnitude (typically ±0.35e). Also, the impact of vdW/LJ interactions on 1,4 bonded atoms is minor in relation to the bonded terms. Therefore, 100% scaling of both Coulomb and LJ interactions between 1, 4 bonded atoms in the internal TiC lattice in CVFF and CHARMM, versus 83.33% scaling of Coulomb interactions and 50% scaling of LJ interactions in AMBER, versus 50% scaling of both Coulomb and LJ interactions in OPLS-AA has only marginal effects on lattice parameters and surface interactions. The effects of different scaling of nonbond interactions between 1, 4 bonded atoms thus stay within the reported uncertainties relative to experimental data.

The potentials with 9-6 LJ parameters (PCFF, CFF, and COMPASS) use 100% scaling of both Coulomb and LJ interactions between 1, 4 bonded atoms and have slightly modified LJ parameters

by default. The nonbond equilibrium distances r_{min} in the 9-6 LJ potential are slightly increased relative to the 12-6 LJ potentials, and the well depths ε are slightly reduced (Table S1) due to weaker repulsive interactions and a larger area under the dispersive energy portion of the potential curve.[5] The ratios of LJ parameters in 9-6 versus 12-6 form are about the same here as in IFF potentials for other chemistries (e.g., silica, aluminates, metals, clay minerals, MoS2, oxides, hydroxides, and gases).[4, 5, 7, 25, 45-48]

S3. Comparison of Computed Vibration Spectra with Experimental Spectra

Infrared and Raman spectra capture vibrational responses that correspond to the Ti-C bond and angle parameters, as well as the superposition with nonbonded parameters. To compute the spectra from MD simulations, the velocity autocorrelation function (VACF) was employed using frames spaced 1 fs to 3 fs apart over a short equilibrium trajectory (~5 ps), followed by a power spectral analysis of the VACF via a Fourier transform. Computed spectra do not include selection rules and superimpose all vibrational modes across the wavenumber spectrum. Only some of these modes may be activated in experiments.

Unlike the data for elastic moduli, experimental reports of Raman spectra of the $Ti_3C_2T_x$ MXene agree with each other.[49-54] The MXene has well-defined peaks, including the primary, high frequency Ti-C bond vibrations at ~1200 cm^{-1} [55] (peak 7 in Figure S3a, b) that were matched quantitatively between simulations and experiments. The peak at ~1200 cm^{-1} was determined by the Ti-C harmonic bond stretching constant K_r with a value of 285 kcal mol^{-1} $Å^{-2}$ (Table 2 and Table S1, Figure S2), and this value of K_r also produced the experimental value for the in-plane elastic modulus. Using the angle bending constants, other peaks at lower wave numbers are

approximately reproduced within a frequency range of ±50 cm^{-1}. These include C-Ti-C and Ti-C-Ti angle vibrations as well as modes involving the surface functional groups TiOH and TiF involved in (O, F)-Ti-C angles. The latter modes involving the surface functional groups are represented via electrostatic and nonbonded Lennard Jones interactions and latter interactions simultaneously influence contact angles (and surface energy).

Overall, for a model that simplifies the electronic structure into atomic charges and LJ parameters, the agreement of the vibrational signatures in simulations and experiments is very good. A reasonable match in wavenumbers has also been achievable for other 2D materials, whereas a match in relative intensities remains challenging due to the lack of representation of the full electronic structure.

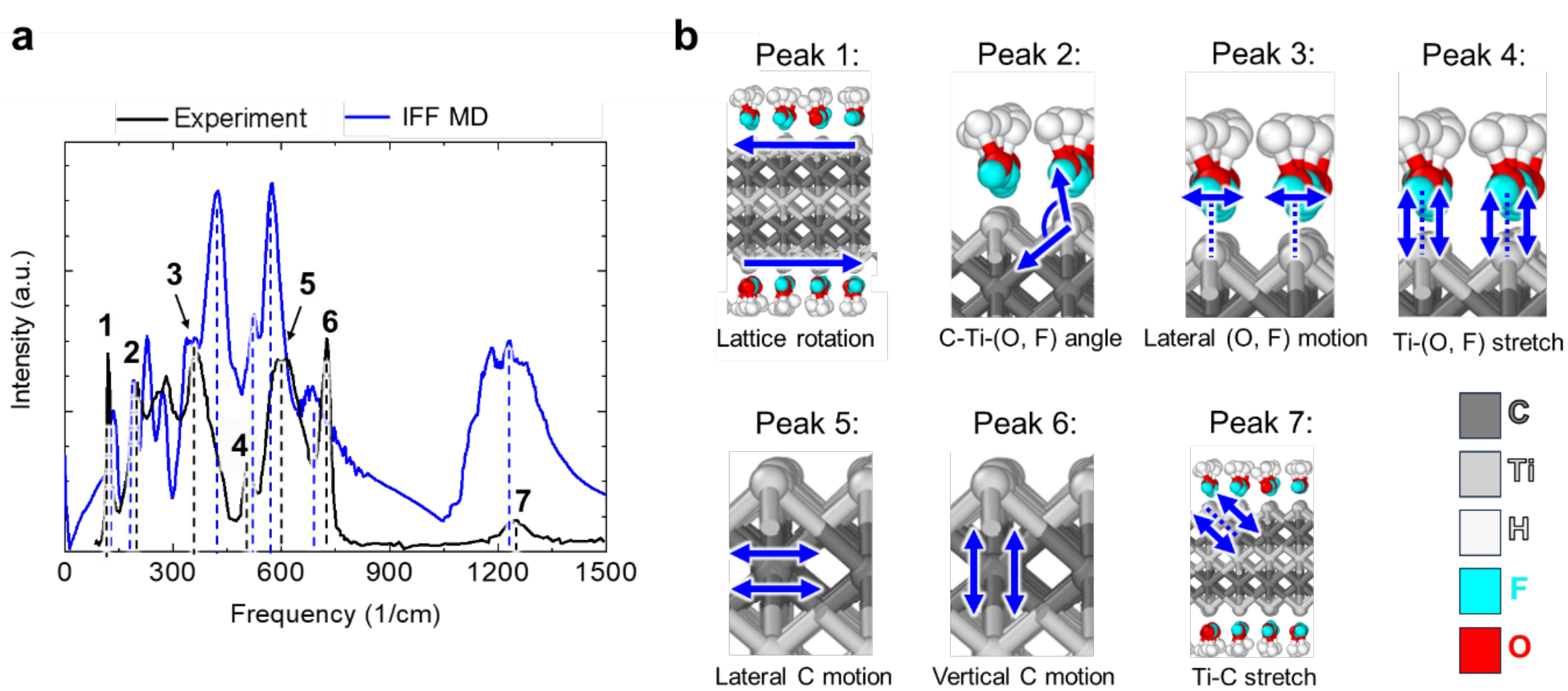

Figure S3. Raman spectra computed by MD simulation with IFF (or IFF-R) versus experimental data. (a) Superposition of simulated and experimental Raman spectra of $Ti_3C_2T_x$ MXene.[49-54] (b) We identified and explain 7 peaks whose vibrational origin has been described in previous

works.[49-54] The IFF/IFF-R models, including the Ti-C harmonic bond and angle stiffness parameters, as well as the nonbonded parameters for all atoms, show reasonable agreement, especially for the high energy peak 7 slightly above 1200 cm^{-1}. Peak alignment within 50 cm^{-1} was achieved for the seven primary peaks, with an emphasis on a close match for the peak due to Ti-C bond stretch (peak 7).

S4. Additional Discussion of Contact Angles and Surface Energies

Contact angles were used for validation of surface properties at common surface coverage as a proxy for experimentally yet unknown cleavage energies. Subsequently, we predicted contact angles of water and DIM for the full range of surface termination from 100% OH to 100% F.

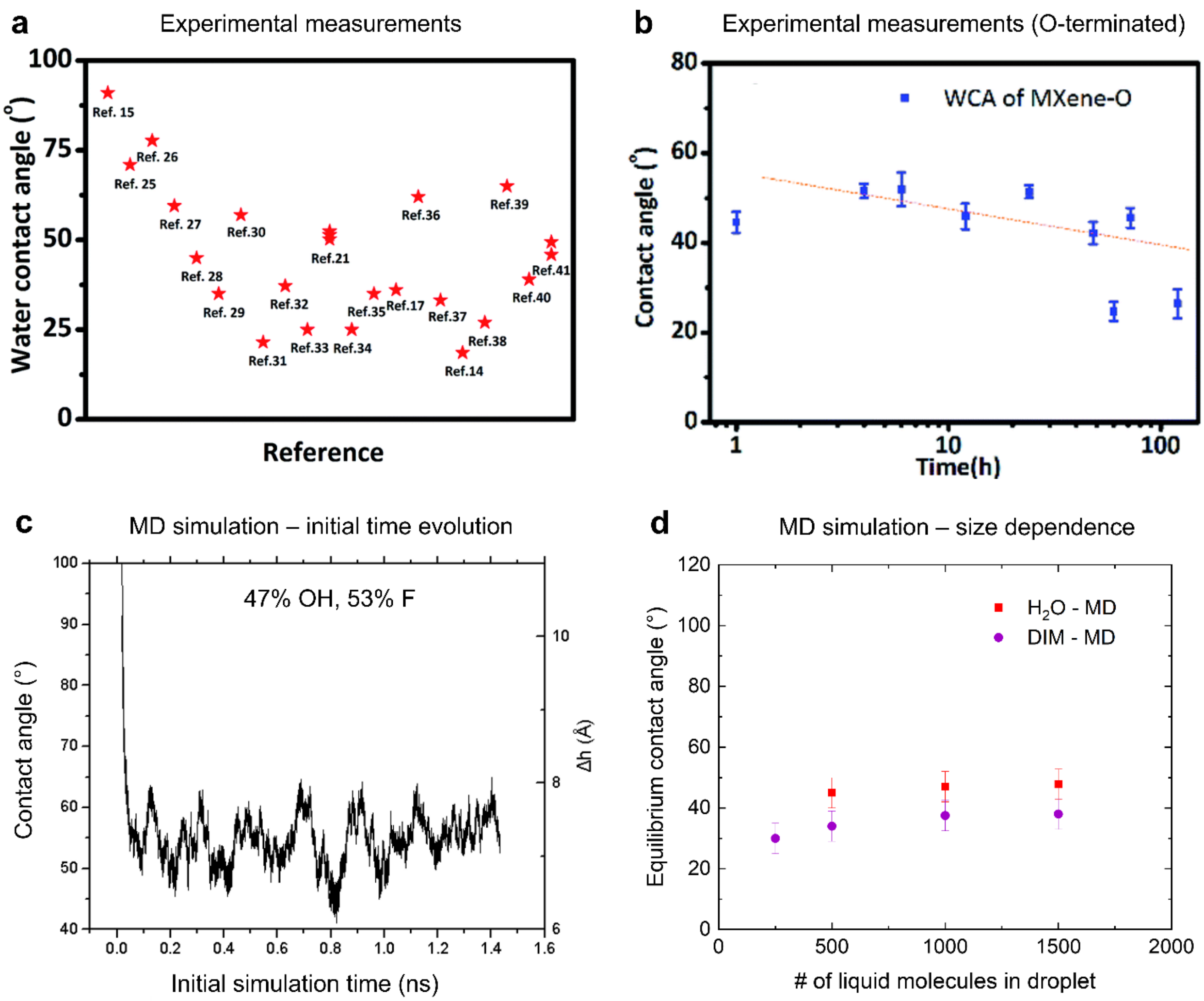

Figure S4. Contact angles of water on MXene surfaces in experiments and sample fluctuations over time in experiments and simulation. (a) Range of water contact angles reported in experiments for $Ti_3C_2T_x$, including different surface groups, defects, and sample types (reproduced with permission from Figure 1 in Zhou et al. [56]) The contact angles depend on these often not very well characterized conditions in experiments and range from ~15° to ~90°. (b) The contact angle can also decrease over time due to surface reactions and an associated increase in oxygen or OH content (part of Figure 2 from the same study by Zhou et al. [56]). The insights from these studies suggest a reasonable tolerance of ±5° for experimental benchmarks in comparison to our MD studies. (c) Example of the time evolution of the contact angle of a water droplet in MD simulations

(1000 molecules) on the MXene surface with mixed surface termination. Contact angles were calculated as averages in equilibrium (e.g., after 0.2 ns). Δh on the right vertical axis refers to the height of the droplet on the surface, which correlates with the contact angles via the equations shown in the methods section.[57] Uncertainties in computed contact angles are reported as differences in block averages, e.g., between time blocks of 1-2 ns, 2-3 ns, and 3-4 ns. (d) Dependence of the equilibrium contact angle in MD simulations (5 ns) on the droplet size. Beyond 1000 molecules, a steady value was obtained and line tension effects become negligible. The surface composition was the same as in (c).

The surface energy is a key property that determines how MXene surfaces interact with their environments, for example, for the adsorption of solvents, analytes and reactants, as well as shear stability in composites. The most straightforward definition is via layer cleavage of the pristine structure (e.g., the (0001) plane). Such cleavage is reproducible in experiments and independent on additional components like solvents. MXenes are not prone to major surface reconstruction and (instant) chemical reactions upon cleavage that would alter the surface properties. Limitations include residual solvent, impurities, and the pH-sensitivity of OH groups when present.

Older theory of surfaces relates surface energies to the contact angle of probe liquids rather than to cleavage energies. This is a useful approach in the rare case that the surfaces do not reconstruct or chemically react upon cleavage. MXene surfaces in water, for example, are pH sensitive which would mean the surface energy becomes a function of protonation/deprotonation equilibria the pH value (Figure 4a, b). Only at neutral pH values of ~6, the MXene surface chemistry in water and its surface energy would be the same as for pristine MXene in vacuum.

Owens-Wendt-Rabel & Kaelble (OWRK) theory[58] further relates the surface energy to the static contact angle (θ) of a droplet of probe liquid, where γ_s is the surface energy, γ_l is the surface tension of a liquid droplet, and the superscripts P and D refer to polar and dispersive components, respectively:

$$\gamma_s = \gamma_{sl} + \gamma_l \cos\theta \tag{S3}$$

$$\gamma_{sl} = \gamma_s + \gamma_l - 2\sqrt{\gamma_s^D \gamma_l^D} - 2\sqrt{\gamma_s^P \gamma_l^P} \tag{S4}$$

$$\sqrt{\gamma_s^D \gamma_l^D} - \sqrt{\gamma_s^P \gamma_l^P} = 0.5\gamma_l(1 + \cos\theta). \tag{S5}$$

Hereby, Equation (S3) is the Young's equation, and Equations (S4) and (S5) are the breakdowns of the surface energies (or surface tensions) into dispersive and polar components. The components, if known, could add to the validation of the IFF model as they can be equated to contributions from LJ interactions and atomic charges. These contributions directly represent dispersive van-der-Waals forces and polar electrostatic interactions, respectively.[13, 25] At this time, however, such analysis for MXenes is unlikely to yield additional insight due to lack of rigorous experimental data at controlled pH values and typical uncertainties of ±5°.

S5. Binding of Dopamine

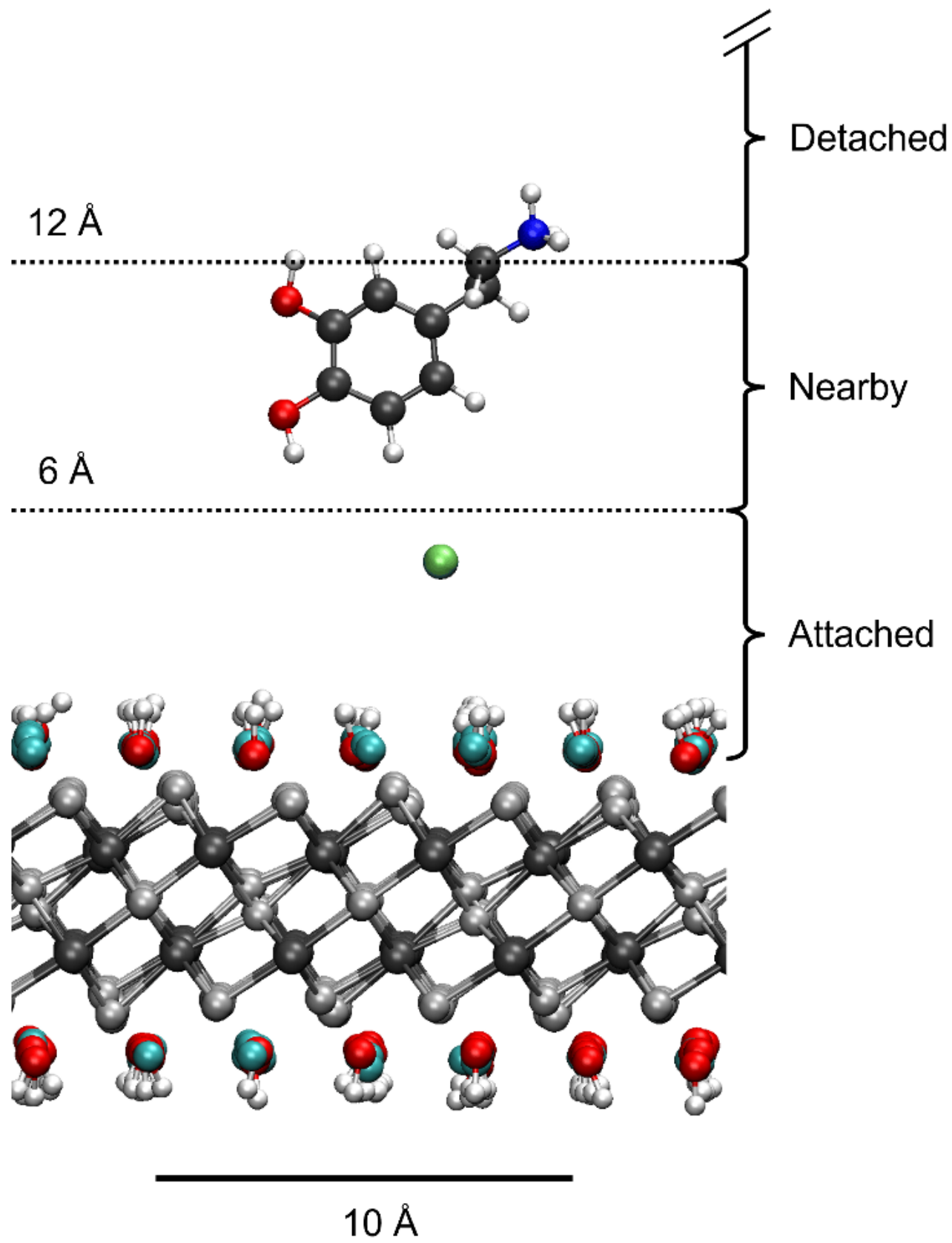

Figure S5. Definition of attached, nearby, and detached binding regimes or dopamine via distance thresholds. The center of mass of dopamine was used to measure the vertical distance (z) from the superficial layer of (O, F) atoms.

MD simulations elucidated the binding mechanism of organic molecules on MXene surfaces under chemically realistic electrolyte conditions in 3D atomic and time resolution. Sensitivity checks using nonprotonated surfaces at pH 6 instead of slightly protonated surfaces at pH 5 showed no significant differences. However, differences of 2 pH units from a realistic representation introduce notable changes in binding, and 3 pH units difference induce major shifts in mechanisms.

The observations of pH-dependent assembly on MXenes are similar to earlier studies of pH-dependent assembly on silica surfaces. Silica surfaces have a PZC of ~3 and were characterized earlier in IFF MD simulations and experiments.[46, 59] An in-depth investigation of MXene surface chemistries and organic adsorption as a function of pH value would be an interesting follow-up study along with experimental validation.

In comparison, DFT and DFT/ML methods have difficulties incorporating the influence of pH and electrolyte composition on the properties of MXene-based (and other) materials. IFF MD models overcome methodological challenges such as interpretability and speed by being simpler, faster, and grounded in experimental data.

S6. Elastic Properties

For typical surface terminations (OH, F, OR, O) that do not interconnect layers via chemical bonds, the in-plane stiffness and strength is primarily a function of the Ti-C bonded framework. Therefore, the accurate representation of related bonds and angles was critical in the model. Experimental reference data from prior studies indicate quite some uncertainty or dependence on the measurement conditions (Table S2).[18, 55, 60, 61] Measurements are especially uncertain with respect to the out-of-plane stiffness. DFT calculations exhibit a larger scatter than experiment, as expected from the underlying numerous empirical approximations (Table S2).[10, 11, 62] Average data are consistent with in-plane moduli in experimental measurements, and no data have been reported for out-of-plane moduli. Overall, the variance in experimental data can be attributed to number of MXene layers and related defects, e.g., larger thickness increases the likelihood for defects and decreased stiffness.[10, 11, 18, 61, 63] Therefore, elastic moduli may be best calculated from a high-purity

monolayer of a $Ti_3C_2T_x$ MXene via out-of-plane nano-indentation using Atomic Force Microscopy (AFM) and amount to an in-plane modulus of 330 GPa.[18] A thicker 50-layer indentation experiment, in contrast, found a 70% lower modulus of 100 GPa.[61] We utilized the 330 GPa value for in-plane tensile modulus from nano-indentation[18] as a reference due to high purity in chemical composition. This value was also backed up by reproducing the correct Raman spectra with the same bonded parameters (Figure S3).

Table S2. Data from experiments, DFT simulations, and IFF predictions for elastic moduli, *E*, of $Ti_3C_2T_x$ MXenes. High variations occur in experiments depending on methodology, surface group (if known), sample thickness, and loading direction. DFT results show a wider range than experiments. MD simulations with IFF/IFF-R reproduce in-plane moduli well and predict the to-date uncertain out-of-plane moduli.

	**Source**	**Surface groups**	**Thickness (nm)**	**Load direction**	**Elastic modulus, *E* (GPa)**
**Expt.**	Lipatov (2020)[18]	OH, F, O	Monolayer	Indentation (in-plane)	330
	Li (2020)[55, 60]	OH, F, O	5 µm	In-plane	285
	Firestein (2020)[61]	OH, F, O	40 nm	Out-of-plane	80-100
	Li (2020)[55]	OH, F, O	5 µm	Out-of-plane	268
	**Overall range (expt)**	OH, F, O	-	In-plane	285-330
		OH, F, O	-	Out-of-plane	80-285

DFT	Fu (2016)[10]	F	Monolayer	In-plane	295
	Fu (2016)[10]	OH	Monolayer	In-plane	256
	Kazemi (2019)[11]	F	Monolayer	In-plane	302
	Kazemi (2019)[11]	OH	Monolayer	In-plane	316
	Guo (2015)[62] (PBE)	O	Monolayer	In-plane	485
	**Overall range (DFT)**	OH, F, O	-	In-of-plane	256-485
		OH, F, O	-	Out-of-plane	NA
**MD**	**This Study – MD**	OH, F	Bilayer	In-plane	320 ±5
		50% OH, 50% F	Bilayer	Out-of-plane	37
		OH	Bilayer	Out-of-plane	67
		F	Bilayer	Out-of-plane	49

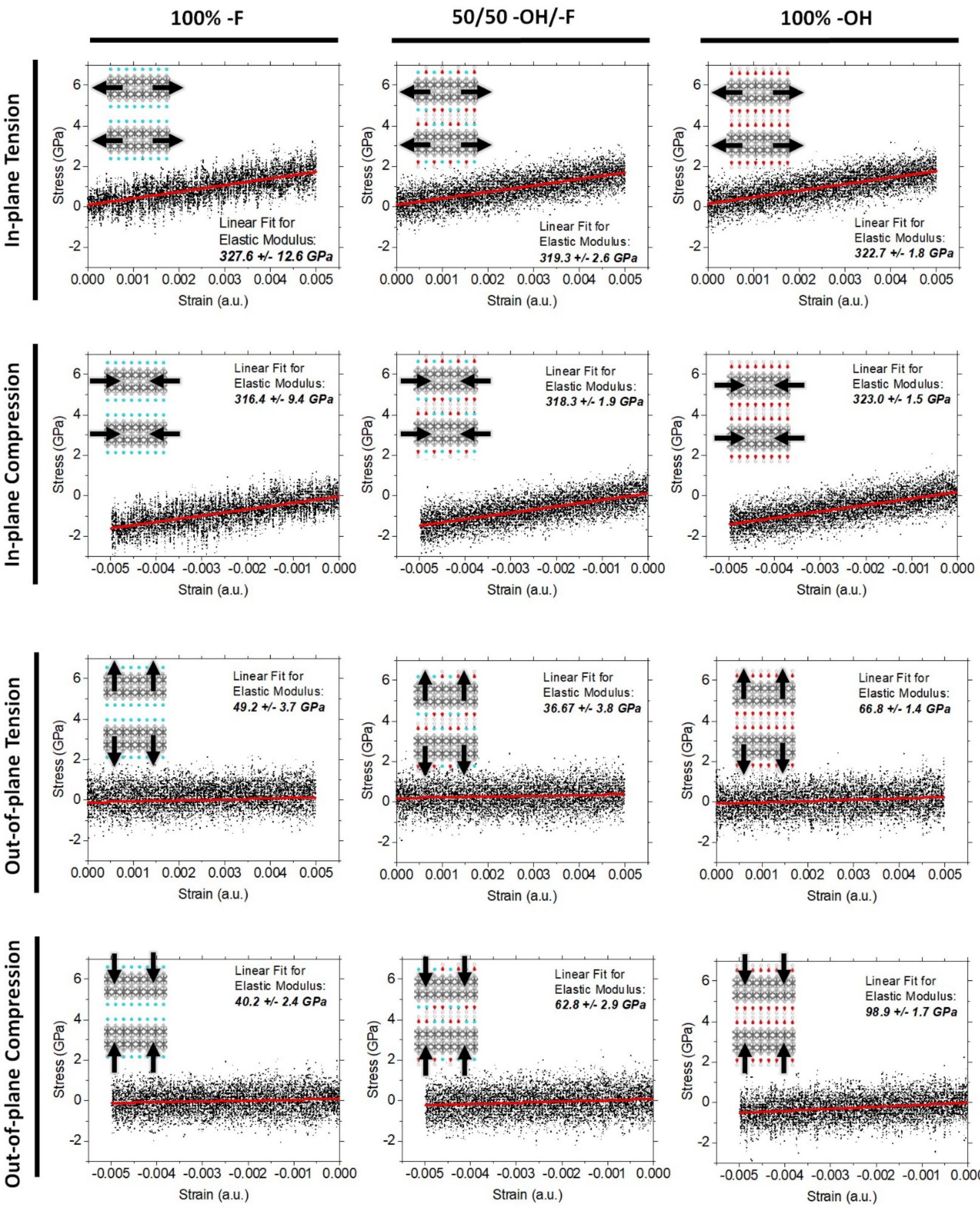

Figure S6. Extended data for the mechanical property prediction of MXenes. Stress-strain curves and elastic moduli for two load directions (tension and compression) in bilayer $Ti_3C_2T_x$ MXene models with 100% -F surface termination, a 50/50 hydroxyl(-OH)/fluorine(-F) mixed surface

termination, as well as 100% -OH surface termination. In-plane moduli were largely unaffected by the different surface groups. However, significant out-of-plane stiffening of elastic moduli was found when –OH groups replace –F on the MXene surface, especially upon compression. In case of tension, the mixed OH, F surface had a lower modulus than either of the 100% F or 100% OH terminated surface, pointing to a loss in cohesion upon contact of OH groups with F groups on opposite sides of the surface.

S7. Shear Properties

Complementing the discussion in the main text, we verified the independence of the shear stress in the *y* direction from the shear strain and shear stress in the *x* direction (Figure S7). Shear stresses in the *y* direction only emerge after more than 5% shear strain was applied in the *x* direction. The coupling occurs due to changes in surface topography after shear failure.

The computed shear properties are sensitive to volume changes in the *z* direction. When keeping the volume constant and not allowing expansion in the *z* direction upon shear under NVT conditions (Figure 6c), the resistance and shear strength is higher than when keeping the pressure constant and allowing temporary expansion in the z direction upon shear under NPT conditions (Figure 6d). Once experimental measurements become available, the values are likely to fall in the reported range, depending on whether the volume or the pressure is kept constant.

Prior studies have examined friction coefficients between MXene layers using DFT and MD simulations with ReaxFF, reporting values of 0.24 to 0.25 for $Ti_3C_2O_2$, as well as lower friction coefficients of 0.10 for OH-terminated MXene and 0.14 for O-CH_3 terminated MXene.[26]

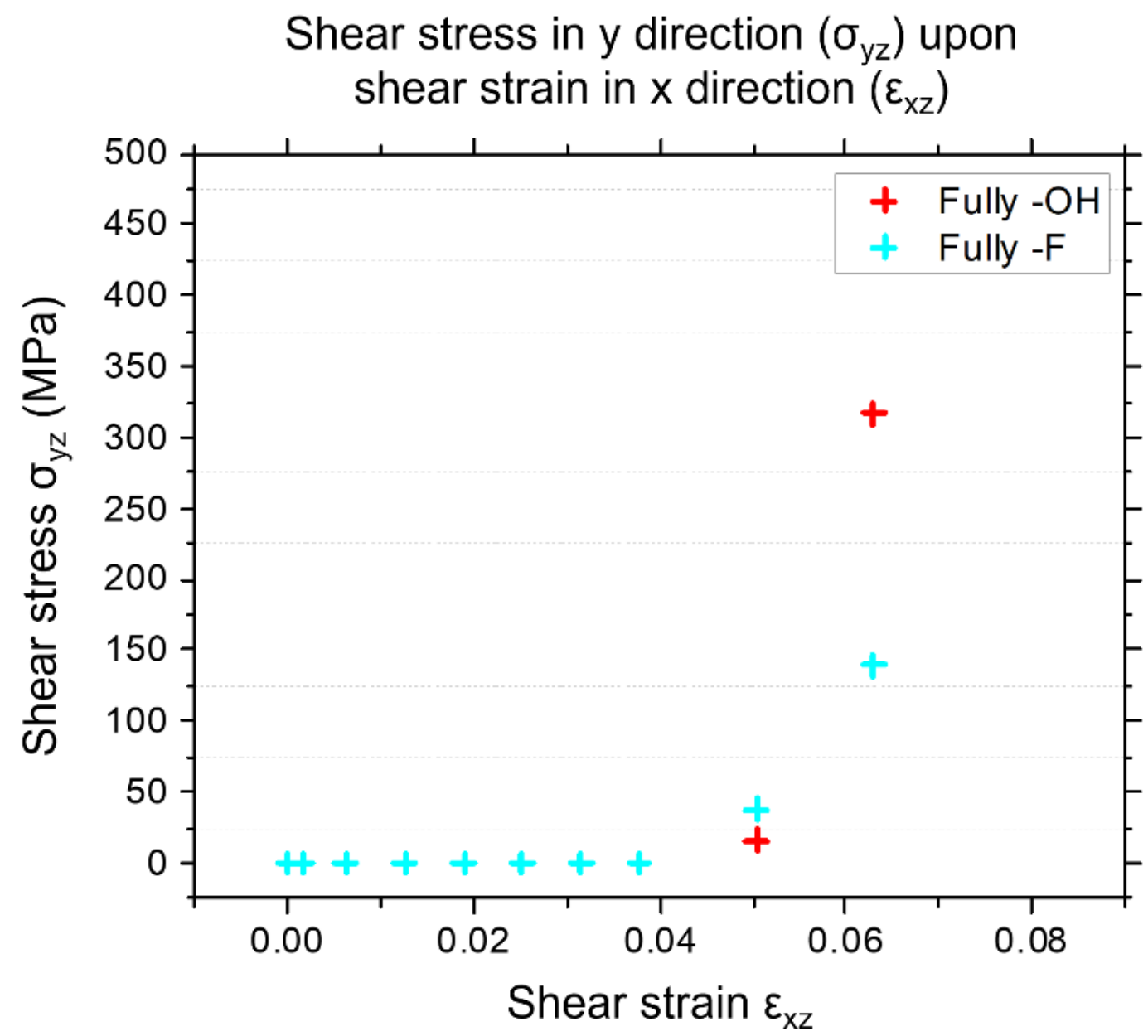

Figure S7. Independence of shear stress in the *y* direction from shear strain and shear stress in the *x* direction during the calculation of shear moduli (Figure 6c). The shear stress in the *y* direction remained at zero until the shear strain in the *x* direction exceeded 0.04 to 0.05 (~1 Å). This level of shear strain is also the onset of static shear failure in the *x* direction (Figure 6c). The lack of correlation up to this range confirms the absence of coupled shear responses in *x* and *y* directions during the calculation of the shear modulus. After shear failure at $\varepsilon_{xz} > 0.06$, a correlation of the shear response in *x* with that in *y* was observed.

S8. Nanoindentation

S8.1. Further Comparisons to Experimental Data. In experiments, a single MXene sheet fractured at ~38 nm of tip penetration, or 9.3% of 410 nm pore radius. Our results from reactive MD simulations agree with the experimental fracture data: substantial layer rip occurred (1) at 9.5 nm, or 11.2% of the 85 nm pore radius, (2) at 4.5 nm, or 15.0% of the 30 nm pore radius, and (3)

at 3.0 nm or 40% of the 7.5 nm pore radius (Figure 6d). This trend indicates larger relative displacements before fracture for smaller pores.

Refinements in the bond dissociation energy, which has an uncertainty of ±10%, can be made if needed upon further comparisons with experimental data in future studies.

S8.2. Limitations and Possible Extensions of Reactive Simulations. For the demonstration of reactivity in this paper (Figure 7), we do not adjust atomic charges or other IFF-R parameters up to bond dissociation. The neglect of polarization changes at the breaking site in this implementation is a limitation for certain reaction pathways. However, this assumption is sufficient to obtain stress-strain curves up to failure in high quality (see ref. [8]), especially for relatively nonpolar C-Ti bonds. The protocol maintains simplicity, ease of use, and achieves very good agreement with experiments (Figure 7). Earlier work also showed that polarization is a second-order effect[6, 64] and involves relatively small changes in atomic charges.[48, 65] Reactive force fields often use physically less justified charges before polarization so that both ground state and polarization are difficult to describe in an adequate way. IFF and IFF-R focus strongly on using experimentally interpretable and reproducible “principal” atomic charges so that adding further complexity in the simulation is firmly grounded on quantitative descriptions of chemical bonding and often found to be optional.

When necessary, IFF-R can be extended to include transitional parameters along a reaction path, i.e., between reactant parameters and product parameters. We discussed realistic descriptions of bond forming and dissociation behavior in ref. [8]. For example, we can define templates for follow-on reactions using the bond react scheme, including transitional parameters and bond cutoff points along the reaction coordinate with associated adjustments in charges, LJ, and other parameters to represent gradual dissociation and follow-on reactions. Hereby, dissociation and

follow-on reactions typically complete at 150% to ~200% original bond length. We have shown in ref. [8] for various materials that the inclusion of reaction templates is usually not necessary for stress-strain curves up to failure unless a very high accuracy is desired.

Regarding dynamic QEq/EEM methods and ReaxFF, we found these methods to underperform in the derivation of static (nonreactive) charges.[2, 8, 13] We see limited merit in using a reactive force field like ReaxFF that may not reproduce structural and energetic properties of reactants and products well by themselves (several times less reliable than IFF), and then using this model to simulate chemical reactions. We introduced a path to modeling reactivity and reactions in IFF-R that starts with meticulously validated representations of the reactants and products, knowing that predictions of reaction outcomes and kinetic parameters are highly sensitive to truthful representations of chemical bonding and energy differences. In this manner, we keep reaction paths aligned with expected reaction mechanisms and only as detailed as necessary. This protocol has been previously introduced for all materials classes (refs. [8, 66-69]), and is applied to MXenes in this study (Figure 7).

A particular limitation of ReaxFF and other reactive models is an overabundance of empirical parameters that combine with nonideal principal atomic charges (~50% deviation from dipole moments and IFF extended Born model scheme common). In such scenarios, no further addition of polarizable or other corrections terms can re-introduce physical realism into the model. As a result, polarization upon bond failure (a smaller effect) cannot be captured well even if incorporated via bond order terms. For example, ReaxFF leads to 20x too large of a slope in the MXene force-displacement curves in nanoindentation while IFF-R is close to experiment in both this curve and in fracture depth without using bond polarization (Figure 7 and discussion therein).

A major limitation of using DFT for chemical reactions is that it cannot reproduce bond dissociation curves of diatomic molecules, and consequently not for any other, more complex molecules. The energy curves go from the minimum bond energy to a maximum and then to negative values rather than zero (refs. [8, 70]). Therefore, bond dissociation energies and related predictions are not reliable unless methods such as CCSD(T) or higher levels of theory are used. Adding onto these uncertainties are the uncertainties of computed adsorption or aggregation energies of reactants and products. Accordingly, the results are underwhelming when DFT data is used to "fit" force fields. IFF-R overcomes this problem by using Morse potentials and can be modified to include further details based on experimental and high-level QM (e.g., CCSD(T)) data.

S9. Thermal Conductivity and Defect Models

S9.1. Discussion of Uncertainties in Thermal Conductivity. A wide range of experimental values for the in-plane thermal conductivity has been reported as a function of the thickness of the sample (Figure 8f). Results from MD simulations of pristine and defective MXenes are consistent with experimental data for few-layer MXenes within the uncertainties and demonstrate a large impact on the type and concentration of defects. The models feature realistic in-plane motion, supported by vibration constants and Young's moduli in agreement with experiments, and contributions by surface terminal groups have been validated via contact angles (as a proxy for unknown cleavage energies from measurements). Even though MD simulations have no explicit electronic component of thermal conductivity, the phonon signature appears to be accurate. Otherwise, we would expect underprediction of the heat transport coefficients.

Predictions of thermal conductivities using the semi-classical Boltzmann transport equation (BTE) require phonon dispersion calculations from DFT.[71] The reliability of these calculations

depends on the chosen density functional and involves tradeoffs. The popular PBE density functional, for example, leads to high uncertainties for several key properties of $Ti_3C_2T_x$ MXenes (Table 1). The 65-fold overestimation of the computed in-plane thermal conductivity by DFT/BTE as compared to monolayer experiments (Figure 8f) is likely related to these uncertainties. After adjustments for defects that help explain a 3-fold to 4-fold overestimate (Figure 8c, e), the DFT/BTE results are still ~15 times too high.

Since phonon momentum transfer occurs via nuclei and electrons and depends on the mean free path *l*, large phonons with micro-scale wavelengths do not contribute to thermal conductivities calculated in MD simulations unless large models are used. Electronic components require a suitable simplified representation, e.g., via accurate vibration and elastic properties. As our results show, IFF MD simulations show promise for comparisons across length scales and between nanomaterials. DFT/BTE results appear less reliable, and either method requires monitoring of assumptions and potential systematic uncertainties.

Simulation results for out-of-plane thermal conductivities have fewer uncertainties. Phonon path lengths and electronic components are expected to have much less impact in the out-of-plane direction, and the thermal conductivity computed with IFF MD has small deviations from experimental measurements (Figure 8c). The out-of-plane thermal conductivity for common mixed -OH/-F coverage is ~1.5x the value observed in laboratory measurements (0.56 vs. ~0.38 W m^{-1} K^{-1}) and likely close to the maximum in the absence of defects. As MXenes are prepared by acid treatment of the corresponding MAX phase with HF, subsequent rinsing may leave acidic surface groups such as OH_2^+ X^- and further ions intercalated between MXene layers, e.g., protonated ion pairs $(Ti_3)OH_2^+$ F^-. Such occlusions could increase average interlayer distances and decrease momentum transfer. Also, control of the plane orientation of MXenes can be less than

perfect in experiments, with some disorderly layers representing a mix of out-of-plane and in-plane directions. Further characterization of layer orientation, regularity, and defects in interlayers in experiments, paired with simulations, are promising to fully quantify thermal conductivities.

S9.2. Defect Models. To understand the in-plane properties better, we have included 3 models of common defects in MXenes:[72] (1) missing internal C atoms known from parent carbides such as TiC and Ti_3C_2, (2) missing surface groups Ti-X (Ti-F in our case), (3) and pinholes ("nanoholes") of ~1 nm size.

To build chemically and physically realistic models of low-energy defects, the stoichiometry, charge neutrality, and significant coordination numbers (at least 2 or 3 to avoid dangling bonds) need to be observed. For C vacancies in the inner layer, the stoichiometry of the defect formation reaction is straightforward: TiC → TiC_{1-x} + x C, whereby the excess carbon atoms are removed from the model. We used a supercell of 12.202x12.202x19.86 $Å^3$ with 100% -F coverage ($C_{64}Ti_{96}F_{64}$) to introduce these defects. We randomly removed 6 out of the 64 C atoms in the inner layers (charge of -0.35e each) and distributed the remaining charge difference of -0.35e over the 6 connected Ti atoms for each of the missing C atoms (-0.35/6e added onto the native Ti charge). In each such neighbor shell, the changes in charge were: (i) 3x0.35e for center Ti → 2x0.292e and 1x0.291e, (ii) 3x0.675e for surface Ti → 2x0.617e and 1x0.616e. (iii) If 1 center Ti atom was connected to two carbon defects, we applied two additive charge reductions: 0.35e → 0.292e → 0.233e. These charge increments resp. decrements can be automated via scripts. In this process, it is important to verify that the local and global net charge balance is exactly zero (0.0000e). The coordination number of the Ti atoms next to C vacancies was reduced from 6 to 5, and in rare instances to 4. These boundary conditions correspond to C vacancies with the lowest energy. The percentage of C atoms removed was 9.4% (6/64), equal to 2.7% of total atoms.

For the second type of defect, Ti-F defects on the surface, the stoichiometry of the formation reaction is: F-Ti-C-Ti-C-Ti-F → F-Ti-C-Ti-C + Ti-F. These defects may be less common than C vancancies. It is important to recognize the stoichiometry of the MXene inner carbide layer $C_{0.5}$-Ti-$C_{0.5}$, and of the two integrated outer layers on each side F-Ti-$C_{0.5}$ and $C_{0.5}$-Ti-F. Removal of a single Ti atom as a vacancy requires removing one F ion at the same time to maintain a low-energy defect stoichiometry. Removing the statistically attached carbon atom is optional since a Ti vacancy is defined as leaving the carbon atom in place. Accordingly, we removed 4 Ti-F pairs out of 64 Ti-F pairs on the surface in a random distribution. Each Ti(0.675e)-F(-0.5e) pair has a net charge of +0.175e that was distributed over the 3 C atoms bonded to the removed Ti atoms for electroneutrality. For each neighbor shell of Ti, the charges were: 3x-0.35e (carbon layer) → 2x -0.292e and 1x -0.291e. As a result, the coordination number of C atoms next to the Ti-F groups was reduced from 6 to 5. This coordination corresponds to TiF vacancies with the lowest energy. The percentage of surface TiF groups removed was 6.25% (4/64), equal to 3.6% of total atoms.

For the third type of defect, pinhole (or nanohole) cluster defects, we utilized a larger cell to begin with: 24.404 x 24.404 x 19.86 $Å^3$, corresponding to $C_{256}Ti_{384}F_{256}$ that contains two stacked MXene layers. The stoichiometry of these more complex pinholes is a combination of TiC vacancies (Schottky defects) in the inner layer, TiF vacancies in the outer layer, and some C vacancies in the inner layer, all in the same area. In the bilayer super cell chosen, we introduced (1) 5 TiC defects, 3x2 TiF defects (= 3 on each side), and 3 C defects in the upper layer, and (2) 5 TiC defects, 3x2 TiF defects (= 3 on each side), and 6 C defects in the lower layer, creating two distinct pinholes in different locations of each layer. The model features all surface Ti with at least 2 out of 3 bonds to C, i.e. no Ti with 1 dangling bond. Inner carbon atoms retain at least 4 out of 6 bonded neighbor atoms, and in few cases 3. The charge equilibration after removing the atoms

needed careful attention, working with exact charge increments (resp. decrements) in the new coordination environments to maintain exact zero local charge and exact zero global charge balances. Hereto, we used charge increments of -0.35e/6 for each missing bond around removed C atoms (4x-0.058e and 2x-0.059e), charge increments of +0.35e/6 for each missing bond around removed inner Ti atoms (4x0.058e and 2x0.059e), and charge increments of -0.35e/6 for each missing bond (less than 3) around removed outer Ti atoms, starting with +0.675e for 3-fold bonded and subtracting -0.058e or -0.059e for a missing bond (in a ratio of 2x -0.058e and 1x -0.059e). The total number of missing atoms is accordingly 19 C atoms, 22 Ti atoms (10 inner, 12 surface), and 12 F atoms, equal to 5.9% of total atoms.

The 3D periodic model structures of all three defect types were subsequently relaxed by 200 steps energy minimization (smart minimizer in the Discover program) and upscaled to the desired size using supercells.

S10. Computational and Experimental Methods

Molecular models of MXene structures were prepared using X-ray data[73] and the Materials Studio Graphical User Interface.[64] Force field parameters were derived from scratch for the $Ti_3C_2T_x$ MXene with two surface terminations (-OH and –F) following IFF protocol and validation by experimental lattice parameters, contact angles, Raman spectra, and Young's moduli.[2, 8] Molecular mechanics and molecular dynamics simulations of the properties of interest were carried out using Materials Studio for smaller test systems, the Large-scale Atomic/Molecular Massively Parallel Simulator (LAMMPS) for large-scale production simulations. We employed the NPT ensemble and, in some cases, NVT ensembles (e.g., cleavage energy, nanoindentation). Visualization of the

molecular models, dynamic trajectories, and image generation involved Materials Studio and the OVITO program.[80] Data analysis was conducted using tools in these programs and custom scripts, provided in the Supporting Files (written in MatLab, OVITO, and python). Uncertainties were obtained using block average data in MD trajectories and multiple simulations of the same properties to ensure reproducibility.

S10.1. Model Building. Unit cells for the $Ti_3C_2T_x$ MXenes were built using atomic positions provided in X-ray crystal structure data in ref. [73]. The space group is P63/mmc, and different surface terminal groups *T* occupy the same surface sites above the outer layer of Ti atoms (Figure 1). Our default mixed surface composition that reflects average MXene compositions used in experiments was 47% -OH and 53% -F, whereby the exact surface composition is often not reported in measurements and expected to deviate by 5% to 10%. 3D periodic supercells up to 10 nm lateral size were built to incorporate different statistical ratios of -OH and -F surface groups, ranging from 0% to 100% -OH coverage, as needed for calculations across the range of surface chemistry. Hereby, -OH groups could be easily replaced with -F groups or other surface functionalization of interest (-Cl, -Br, O-CH_3) in the desired ratio. For large systems, random seeding of surface functional groups was achieved using a custom MATLAB script (provided in the Supporting Files). Elemental identities, atomic masses, atomic charges and atom types (force field types) were assigned to every atom consistent with the IFF definitions. For the analysis of thermal conductivity and contact angles, we used larger models composed of 64x8x2 and 48x48x1 unit cells, respectively, and single layers up to approximately 200 nm wide in length for nanoindentation.

S10.2. Force Field Development. Force field development utilized initially small multiples of the unit cell, about 2 nm in all directions. This length is past the range of electrostatic and van-

der-Waals interactions in electroneutral systems. We utilized the energy expression of the INTERFACE force field (IFF) in CVFF format. The workflow included the (1) initial assignment of charge distributions following the IFF protocol,[2, 13] (2) definition of atom types, (3) assignments of LJ parameters consistent with the chemical environment,[2, 74] (4) assignment of bonded parameters and angle parameters consistent with X-ray data and vibrational spectroscopy, (5) followed by iterations until lattice parameters replicated experiment within <0.5% deviation (Figure 2f). For example, the lattice height *c* was affected by partial charge assignments on -OH surface groups, while lattice parameters *a* and *b* were affected by bond angles and the angle bending constant. (6) Due to lack of experimental data for surface energies, we then used computed contact angles with water and CH_2I_2 to refine atomic charges, especially near the surface groups until agreement with experimental data was reached (better than ±5° reliability is often not possible in experiment). Agreement here provides validation for energy differences (equivalent to cleavage energies). (7) We revisited the Ti-C bond stiffness constants, in both IFF and IFF-R models, to reproduce both the experimentally determined in-plane Young's modulus and the approximate Raman spectra, (8) Iterative adjustments of LJ parameters were carried out to ensure lattice parameters remained within specifications. In total, more than 1,000 simulations were carried out to optimize the force field parameters. The simulations included short energy minimizations and long MD simulations over 10 ns, for example, for feedback on contact angles.

The assignment of atomic charges and LJ parameters, as well as bond and angle vibration constants, was aided by comparison with reference data and chemical analogies to over 100 compounds with rigorously validated parameters in IFF. As a result, the parameters quantitatively represent chemical bonding (covalent versus ionic), relative atomic sizes, polarizability, and exclusion rules for nonbonded interactions between 1, 2 and 1, 3 bonded atoms. The parameter

refinement was carried out under consideration of native combination rules in multiple force fields, i.e., CVFF/OPLS-AA, CHARMM/AMBER, and PCFF/COMPASS (including also 9-6 LJ parameters) without loss in accuracy (see separate entries for LJ parameters in Table 2 and Table S1). We also considered different treatment of nonbonded interactions between 1,4 bonded atoms in some of the above force fields. 1,4 bonded interactions are only present in the internal carbide layer of the MXenes and associated with high bond and angle stiffness. Therefore, differences in treatment of associated nonbond interactions are effectively negligible.

The proposed IFF and IFF-R parameters have a clear interpretation of atomic charges and LJ parameters, lead to deviations of <0.3% in density relative to experiment and agree with experimentally reported contact angles and Young's moduli. We cover chemistry space previously unavailable in IFF, CVFF, CHARMM, AMBER, OPLS-AA, PCFF, CFF and COMPASS parameters, and achieve full compatibility with organic and inorganic compounds included in these force fields.

The parameters were then used for predictions of cleavage energies, contact angles, biomolecular adsorption, mechanical and failure properties, and thermal conductivity for custom surface chemistries, as well as first assessments of the influence of defects (Figure 2f).

S10.3. Protocols of Molecular Dynamics Simulations. Protocols for molecular dynamics simulations follow standard settings for IFF: 12 Å spherical cutoff for LJ interactions, high accuracy electrostatics (standard of 10^{-6}, and at least 10^{-4}), 1 fs time step, 298.15 K, and standard pressure. Simulation times were up to 50 ns for individual trajectories. For thermostats and barostats, we recommend velocity scaling and Parinello-Raman resp. Nove-Hoover chains. However, any reasonable choice leads to the same results within error bars.

We developed specific protocols for effective computation of the shear response, thermal conductivity, and nanoindentation. Protocols to compute lattice parameters, cleavage energies, contact angles, and elastic moduli followed those previously reported and extensively tested.[7, 46] Simulations were performed using LAMMPS and NAMD. Details are described in the following subsections.

S10.4. Molecular Geometry, Lattice Parameters and Density. Molecular dynamics simulations in the NPT ensemble were carried out to obtain the equilibrium lattice parameters (lengths *a*, *b*, *c* and angles *α*, *β*, *γ*), density, and bond lengths between titanium and carbon atoms in comparison to X-ray data for the $Ti_3C_2T_x$ MXene (space group P63/mmc).[73] To build the 3D periodic models, the unit cell was replicated in the *a* and *b* directions to form an 8x8x1 super cell, with lattice lengths of *a*, *b* = 24.04 Å, and *c* = 19.86 Å. Side lengths of ≥ 2 nm ensure the elimination of finite size effects from periodic images of atoms in neighbor cells. The supercell was assigned a random distribution of -OH and -F groups on all surfaces in a 47-53 split.

An initial energy minimization with geometry optimization was carried out for 200 steps, followed by MD simulations in the NPT ensemble using a velocity rescaling thermostat[75] and Parinello barostat.[76] Less than 30 ps of simulation time were needed reach equilibration of the crystal lattice. Regardless, we carried out simulations for 10 ns and used over 1000 independent snapshots to compute average equilibrium lattice parameters, density, bond lengths, and bond angles.

An atomic overlap analysis of the average structure in MD simulations and the X-ray crystallographic structure (using Materials Studio's Superpose Structures tool) showed ~0.014 Å average root-mean-square deviation per atom.

S10.5. Raman and Infrared Spectra. The calculation of vibration spectra involved short MD simulations of 10 ps to record the velocity autocorrelation function (VACF). The VACF includes a superposition of vibrational modes active in experimental Raman and IR spectroscopy.[2, 25, 77] Experimental reference data for Raman and IR spectra were consistent across different experimental sources,[49-54] providing ideal input for validation and fine-tuning of bonded model parameters (unlike stress/strain data and elastic constants). Example scrips for the calculation of the vibration spectra are provided in the Supporting Information.

We utilized a 6x6x1 super cell for simulations, with cell parameters of *a*, *b* = 18.39 Å, and *c* = 19.86 Å. The model was assigned a random distribution of -OH and -F surface groups in a ~50/50 split. We used a MatLab script that modified the LAMMPS input data file (see Supporting Files). The surface groups had little to no impact on the titanium-carbon bond and angle peaks characteristic for MXenes and model validation.

Following initial energy minimization for 200 steps, MD simulations were carried out for 10 ps with recording snapshots and velocities every 2 fs. We used the NVT ensemble with equilibrium lattice parameters, and the Nose-Hoover thermostat. The VACF was calculated for all atoms over time using LAMMPS tools (alternative: Materials Studio Discover or Forcite), followed by a fast Fourier transform to obtain the vibration spectra using custom python and MatLab scripting (see Supporting Information). We overlaid the experimental IR and Raman spectra for ease of comparison by discretizing original data into points using the WebPlotDigitizer tool[78] and scaling the intensities (given in arbitrary units by default).

S10.6. Cleavage Energy. MD simulations were employed to capture the energy of surface creation, termed *cleavage energy*, by direct comparison of a bulk system with no surfaces versus one with surfaces.[25, 79] The cleavage energy is the actual surface energy and distinct from surface

energies calculated via the Young equation from contact angles. The latter protocol refers to already created surfaces that may have been subject to prior reconstruction or reactions with a solvent. We could not find prior experimental or computational data for the surface energy (cleavage energy) for MXenes. The cleavage energy is a critical proxy to validate energy differences and predict adsorption energies, binding mechanisms, activation energies, contact angles, mechanical, thermal, and other properties via MD simulations. All of these properties directly involve related energy differences or derivatives of these energy differences with respect to distance (deformation) and temperature.[2] (Since experimental data were not available, we used contact angles as a proxy for validation and predicted the cleavage energy for future use.)

We utilized 3D periodic 6x6x2 supercells with cell parameters of *a*, *b* = 18.39 Å, and *c* = 38.72 Å that contain a stack of four MXene layers. We computed the energy in equilibrium over a period of 50 ns using MD simulations in the NVT ensemble and equilibrium lattice parameters at 298.15 K and 101.325 kPa pressure (time step 1 fs, 12 Å cutoff for LJ interactions, PPPM method for electrostatics with high accuracy of 10^{-6}, velocity rescaling thermostat,[75] Parinello barostat[76] to obtain equilibrium lattice parameters in MD simulation). Then, a vacuum space was added in the *c* direction, bringing the total box height to 100 Å, which introduces two exposed surfaces at the top and at the bottom of the 4-layer structure. Then, MD simulations were run again for 50 ns with the same protocol to compute the average energy of the cleaved structure.

The cleavage energy, $\gamma_{Cleavage}$, was then calculated as the difference in total time-average energy of the cleaved structure, $E_{surface}$, and the bulk structure, E_{bulk}, normalized by the area of the created surfaces, $2 \cdot A_{surface}$:

$$\gamma_{Cleavage} = \frac{E_{surface} - E_{bulk}}{2 * A_{surface}}. \quad \text{(S6)}$$

The simulations were also repeated for a 2-layer system (~19.4 Å) thickness and led to the same results as the thickness remains larger than the range of nonbonded interactions. The calculations were repeated for different surface termination, and we typically found the fluctuations and uncertainties increased as the system size was larger (e.g., 4 layers instead of 2 layers).

S10.7. Contact Angles. The calculation of equilibrium contact angles was performed utilizing the simulation protocols of ref. [7] and the older method of ref. [57]. Both methods lead to the same results within the uncertainty. The latter method[57] defines an analytical relation between the center-of-mass location of a probe liquid droplet with known number density and its unique contact angle. Experimental reference data were chosen from the contact angle measurements of a $Ti_3C_2T_x$ MXene thin film by Zukiene et al.,[80] who obtained contact angles in H_2O and DIM (CH_2I_2) as probe liquids, simultaneously characterized the distribution of surface groups, and provided uncertainty estimates. The results of this work are consistent with other earlier studies.[80-84] Example scripts for calculating average contact angles from a series of snapshots from MD simulations are provided in the Supporting Information.

We utilized 72x72x1 super cells of the MXene with cell parameters of a, b = 219.65 Å and c = 19.86 Å that provide ample surface space for spreading of the droplets. Vacuum space equal to 100 Å was added above the bilayer MXene, bringing the height of the simulation cell up to c ~ 120 Å. The surface termination varied from fully -OH to fully -F and included multiple mixed ratios. The mixed surface terminations were implemented as random distributions via a MatLab script provided in the Supporting Files that modified the LAMMPS input data file. The script also adds a liquid droplet into the center of the simulation cell, with the bottom of the droplet initially positioned 5 Å above the MXene lattice surface. Water models included TIP3P[85] and similar flexible SPC water models, and DIM models were used as previously validated in IFF.[7] Both water

models perform nearly identical within error bars.[7] Droplets were sized at 1880 molecules for H_2O and 940 molecules for DIM, underwent energy minimization and equilibration during MD simulations in the NPT ensemble before being inserted into the model for simulating the contact angles. Tests as a function of droplet size showed that more than 1000 molecules in a droplet lead to negligible errors due to line tension effects (Figure S4d).

The bottom atomic layer of the MXene was fixed in space to reduce out-of-plane oscillations. After a brief energy minimization, systems composed of the MXene slab and the droplet were subjected to MD simulations in the NVT (canonical) ensemble at 298.15 K using the Nose-Hoover thermostat. Fixed volume helped to prevent expansion or collapse of the vacuum space between periodic repeats of the 3D periodic system. The total simulation time was 5 ns created an equilibrium trajectory that allowed for equilibration and fluctuations. The number of molecules staying in the droplet (as a few enter vapor phase) was monitored to determine the center of mass.

Analysis involved MatLab scripts to obtain average contact angles over time from at least 100 independent snapshots. The method by Hautman et al.[57] treats the liquid droplet as a portion of a sphere and two analytical relations relate the center of mass height, z_{cm}, to the contact angle θ:

$$z_{cm} = 2^{-4/3} R_0 \left(\frac{1-\cos\theta}{2+\cos\theta}\right)^{1/3} \frac{3+\cos\theta}{2+\cos\theta} \quad \text{(S7)}$$

$$R_0 = \left(\frac{3N}{4\pi\rho_0}\right)^{1/3} \quad \text{(S8)}$$

Hereby, N is the number of droplet molecules and ρ_0 is the number density of the fluid (number of molecules per unit volume). This number density was assumed constant throughout the macroscopic droplet. Monitoring z_{cm}, N (and ρ_0) over the simulation timesteps enabled the calculation of a contact angle using Equation (S7) with an iterative Newton solver. Contact angles

fluctuated initially within 5° to 10° and later within a smaller range. We report the average contact angle from the latter, equilibrium part of the MD simulations.

Experimental contact angles in equilibrium also undergo significant fluctuations, ±5°, which is similarly observed in MD simulations at a smaller scale (Figure S4).[56] Overall, computed contact angles agree with experimental data within typical error bounds.

S10.8. Dopamine Binding and Assembly: QCM-D and UV/Vis Measurements. Application of MXene to QCM Chips: Quartzpro QCM5140TiAu120-050-Q QCM chips were coated using an interfacial assembly method for $Ti_3C_2T_x$. In a 9.5 cm diameter glass petri dish, 40 mL of MilliΩ H_2O and 2 mL of the MXene stock solution were combined. 5 mL of ethyl acetate was added dropwise around the edge of the dish. The ethyl acetate began to evaporate immediately, inducing Rayleigh-Benard convection currents.[86] Film formation began at the liquid-air interface. Evaporation was allowed to go to visible completion before the film was scooped onto the QCM chip. Samples were allowed to dry horizontally under ambient conditions for 24 hours. The coated chip was stored in a low-permeability pouch sealed in argon until ready for use.

QCM-D Measurements: A Q-Sense E4 quartz-crystal microbalance with dissipation (QCM-D) was utilized to monitor binding of dopamine solutions. Dopamine hydrochloride was purchased from Sigma Aldrich and prepared as solutions with a concentration of 1 and 100 µg/ml in deionized water. The solution assumed a pH value of 5 before and during contact with the MXene chip to prevent auto-polymerization of dopamine that occurs at basic pH. For QCM measurements, the temperature was controlled at 23°C to minimize noise and frequency drift (Actual 23.400°C ± 0.003°C), the flow rate was maintained at 0.17 mL/min using a high precision peristaltic pump, and the 3rd overtone frequency was monitored vs. time. Before exposure to

dopamine solutions, the MXene coated QCM chip was exposed to doubly deionized water at 0.17 mL/min and equilibrated until a stable baseline was achieved (±0.1 Hz or ±1.155 ng/cm^2). After equilibration, the deionized water was replaced with 1 μg/mL and then 100 μg/mL of dopamine solutions and continuously monitored for frequency changes. Finally, the concentration-dependent mass accumulation of dopamine on a solution-processed MXene coated QCM chip was determined by applying the Sauerbrey equation and plotting as a function of time.

The concentration-dependent mass accumulation of dopamine on a solution-processed MXene coating on a gold coated QCM sensor chip (QuartzPro) was acquired using the Sauerbrey equation to convert frequency into mass as a function of time using a flow rate of 0.17 mL/min.

UV-Vis Spectroscopy: UV spectra were measured for MXene in water, dopamine in water, and MXene films after exposure to dopamine solutions at two concentrations of 1 mg/ml and 10 mg/ml. A Cary 5000 UV-Vis-NIR Spectrometer measured optical extinction at a scan rate of 600 nm/min with a detector and grating change set to 900 nm to avoid interference with the plasmon band of MXene around 800 nm. The dopamine peak at 280 nm was observed on multiple samples, and is repeatable, showing binding to the MXene particles.

S10.9. Dopamine Binding and Assembly: MD Simulations. MD simulations were employed to study the adsorption mechanism and energy of a single dopamine molecule in solution and the assembly mechanism of multiple dopamine molecules corresponding to a surface coverage up to 3 molecules per nm^2 (~90 ng/cm^2) as observed in QCM-D measurements. We employed the CHARMM36 parameter types for dopamine and the NAMD program[87] for MD simulations. For the calculation of adsorption energies, two simulation boxes with dopamine adsorbed and desorbed were used to compute differences in average adsorption energies (utilizing only time blocks with dopamine adsorbed for averaging the energy in the adsorbed state).[88]

We utilized a 8x9x1 super cell for the MXene substrate, which was expanded to 79.86 Å in the *z* direction to create space for the electrolyte and dopamine. The resulting rectangular cell with side lengths of *a* = 48.808 Å, *b* = 47.5524 Å, and *c* = 79.86 Å was filled with water (flexible SPC model) to simulate the system at a density near 1.000 g/cm^3 for the aqueous electrolyte. The position of the 53% F and 47% OH groups on the MXene surfaces were randomized. To represent a pH value to 5 as in experiments, four of the OH groups on each side of the layer were protonated by HCl, resulting in H_2O^+ Cl^- pairs. For the single dopamine systems, a single dopamine hydrochloride ion pair (DAH^+ with Cl^- counterion) was placed in a variety of starting orientations on and off the surface. For the full accumulation simulations, PACKMOL[84] was used to place 80 DAH^+ Cl^- pairs in a 9 Å thick layer above one side of the surface.

For adsorption of single dopamine in solution, 12 simulations with varying starting orientations were run for 10 ns each using NPT molecular dynamics at 298 K. The model systems with multiple dopamine molecules were run with the same parameters for 50 ns (protocols are the same as in earlier studies[59]). Furthermore, a simulation box of water only and of a single dopamine molecule in water (using the same number of water molecules as in the combined system with MXene at equilibrium density), were run for 30 ns. The Langevin thermostat was used for temperature control at 300 K and 310 K, and the Langevin-Piston barostat was used for pressure control at 1 atm. A cutoff at 12 Å and pair list distance of 14 Å were used for LJ interactions, and the PPPM method for high accuracy electrostatics (10^{-6}).

The resulting trajectory files were visualized using the VMD program (version 1.9.4a53)[89]. For each of the single dopamine simulations, binding attachment was determined both visually in VMD and using Python 3.10.16, using less than 6 Å distance of the center of mass of dopamine to the surface atomic layer of (O, F) in the MXene. Binding energies and percent of time in different

positions relative to the surface were determined from the block averages of the 12 simulations, considering the following distances: (1) dopamine was attached to the surface (as above), (2) dopamine was near the surface (6 to 12 Å distance of the center of mass of dopamine to the surface atomic layer of (O, F) in the MXene), or (3) free floating in solution (>12 Å of the same distance). The binding energy was the difference in average energy for the given state and the average energy of dopamine when fully detached from the surface (>10 Å away from surface functional groups). For multi-molecule adsorption, the adsorption energy was calculated using $\Delta E_b = E_1 + n\,E_w - E_3 - n\,E_2$, where E_1 is the average energy of an MXene sheet in water with attached dopamine molecules, E_w the average energy of a box of water without any other additions, E_2 the average energy of a water box with a single dopamine, and E_3 the average energy of a MXene sheet in water. Hereby, keeping equilibrium densities of all phases was important as energies are sensitive to volume changes.

Average energies calculated from the simulations were also corrected for equal temperature (298.000 K) using the simulated heat capacities of the systems. The heat capacity was determined by simulations of the same system at 310 K and at 300 K using the difference in average energy according to the definition, $C_p = \frac{E_{T_2} - E_{T_1}}{T_2 - T_1}$. The binding energy equals the binding enthalpy due to negligible volume change upon adsorption.

S10.10. Linear Elastic Mechanical Properties. Analysis of the elastic stress/strain response of the $Ti_3C_2T_x$ MXene used standard IFF MD simulation protocols from earlier work.[5, 90] Hereby, an induced strain causes a measurable stress response including multiple elements of the stress tensor. Due to the unique 2D topology of MXenes, we characterized tensile and compressive stress-strain curves along the in-plane and out-of-plane directions.

Prior studies from experiment, quantum mechanics, and MD simulations varied widely in their results, and no prior simulation model was validated across multiple critical properties (structures, surface energy/contact angles, and vibration spectra), which is important for consistency. While we surveyed a number of experimental reference data as a potential benchmark,[10, 11, 18, 61, 63] the combination of bonded and nonbonded parameters in the force field should also reproduce the experimental Raman spectra at the same time. Consistent with this rationale, we chose an experimental study using nano-indentation on a single MXene layer as the most trustworthy value for the tensile and compressive in-plane modulus.[18]

We utilized a 6x6x1 super cell with lattice lengths of a, b = 18.39 Å, c = 19.86 Å, and a series of surface terminations of 100% -OH groups, 50/50 mixed -OH and -F groups in a random distribution (see MatLab scripts in the Supporting Files), and 100% -F groups. Initial energy minimization was carried out for 200 steps, and the systems were subsequently subjected to MD simulations in the NPT (isobaric-isothermal) ensemble for 2 ns using a Nose-Hoover thermostat and barostat at 298.15 K and 101.325 kPa. These simulations aimed at reaching equilibrium lattice parameters and angles at zero stress as a reference point relative to which elastic constants under applied strain were to be computed. Depending on the direction of strain of interest, the lattice would then undergo contraction or expansion, causing a corresponding reaction in the stress tensor. Upon the applied strain program, all other cell dimensions were allowed to shift freely. We carried out a series of MD simulations with applied tensile or compressive strain rates of $1 \cdot 10^{-9}$ per fs in the desired directions over a period of 5 ns that resulted in 0.5% compressive or tensile strain ($5 \cdot 10^{-3}$) in the desired direction at the end of the simulation.

Stress-strain curves were calculated by tracking lattice deformations and the selected component of the pressure tensor over time, e.g., σ_{xx} if strain had been applied in the x-direction.

Hereby, the simulations were orders of magnitude slower than necessary to remain in equilibrium and did not depend on the strain rate, since a typical equilibration time for a new strain setting is only around 20 ps for a conformationally rigid material like an MXene.[2, 90] The resulting cloud of points was fitted with a linear fit to extract the average slope. This slope equals the elastic modulus of the MXene in this given direction of strain. Example scripts for tensile property calculations after the completed MD simulations are provided in Supporting Files.

S10.11. Shear Properties: Linear Elastic Shear Properties, Slip, and Shear Failure. We developed protocols suitable to study shear strain-shear stress responses up to failure, partly based on similar studies for other 2D materials.[26, 90-92]

We utilized a super cell of 12x12x1 unit cells with side lengths of *a*, *b* = 73.212 Å and *c* = 19.86 Å. This size was better suited than smaller models to study layer slippage. The models were terminated with 100% -OH groups and 100% -F groups, respectively, fully periodic in the three directions, and all atoms were flexible.

Two different protocols were utilized to determine the shear modulus and the shear strength, namely, (1) a series of static shear stress-shear strain curves (NVT) and (2) dynamic shear stress-shear slip simulations (NPT).

(1) For the determination of shear modulus, the simulation box was subjected to a range of successive shear displacements between 0 and 1 Å (0% to ~5% shear strain) in the *xz* direction, i.e., in the *x* direction parallel to the plane, enforcing shear strain. We then monitored the induced shear stresses while keeping the shear displacement fixed during MD simulations using the NVT ensemble for 50 ps. This method was suited to determine the elastic part of the response at low strain, whereby we kept the volume constant and allowed no expansion in the *z* direction.

Applying displacement and monitoring shear stresses was an effective method to elucidate the shear modulus because the deformation range of the MXenes with elastic behavior was quite small (<1 Å). A shear stress-strain relation was obtained by deforming the box every 0.125 Å (ε_{xz}) and recording the induced shear stresses (σ_{xz}). We observed a clear linear relationship at low shear stress and report the shear modulus as the slope of this curve. Higher strain eventually lead to yield and slip, although this simulation setup prevents relaxation by lateral or vertical motion. Therefore, the maximum shear stress represents a barrier height rather than the dynamic shear strength (which is lower). After the shear strain reaches a maximum at ~0.8 Å displacement, it decreases as the surface groups between two layers then shift from unfavorable overlap with each other into a new local energy minimum. This protocol could be augmented, if needed, to capture NPT conditions by allowing vertical expansion at every fixed shear strain, for example, by using a series of changing, fixed cell angles α or β that define fixed shear strains and employ MD simulations in the NPT ensemble under these cell angle constraints.

(2) For the investigation of slip and shear strength, we fixed the positions of one MXene layer (except surface groups) and applied lateral shear forces to every other MXene layer at different levels, corresponding to shear stresses of 0 to 200 MPa (Figure 6a). At each setting of shear forces, MD simulations were carried out for 50 ps in the NPT ensemble utilizing a Nose-Hoover thermostat and barostat at STP conditions. Hereby, the volume was flexible and expansion in the z direction was allowed. The shear forces were systematically increased until the dynamic layers slipped over the static layers, exhibiting shear yield, both in the form of initial static, stepwise slip, and full dynamic slip (sliding) at higher shear stresses (Figure 6b). This second, dynamic protocol allowed surface groups to respond to stress events and induce relative motion of the layers. Relative motion of the layers was not possible in the static stress-strain setup of the first method.

A constant force was applied in the lateral (x) direction over the entire simulation to provide a defined value of the shear stress. The shear strength was obtained by observing the likelihood of slip over time and steady-state layer velocities in the dynamic layers were recorded once continuous layer sliding was observed (see MatLab scripts for analysis in the Supporting Files). When static slip was detected via repeated stepwise motion, the associated average shear stress can be considered the shear strength, and when continuous stepwise motion was recorded, the associated average shear stress can be considered the shear strength for continuous slip.

Due to the statistical variability in slip motion, we replicated 10 identical systems for both –OH and –F surface treatments and subjected them to the dynamic slip simulations. The onset of sliding yield (effective shear strength) occurred within the range of 15-30 MPa for the fully fluorinated (-F) MXene, and 50-155 MPa for the fully hydroxylated (-OH) MXene. In both cases, the shear strength is much lower than the tensile strength in any direction. Example scripts to calculate shear properties from LAMMPS trajectories are provided in the Supporting Files.

S10.12. Nano-indentation and Fracture. Our nanoindentation protocol sought to balance accurate replication of AFM experiments[18] without going to the full physical scale. In experiments,[18] nanoindentation was conducted by placing a single layer of (47% OH, 53% F) terminated MXene across a plate with 820 nm pores. The simulated AFM tip would then penetrate the MXene monolayer held across the pores. In the simulations, we utilized three analogous but smaller systems (15 nm, 60 nm, 170 nm) to evaluate the accuracy, scaling with pore size, and maintain a modest computational load.

The AFM tip in the simulation was generated by trimming a Fd3m diamond cubic lattice of sp^3 carbon atoms into a 30° cone terminating in a single atom (using IFF parameters for diamond). Dimensions of the cone are an end diameter of 35 Å, a height of 35 Å, and a cone angle of 30

degrees. The tip was placed 4 Å above the center of a MXene monolayer, with constrained (fixed) atoms outside of a given pore radius to simulate indentation on a pore plate. The fracture depth was recorded as the vertical cone tip displacement when bond dissociations first occurred and is likely to have a higher resolution than in experiment. For the purposes of failure simulations, we used the IFF-R parameters that utilize a reactive shifted Morse potential for the C-Ti bonds instead of a nonreactive harmonic bond stretch potential.

Triclinic unit cells were replicated in the *a*, *b* directions to form a 24x24x1 single MXene layer with side lengths of ~146 Å. A larger system was created by replicating upsizing this model 4 times in both directions to form a 96x96x1 single MXene layer with side lengths of ~584 Å. Further, an extra-large system with 250x250x1 unit cells was built that had side lengths of ~1520 Å. As seen in Figure 7a, b, a circle with a maximum possible radius in the *x*-*y* plane was then inscribed about the cone tip to define a circular section of the MXene lattice for deformation. All atoms outside this circular section were fixed.

Two protocols were utilized in the nanoindentation simulations, a non-reactive protocol for force tracking and a reactive IFF-R protocol for visualizations of fracture. In AFM experiments, forces are applied to the AFM tip via an actuator that moves the diamond tip into contact with a sample. To simulate this process, we moved the rigid cone downwards in regular increments of 0.1 Å and tracked the response of *z*-forces in a localized area of the AFM cone tip. Cone atoms were removed from NPT dynamics but could still experience forces, under assumption that diamond tip deflections were negligible. In the reactive IFF-R protocol,[8] titanium-carbon bonds were allowed to break via the shifted Morse potential, and all other simulation details were the same.

The computed cone-tip forces were plotted against cone displacement distances and overlaid with experimental values[18] utilizing the WebPlotDigitizer for the overlay.[78] Vertical displacements were recorded with respect to the average initial coordinate of the top layer of MXene titanium atoms. During the reactive simulations, forces in the cone tip saw larger fluctuations as bonds broke, so the non-reactive forces provided a cleaner comparison to experimental curves taken from larger systems and were used to generate force-displacement curves. Reactive simulations were visually analyzed to determine the initiation and propagation of MXene fracture.

S10.13. Nanoscale Thermal Conductivity. The model setups involved a Fourier-law approach in which four MXene layers (10 and 20 nm in length) were stacked in the in-plane direction (Figure 8a), and thirty-two MXene layers (4x4 nm squares) were stacked in the out-of-plane direction (Figure 8b), both in 3D periodic simulation boxes. We used the original hexagonal supercells with 120° angles.[73] A portion of atoms at each end were constrained to avoid periodic interactions, adjacent to temperature-controlled regions (~2.5 nm long, 350 K and 250 K) separated by energy-constrained regions to generate thermal gradients across the system as heat flowed from hot to cold sides. MD simulations in the NVE ensemble were used to equilibrate the temperature gradient and record the steady-state heat flux and thermal conductivity in both directions.

In this manner, computation of the thermal conductivity of nanoscale $Ti_3C_2T_x$ was based on analyzing a steady-state temperature gradient across a bulk slab of MXene, utilizing Fourier's law.[93] MD simulations were used to examine thermal conductivities in both in-plane and out-of-plane directions for fully fluorinated (-F), fully hydroxylated (-OH), and 50/50 surface terminations. Calculations of in-plane thermal conductivity involved two systems of different size, one 100 Å long, the other 200 Å, to explore the impact of longer wavelength lattice vibrations (phonons). We also included 100% F covered MXene models with three common types of defects.

The defects led to strong reductions of the in-plane thermal conductivity and included randomly missing internal C atoms (9%), TiF surface groups (6%), and pinholes (6% of atoms) (Figure 8d, e).

At the nanoscale, thermal conductivity was expected to increase with length, so that unit cells were replicated in the *b* and *c* directions to form a "L"x8x2 super cell, with lattice lengths "L" extending from 32 to 64 units. Therefore, the largest 3D model slab had dimensions of *a* = 390.40 Å, *b* = 48.81, and *c* = 39.72 Å. Both ends of the slab were fixed with 15 Å lengths to provide 30 Å buffer zones in the periodic slabs.

Two independently temperature-controlled regions connected to the fixed ends were defined, both at 15 Å thickness, that maintained a constant temperature via Nose-Hoover thermostats using the NVT ensemble (see Figure 8b, c). One region was controlled to be hot at 350 K, while the other end was set as a cold region at 250 K. The center portion between the two ends was subjected to MD simulations for an extended time (>20 ns) in the microcanonical ensemble (NVE), different from the NVT ensemble in the hot and cold slabs. The NVE ensemble maintained constant total energy. Initially, an equilibration time of 0.5 ns developed a steady state temperature gradient. Its slope directly correlated with thermal conductivity λ via Fourier's law:[93]

$$\lambda = \frac{Q/A\Delta t}{dT/dz} \tag{S9}$$

Equation (S9) corresponds to the definition of macroscopic thermal conductivity as used in experiments. The terms were straightforward to compute, whereby $Q/\Delta t$ is the heat flow into the hot region at steady state as monitored in the MD program (e.g., LAMMPS), and A is the cross-sectional area. The temperature gradient dT/dz is linear in the center of the slab and assumes a

constant value after steady state is reached. The center slabs subjected to NVE MD simulations were discretized into 24 sub-regions to monitor the temperature profile.

Due to the triclinic crystal structure of the MXenes, all sub regions of the in-plane temperature slabs were rotated by a 30° tilt to account for the proper path length vertical to the cross-sectional area. The uncertainties in thermal conductivity were obtained from error bars in heat flux and temperature gradient. The thermal gradient was subject to larger fluctuations than the heat flux, and the total uncertainties are represented by the error bars in Figure 8. Scripts for the calculation of thermal conductivities are provided in the Supporting Files.

S10.14. Tradeoffs and Opportunities for Further Development of IFF and IFF-R. It is well-known in the molecular dynamics community that force field parameterization often involves trade-offs.[94] Our reasoning for broader compatibility and utility across multiple properties lies in the derivation of the initial parameters based on understanding chemical bonding and its representation via atomic charges, bonded versus nonbonded interactions, LJ, and other parameters consistent with reproducible experimental atomic-scale data (Figure 2f). We also emphasize a strong interpretative feedback loop relative to other validated chemistries (e.g. titania, carbon/organic compounds, alumina etc) that guides refinement and is part of validation.

We do not expect IFF to be robust for all applications, however, we can describe a path to make it more robust if needed.[2, 8] This path involves analysis of failure modes and explaining them in terms of, e.g., (1) did we miss a specific bonding feature (off-center bonds, lone pairs, inherent reactivity, or an unphysical representation),[8, 95] (2) were some LJ terms not representing the true relative atomic sizes and polarizabilities, (3) can we better approximate the electronic structure, e.g., as effectively tried for double/multiple bonds with virtual electrons,[6] (4) improve nonbonded descriptions etc. The inspiration for doing so comes from chemical knowledge of electronic

structure and known reactions, as well as quantum chemical calculations. Hereby, the representation of appropriate features and electronic structure in IFF to solve a shortcoming is hardly mapped 1:1 in an automated way. We consider the workings of the force field (energy expression, exclusion of nearest neighbor nonbond interactions, combination rules), suitable experimental or high-level QM reference data, and interpretation of the parameters relative to the desired chemical and physical effects to guide the most effective representations and refinements.

IFF h demonstrated high accuracy across diverse chemistries (metals, minerals, polymers, electrolytes, gases) due to this defined and scalable approach. At the same time, IFF and IFF-R models can always be further refined depending on the tradeoff between desired predictions versus complexity and ease of use.

S10.15. Availability of Code and Examples. The IFF and IFF-R force field files, 3D models of MXenes with atomic charges and atom types ready for simulations, and well as example files for each type of simulations including run scripts and analysis scripts are shared in the Supporting Files.

Supporting References

1. Wan, S.; Li, X.; Wang, Y.; Chen, Y.; Xie, X.; Yang, R.; Tomsia, A. P.; Jiang, L.; Cheng, Q., Strong Sequentially Bridged MXene Sheets. *Proc. Natl. Acad. Sci. U. S. A.* **2020,** *117*, 27154-27161.
2. Heinz, H.; Lin, T.-J.; Mishra, R. K.; Emami, F. S., Thermodynamically Consistent Force Fields for the Assembly of Inorganic, Organic, and Biological Nanostructures: the INTERFACE Force Field. *Langmuir* **2013,** *29*, 1754-1765.
3. Zhu, C.; Hoff, S. E.; Hémadi, M.; Heinz, H., Accurate and Ultrafast Simulation of Molecular Recognition and Assembly on Metal Surfaces in Four Dimensions. *Acs Nano* **2023,** *17*, 9938-9952.
4. Kanhaiya, K.; Nathanson, M.; in 't Veld, P. J.; Zhu, C.; Nikiforov, I.; Tadmor, E. B.; Choi, Y. K.; Im, W.; Mishra, R. K.; Heinz, H., Accurate Force Fields for Atomistic Simulations of Oxides, Hydroxides, and Organic Hybrid Materials up to the Micrometer Scale. *J. Chem. Theor. Comput.* **2023,** *19*, 8293-8322.
5. Heinz, H.; Vaia, R. A.; Farmer, B. L.; Naik, R. R., Accurate Simulation of Surfaces and Interfaces of Face-Centered Cubic Metals Using 12−6 and 9−6 Lennard-Jones Potentials. *J. Phys. Chem. C* **2008,** *112*, 17281-17290.
6. Pramanik, C.; Gissinger, J. R.; Kumar, S.; Heinz, H., Carbon Nanotube Dispersion in Solvents and Polymer Solutions: Mechanisms, Assembly, and Preferences. *Acs Nano* **2017,** *11*, 12805-12816.
7. Liu, J.; Zeng, J.; Zhu, C.; Miao, J.; Huang, Y.; Heinz, H., Interpretable Molecular Models for Molybdenum Disulfide and Insight into Selective Peptide Recognition. *Chem. Sci.* **2020,** *11*, 8708-8722.
8. Winetrout, J. J.; Kanhaiya, K.; Kemppainen, J.; in 't Veld, P. J.; Sachdeva, G.; Pandey, R.; Damirchi, B.; van Duin, A.; Odegard, G. M.; Heinz, H., Implementing Reactivity in Molecular Dynamics Simulations with Harmonic Force Fields. *Nat. Commun.* **2024,** *15*, 7945.
9. Zhu, H.; Liang, Z.; Xue, S.; Ren, X.; Liang, X.; Xiong, W.; Gao, L.; Liu, A., DFT Practice in MXene-based Materials for Electrocatalysis and Energy Storage: From Basics to Applications. *Ceram. Int.* **2022,** *48* (19, Part A), 27217-27239.
10. Fu, Z. H.; Zhang, Q. F.; Legut, D.; Si, C.; Germann, T. C.; Lookman, T.; Du, S. Y.; Francisco, J. S.; Zhang, R. F., Stabilization and Strengthening Effects of Functional Groups in Two-Dimensional Titanium Carbide. *Phys. Rev. B* **2016,** *94*, 104103.
11. Kazemi, S. A.; Wang, Y., Super Strong 2D Titanium Carbide MXene-Based Materials: a Theoretical Prediction. *J. Phys.: Condens. Matter* **2019,** *32*, 11LT01.
12. Zhan, C.; Sun, W.; Xie, Y.; Jiang, D.-e.; Kent, P. R. C., Computational Discovery and Design of MXenes for Energy Applications: Status, Successes, and Opportunities. *ACS Appl. Mater. Interfaces* **2019,** *11*, 24885-24905.
13. Heinz, H.; Suter, U. W., Atomic Charges for Classical Simulations of Polar Systems. *J. Phys. Chem. B* **2004,** *108*, 18341-18352.
14. Gross, K. C.; Seybold, P. G.; Hadad, C. M., Comparison of Different Atomic Charge Schemes for Predicting pK_a Variations in Substituted Anilines and Phenols. *Int. J. Quantum Chemistry* **2002,** *90*, 445-458.
15. White, B. R.; Wagner, C. R.; Truhlar, D. G.; Amin, E. A., Molecular Modeling of Geometries, Charge Distributions, and Binding Energies of Small, Drug-Like Molecules

Containing Nitrogen Heterocycles and Exocyclic Amino Groups in the Gas Phase and Aqueous Solution. *J. Chem. Theory Comput.* **2008,** *4*, 1718-1732.
16. Wang, B.; Li, S. L.; Truhlar, D. G., Modeling the Partial Atomic Charges in Inorganometallic Molecules and Solids and Charge Redistribution in Lithium-Ion Cathodes. *J. Chem. Theory Comput.* **2014,** *10*, 5640-5650.
17. Plummer, G.; Anasori, B.; Gogotsi, Y.; Tucker, G. J., Nanoindentation of Monolayer Ti C T MXenes via Atomistic Simulations: The Role of Composition and Defects on Strength. *Comput. Mater. Sci.* **2019,** *157*, 168-174.
18. Lipatov, A.; Lu, H.; Alhabeb, M.; Anasori, B.; Gruverman, A.; Gogotsi, Y.; Sinitskii, A., Elastic Properties of 2D Ti3C2Tx MXene Monolayers and Bilayers. *Sci. Adv.* **2018,** *4*, eaat0491.
19. Borysiuk, V. N.; Mochalin, V. N.; Gogotsi, Y., Molecular Dynamic Study of the Mechanical Properties of Two-Dimensional Titanium Carbides Ti_{n+1} C_n (MXenes). *Nanotechnology* **2015,** *26*, 265705.
20. Hatam-Lee, S. M.; Esfandiar, A.; Rajabpour, A., Mechanical Behaviors of Titanium Nitride and Carbide MXenes: A Molecular Dynamics Study. *Appl. Surf. Sci.* **2021,** *566*, 150633.
21. Zartman, G. D.; Liu, H.; Akdim, B.; Pachter, R.; Heinz, H., Nanoscale Tensile, Shear, and Failure Properties of Layered Silicates as a Function of Cation Density and Stress. *J. Phys. Chem. C* **2010,** *114*, 1763-1772.
22. Zhou, T.; Wu, C.; Wang, Y.; Tomsia, A. P.; Li, M.; Saiz, E.; Fang, S.; Baughman, R. H.; Jiang, L.; Cheng, Q., Super-Tough MXene-Functionalized Graphene Sheets. *Nat. Com.* **2020,** *11*, 2077.
23. Meidani, K.; Cao, Z.; Barati Farimani, A., Titanium Carbide MXene for Water Desalination: A Molecular Dynamics Study. *ACS Appl. Nano Mater.* **2021,** *4*, 6145-6151.
24. Ding, L.; Wei, Y.; Li, L.; Zhang, T.; Wang, H.; Xue, J.; Ding, L.-X.; Wang, S.; Caro, J.; Gogotsi, Y., MXene Molecular Sieving Membranes for Highly Efficient Gas Separation. *Nat. Com.* **2018,** *9*, 155.
25. Heinz, H.; Koerner, H.; Anderson, K. L.; Vaia, R. A.; Farmer, B. L., Force Field for Mica-Type Silicates and Dynamics of Octadecylammonium Chains Grafted to Montmorillonite. *Chem. Mater.* **2005,** *17*, 5658-5669.
26. Zhang, D.; Ashton, M.; Ostadhossein, A.; van Duin, A. C. T.; Hennig, R. G.; Sinnott, S. B., Computational Study of Low Interlayer Friction in Tin+1Cn (n = 1, 2, and 3) MXene. *ACS Appl. Mater. Interfaces* **2017,** *9*, 34467-34479.
27. Prokudina, V. K., *Abrasives*. Elsevier, Amsterdam: 2017; pp. 2-5.
28. Lide, D. R., *CRC Handbook of Chemistry and Physics*. 101st Edition ed.; CRC Press: Boca Raton, Florida, 2020.
29. Liu, J.; Tennessen, E.; Miao, J.; Huang, Y.; Rondinelli, J. M.; Heinz, H., Understanding Chemical Bonding in Alloys and the Representation in Atomistic Simulations. *J. Phys. Chem. C* **2018,** *122*, 14996-15009.
30. Goerigk, L.; Grimme, S., A Thorough Benchmark of Density Functional Methods for General Main Group Thermochemistry, Kinetics, and Noncovalent Interactions. *Phys. Chem. Chem. Phys.* **2011,** *13*, 6670-88.
31. Ruiz, V. G.; Liu, W.; Tkatchenko, A., Density-Functional Theory with Screened van der Waals Interactions Applied to Atomic and Molecular Adsorbates on Close-Packed and Non-Close-Packed Surfaces. *Phys. Rev. B* **2016,** *93*, 035118.

32. Dharmawardhana, C. C.; Kanhaiya, K.; Lin, T.-J.; Garley, A.; Knecht, M. R.; Zhou, J.; Miao, J.; Heinz, H., Reliable Computational Design of Biological-Inorganic Materials to the Large Nanometer Scale using Interface-FF. *Molec. Sim.* **2017,** *43*, 1394-1405.
33. Sauer, J., Ab Initio Calculations for Molecule-Surface Interactions with Chemical Accuracy. *Acc Chem Res* **2019,** *52*, 3502-3510.
34. Brooks, B. R.; Bruccoleri, R. E.; Olafson, B. D.; States, D. J.; Swaminathan, S.; Karplus, M., CHARMM: A Program for Macromolecular Energy, Minimization, and Dynamics Calculations. *J. Comp. Chem.* **2004,** *4*, 187-217.
35. Weiner, P. K.; Kollman, P. A., AMBER: Assisted Model Building with Energy Refinement. A General Program for Modeling Molecules and their Interactions. *J. Comp. Chem.* **2004,** *2*, 287-303.
36. Jorgensen, W. L., Development and Testing of the OPLS All-Atom Force Field on Conformational Energetics and Properties of Organic Liquids. *J. Am. Chem.* **1996,** *118*, 11225-11236.
37. Sun, H.; Mumby, S. J.; Maple, J. R.; Hagler, A. T., An ab Initio CFF93 All-Atom Force Field for Polycarbonates. *J. Am. Chem.* **1994,** *116*, 2978-2987.
38. Sun, H., Ab Initio Calculations and Force Field Development for Computer Simulation of Polysilanes. *Macromol.* **1995,** *28*, 701-712.
39. Sun, H., COMPASS: An ab Initio Force-Field Optimized for Condensed-Phase Applications: Overview with Details on Alkane and Benzene Compounds. *J. Phys. Chem* **1998,** *102*, 7338-7364.
40. Asensio, J. L. M. M.-P. J.-B., Jesus, The Use of CVFF and CFF91 Force Fields in Conformational Analysis of Carbohydrate Molecules. Comparison with AMBER Molecular Mechanics and Dynamics Calculations for Methyl-Lactoside. *Int. Journal Bio. Macromol.* **1994,** *17*, 3-4.
41. Mayo, S. L., DREIDING: A Generic Force Field for Molecular Simulations. *J. Phys. Chem.* **1990,** *94*, 8897-8909.
42. Sun, H.; Mumby, S. J.; Maple, J. R.; Hagler, A. T., An Ab-Initio CFF93 All-Atom Force Field for Polycarbonates. *J. Am. Chem. Soc.* **1994,** *116*, 2978-2987.
43. Yang, J.; Ren, Y.; Tian, A. M.; Sun, H. A., COMPASS Force Field for 14 Inorganic Molecules, He, Ne, Ar, Kr, Xe, H-2, O-2, N-2, NO, CO, CO2, NO2, CS2, and SO2, in Liquid Phases. *J. Phys. Chem. B* **2000,** *104*, 4951-4957.
44. Sun, H., COMPASS: An Ab Initio Force-Field Optimized for Condensed-Phase Applications - Overview with Details on Alkane and Benzene Compounds. *J. Phys. Chem. B* **1998,** *102*, 7338-7364.
45. Mishra, R. K.; Fernández-Carrasco, L.; Flatt, R. J.; Heinz, H., A Force Field for Tricalcium Aluminate to Characterize Surface Properties, Initial Hydration, and Organically Modified Interfaces in Atomic Resolution. *Dalton Trans.* **2014,** *43*, 10602-10616.
46. Emami, F. S.; Puddu, V.; Berry, R. J.; Varshney, V.; Patwardhan, S. V.; Perry, C. C.; Heinz, H., Force Field and a Surface Model Database for Silica to Simulate Interfacial Properties in Atomic Resolution. *Chem. Mater.* **2014,** *26*, 2647-2658.
47. Wang, S.; Hou, K.; Heinz, H., Accurate Compatible Force Fields for Molecular Oxygen, Nitrogen and Hydrogen to Simulate Heterogeneous Electrolytes and Interfaces. *J. Chem. Theor. Comput.* **2021,** *17*, 5198-5213.

48. Mishra, R. K.; Kanhaiya, K.; Winetrout, J. J.; Flatt, R. J.; Heinz, H., Force Field for Calcium Sulfate Minerals to Predict Structural, Hydration, and Interfacial Properties. *Cem. Concr. Res.* **2021,** *139*, 106262.
49. Sarycheva, A.; Gogotsi, Y., Raman Spectroscopy Analysis of the Structure and Surface Chemistry of Ti3C2Tx MXene. *Chem. of Mater.* **2020,** *32*, 3480-3488.
50. Rasel, M. A. J.; Wyatt, B.; Wetherington, M.; Anasori, B.; Haque, A., Low-Temperature Annealing of 2D Ti3C2Tx MXene Films using Electron Wind Force in Ambient Conditions. *J. Mater. Res.* **2021,** *36*, 3398-3406.
51. Rafiq, S.; Awan, S.; Zheng, R.-K.; Wen, Z.; Rani, M.; Akinwande, D.; Rizwan, S., Novel Room-Temperature Ferromagnetism in Gd-doped 2-Dimensional Ti3C2Tx MXene Semiconductor for Spintronics. *J. Magn. Magn. Mater.* **2020,** *497*, 165954.
52. Limbu, T. B.; Chitara, B.; Garcia Cervantes, M. Y.; Zhou, Y.; Huang, S.; Tang, Y.; Yan, F., Unravelling the Thickness Dependence and Mechanism of Surface-Enhanced Raman Scattering on Ti3C2TX MXene Nanosheets. *J.Phys. Chem. C* **2020,** *124*, 17772-17782.
53. Yorulmaz, U.; Özden, A.; Perkgöz, N. K.; Ay, F.; Sevik, C., Vibrational and Mechanical Properties of Single Layer MXene Structures: a First-Principles Investigation. *Nanotechnology* **2016,** *27*, 335702.
54. Sarycheva, A.; Shanmugasundaram, M.; Krayev, A.; Gogotsi, Y., Tip-Enhanced Raman Scattering Imaging of Single- to Few-Layer Ti3C2Tx MXene. *ACS Nano* **2022,** *16*, 6858-6865.
55. Li, Y.; Wei, C.; Huang, S.; Wu, C.; Mochalin, V. N., In-situ SEM Compression of Accordion-like Multilayer MXenes. *Extr. Mech. Lett.* **2020,** *41*, 101054.
56. Zhou, H., Study on Contact Angles and Surface Energy of MXene Films. *RSC Adv.* **2021,** *11*, 5512.
57. Hautman, J.; Klein, M. L., Microscopic Wetting Phenomena. *Phys. Rev. Lett.* **1991,** *67*, 1763-1766.
58. Thomsen, F. *Practical Contact Angle Measurement*; KRUSS: Kruss Technical Newsletters, December 2008, 2008.
59. Emami, F. S.; Puddu, V.; Berry, R. J.; Varshney, V.; Patwardhan, S. V.; Perry, C. C.; Heinz, H., Prediction of Specific Biomolecule Adsorption on Silica Surfaces as a Function of pH and Particle Size. *Chem. Mater.* **2014,** *26*, 5725–5734.
60. Li, Y.; Wei, C.; Huang, S.; Ghasemi, A.; Gao, W.; Wu, C.; Mochalin, V. N., In Situ Tensile Testing of Nanometer-Thick Two-Dimensional Transition-Metal Carbide Films: Implications for MXenes Acting as Nanoscale Reinforcement Agents. *ACS Appl. Nano Mater.* **2021,** *4*, 5058-5067.
61. Firestein, K. L.; von Treifeldt, J. E.; Kvashnin, D. G.; Fernando, J. F. S.; Zhang, C.; Kvashnin, A. G.; Podryabinkin, E. V.; Shapeev, A. V.; Siriwardena, D. P.; Sorokin, P. B.; Golberg, D., Young's Modulus and Tensile Strength of Ti3C2 MXene Nanosheets As Revealed by In Situ TEM Probing, AFM Nanomechanical Mapping, and Theoretical Calculations. *Nano Lett.* **2020,** *20*, 5900-5908.
62. Guo, Z.; Zhou, J.; Sun, Z., Flexible Two-Dimensional Tin+1 Cn (n = 1, 2 and 3) and Their Functionalized MXenes Predicted by Density Functional Theories. *Phys. Chem. Chem. Phys.* **2015,** *17*, 15348.

63. Li, J.; Niu, J.; Kim, Y.; Chae, H., Surface Wettability Control and Fluorination Modeling of Amorphous Carbon Films Fluorinated with CF4 Plasma. *Appl. Surf. Sci.* **2023,** *635*, 157668.
64. Geada, I. L.; Ramezani-Dakhel, H.; Jamil, T.; Sulpizi, M.; Heinz, H., Insight into Induced Charges at Metal Surfaces and Biointerfaces Using a Polarizable Lennard-Jones Potential. *Nat. Comm.* **2018,** *9*, 716.
65. Mishra, R. K.; Darouich, S.; in 't Veld, P. J.; Flatt, R. J.; Heinz, H., Understanding Hydration Reactions, Mechanical Properties, Thermal Expansion, and Organic Interfacial Interactions of Calcium Sulfate Hydrates from the Atomic Scale. *Cem. Concr. Res.* **2025,** *189*, 107740.
66. Winetrout, J. J.; Li, Z.; Zhao, Q.; Gaber, L.; Unnikrishnan, V. U.; Varshney, V.; Xu, Y.; Wang, Y.; Heinz, H., Prediction of Carbon Nanostructure Mechanical Properties and the Role of Defects Using Machine Learning. *Proc. Natl. Acad. Sci. U. S. A.* **2025,** *122*, e2415068122.
67. Bamane, S. S.; Jakubinek, M. B.; Kanhaiya, K.; Ashrafi, B.; Heinz, H.; Odegard, G. M., Boron Nitride Nanotubes: Force Field Parameterization, Epoxy Interactions, and Comparison with Carbon Nanotubes for High-Performance Composite Materials. *ACS Appl. Nano Mater.* **2023,** *6*, 3513-3524.
68. Odegard, G. M.; Patil, S. U.; Deshpande, P. P.; Kanhaiya, K.; Winetrout, J. J.; Heinz, H.; Shah, S. P.; Maiaru, M., Molecular Dynamics Modeling of Epoxy Resins Using the Reactive Interface Force Field. *Macromolecules* **2021,** *54*, 9815-9824.
69. Damirchi, B.; Radue, M.; Kanhaiya, K.; Heinz, H.; Odegard, G. M.; van Duin, A. C. T., ReaxFF Reactive Force Field Study of Polymerization of a Polymer Matrix in a Carbon Nanotube-Composite System. *J. Phys. Chem. C* **2020,** *124*, 20488-20497.
70. Bryenton, K. R.; Adeleke, A. A.; Dale, S. G.; Johnson, E. R., Delocalization Error: The Greatest Outstanding Challenge in Density-Functional Theory. *WIREs Computational Molecular Science* **2023,** *13*, e1631.
71. Gholivand, H.; Fuladi, S.; Hemmat, Z.; Salehi-Khojin, A.; Khalili-Araghi, F., Effect of Surface Termination on the Lattice Thermal Conductivity of Monolayer Ti3C2Tz MXenes. *J. Appl. Phys.* **2019,** *126*, 065101.
72. Sang, X.; Xie, Y.; Lin, M.-W.; Alhabeb, M.; Van Aken, K. L.; Gogotsi, Y.; Kent, P. R. C.; Xiao, K.; Unocic, R. R., Atomic Defects in Monolayer Titanium Carbide (Ti3C2Tx) MXene. *Acs Nano* **2016,** *10*, 9193-9200.
73. Shi, C.; Beidaghi, M.; Naguib, M.; Mashtalir, O.; Gogotsi, Y.; Billinge, S. J. L., Structure of Nanocrystalline Ti 3 C 2 MXene Using Atomic Pair Distribution Function. *Phys. Rev. Lett.* **2014,** *112*, 125501.
74. *Interface Force Field (IFF) and a Surface Model Database*. https://bionanostructures.com/interface-md/. 2013-2026.
75. Bussi, G.; Donadio, D.; Parrinello, M., Canonical Sampling Through Velocity Rescaling. *J. Chem. Phys.* **2007,** *126*, 014101.
76. Parrinello, M.; Rahman, A., Crystal Structure and Pair Potentials: A Molecular-Dynamics Study. *Phys. Rev. Lett.* **1980,** *45*, 1196-1199.
77. Kleinhesselink, D.; Wolfsberg, M., The Evaluation of Power Spectra in Molecular Dynamics Simulations of Anharmonic Solids and Surfaces. *Surf. Sci.* **1992,** *262*, 189-207.

78. WebPlotDigitizer - Extract data from plots, images, and maps. https://automeris.io/WebPlotDigitizer/citation.html.
79. Heinz, H.; Vaia, R. A.; Farmer, B. L., Interaction Energy and Surface Reconstruction Between Sheets of Layered Silicates. *J. Chem. Phys.* **2006,** *124*, 224713.
80. Zukiene, K.; Monastyreckis, G.; Kilikevicius, S.; Procházka, M.; Micusik, M.; Omastová, M.; Aniskevich, A.; Zeleniakiene, D., Wettability of MXene and its Interfacial Adhesion with Epoxy Resin. *Mater. Chem. Phys.* **2021,** *257*, 123820.
81. Zhou, H.; Wang, F.; Wang, Y.; Li, C.; Shi, C.; Liua, Y.; Ling, Z., Study on Contact Angles and Surface Energy of MXene Films. *RSC Adv.* **2020,** *11*, 5512-5520.
82. Bian, R.; Xiang, S.; Cai, D., Fast Treatment of MXene Films with Isocyanate to Give Enhanced Stability. *Chem. Nano. Mat.* **2020,** *6*, 64-67.
83. Lorencova, L.; Bertok, T.; Desekova, E.; Holazova, A.; Paprckova, D.; Vikartovska, A.; Sasinkova, V.; Filip, J.; Kasak, P.; Jerigova, M.; Velic, D.; Mahmoud, K. A.; Tkac, J., Electrochemical Performance of $Ti_3C_2T_x$ MXene in Aqueous Media: Towards Ultrasensitive H_2O_2 Sensing. *Electrochim. Acta* **2017,** *235*, 471-479.
84. Talouba, N.; Hennicheb, A.; Liua, L.; Lia, J.; Rahouia, N.; Hegazya, M.; Huanga, Y., Improving the Mechanical Properties, UV and Hydrothermal Aging Resistance of PIPD Fiber using MXene (Ti3C2(OH)2) Nanosheets. *Composites Part B* **2019,** *163*, 260-271.
85. Jorgensen, W. L.; Chandrasekhar, J.; Madura, J. D.; Impey, R. W.; Klein, M. L., Comparison of simple potential functions for simulating liquid water. *J. Chem. Phys.* **1983,** *79*, 926-935.
86. Kim, S. J.; Choi, J.; Maleski, K.; Hantanasirisakul, K.; Jung, H.-T.; Gogotsi, Y.; Ahn, C. W., Interfacial Assembly of Ultrathin, Functional MXene Films. *ACS Appl. Mater. Interfaces* **2019,** *11*, 32320-32327.
87. Phillips, J. C.; Braun, R.; Wang, W.; Gumbart, J.; Tajkhorshid, E.; Villa, E.; Chipot, C.; Skeel, R. D.; Kale, L.; Schulten, K., Scalable Molecular Dynamics with NAMD. *J. Comput. Chem.* **2005,** *26*, 1781-1802.
88. Heinz, H., Computational Screening of Biomolecular Adsorption and Self-Assembly on Nanoscale Surfaces. *J. Comput. Chem.* **2010,** *31*, 1564-1568.
89. Humphrey, W.; Dalke, A.; Schulten, K., VMD - Visual Molecular Dynamics. *J. Mol. Graphics* **1996,** *14*, 33-38.
90. Zartman, G.; Liu, H.; Akdim, B.; Pachter, R.; Heinz, H., Nanoscale Tensile, Shear, and Failure Properties of Layered Silicates as a Function of Cation Density and Stress. *J. Phys. Chem. C* **2010,** *114*, 1763-1772.
91. Hadizadeh Kheirkhah, A.; Saeivar Iranizad, E.; Raeisi, M.; Rajabpour, A., Mechanical Properties of Hydrogen Functionalized Graphene under Shear Deformation: A Molecular Dynamics Study. *Solid State Commun.* **2014,** *177*, 98-102.
92. Min, K.; Aluru, N. R., Mechanical Properties of Graphene under Shear Deformation. *Appl. Phys. Lett.* **2011,** *98*, 013113.
93. Varshney, V.; Patnaik, S. S.; Muratore, C.; Roy, A. K.; Voevodin, A. A.; Farmer, B. L., MD Simulations of Molybdenum Disulphide (MoS2): Force-Field Parameterization and Thermal Transport Behavior. *Comp. Mater. Sci.* **2010,** *48*, 101-108.
94. Zhang, X.; Nguyen, H.; Paci, J. T.; Sankaranarayanan, S. K. R. S.; Mendoza-Cortes, J. L.; Espinosa, H. D., Multi-objective Parametrization of Interatomic Potentials for Large Deformation Pathways and Fracture of Two-dimensional Materials. *npj Computational Materials* **2021,** *7*, 113.

95. Kanhaiya, K.; Kim, S.; Im, W.; Heinz, H., Accurate Simulation of Surfaces and Interfaces of Ten FCC Metals and Steel Using Lennard–Jones Potentials. *npj Comput. Mater.* **2021,** *7*, 17.
