# Supplementary material for: Validated Reactive Force Field Quantifies MXene Interfacial Properties, Mechanics, and Thermal Transport
Source: ACS Nano. 2026 Jul 22;20(30):20979–97. doi: 10.1021/acsnano.5c20046 (PMC13450432; doi:10.1021/acsnano.5c20046)
Supplement: Supplementary file 2 [file nn5c20046_si_002.zip › SUPPORTING FILES/README Supporting Files.docx]

The Supporting Files are organized into the following folders and subfolders as follows:

**Force Field Files:** This folder contains the force field files (.frc file and templates.dat file) for MXenes that includes all IFF parameters in CVFF format and PCFF format (Version 1.6). The templates.dat file enables use within Materials Studio (using the provided atom-typed and charged models structures, no automated typing of MXenes). The folder also contains the IFF force field for MXenes in CHARMM36 format (.prm file). This file is integrated with CHARMM36 to simulate biomolecular/MXene systems as shown in simulations with dopamine. Protonated surface groups Ti(OH2)(+) Cl(-) at pH <6 and deprotonated surface groups TiO(-) Na(+) at pH > 6 can be included in all force fields.

For use with IFF-R, we suggest using LAMMPS. Harmonic bond coefficients for Ti-C bonds are overwritten and replaced using the Morse bond parameters, as shown in the input (.inp or .lmp) scripts for nanoindentation (see LAMMPS INPUTS folder).

We also include an earlier force field version 1.5 in the OLD sub-folder that was used for initial calculations (slightly lower accuracy for surface parameters).

Use in common harmonic force fields: IFF parameters are ready for use with CVFF, OPLS-AA, CHARMM, AMBER, PCFF, CFF, COMPASS and others (e.g., DREIDING). Parameters for CHARMM and AMBER use a smaller r_min_ for Ti1 (int) (2.25 --> 2.16 Å) and Ti2 (ext) (2.15 --> 2.06 Å) due to arithmetic combination rules rather than geometric combination rules. No other changes are needed. Simulations in PCFF, CFF, and COMPASS formats should use the given 9-6 LJ parameters. Reactive simulations using IFF-R with shifted Morse bonds are enabled in all these force fields.

Flexibility for nonbonded versus bonded surface groups: By default, we use nonbonded interactions for the surface groups (OH and F) with the Ti carbide framework. If a bonded description is desired for these and similar covalent modifications, the optional bonded terms below (bonds and angles) for OH and F surface groups can be used and further customized:

Bonds

4.0 33	ti2	fx	2.1060	200.000

4.0 33	ti2	omx	2.1060	200.000

Angles

4.0 33	cx1/cx2/cx3/cx4	ti2	fx	90.0000	80.0000

4.0 33	cx1/cx2/cx3/cx4	ti2	omx	90.0000	80.0000

4.0 33	ti2	omx	hoy	115.0000	45.0000

4.0 33	ti2	omx	ti2	92.8000	80.0000

4.0 33	ti2	fx	ti2	92.8000	80.0000

**Unit Cell, Surface, and Defect Models:** This folder contains 3D periodic all-atom models of the Ti3C2(OH, F)2 structures, including unit cells (hexagonal), equivalent rectangular versions of the unit cells (composed of two hexagonal unit cells), and 3 models with common defects. The unit cells are given for different surface termination. The subfolder with surface models contains large supercells with more detailed variation in surface composition (-OH versus -F). Surface models with protonation at pH 5 are also included.

All models are provided in car/mdf format, directly readable in Materials Studio, easy to modify, and ready for simulations. Files are also included as LAMMPS data files (including force field information) and in pdb/psf format for aqueous interfaces and MXene/biomolecules simulations, ready for simulations with LAMMPS or NAMD.

Our reported calculations utilized the models in these folders, replicated and stacked into different geometries. For modification and adaptation to other programs, please (1) keep the atomic charges and (2) keep the atom types and parameters as is. Do not use different charge schemes or atom typing schemes, which would void the accuracy of the IFF models.

*Ti3C2Tx Unit Cells and Rectangular Cells:* Hexagonal unit cells are provided for 100% –F, 100% –OH, and 50/50 splits of –F and –OH, as well as the corresponding rectangular super cells. We used the X-ray data in

Shi et al., Phys. Rev. Lett. 2014, 112, 125501, DOI: 10.1103/PhysRevLett.112.125501.

Orthogonal cells are often more convenient to use and contain identical atomic positions. All models include atomic charges and atom types, ready for MM/MD simulations.

*MXene Surface Models OH, F:* Surface models with variable percentage of -OH versus -F termination are provided as hexagonal supercells: 0%, 20%, 40%, 47%, 60%, 80%, and 100% –OH. The difference to 100% is -F termination. 47% -OH is included as it matches experimental data.

*MXene Surface-Liquid Models OH, F:* For each of these surface models, models of the interfaces with water and diiodomethane are given, which were further processed for contact angle simulations.

*Defects Models:* Three defect models as described in Section S9 in the Supporting Information.

**Biomolecular Binding, pH, and CHARMM Integration:** This folder contains the models for dopamine chloride assembly on the Ti_3_C_2_T_x_ MXene surfaces in solution, as well as for the components the system. These include pH-resolved surface models (pH 5), single dopamine chloride molecules, and dopamine aggregates. The combined models are given with CHARMM36 parameters in pdb/psf format as used in simulations with NAMD, and initial models with CHARMM36 parameters also car/mdf format. On the MXene surfaces with 47% -OH and 53% -F groups, 3% of the OH groups are protonated at pH 5. See README file in in this folder for further information.

**Run Scripts and Examples in LAMMPS and NAMD:** This folder contains run scripts and example data for the reported calculations. Structure/data files are typically excluded due to space limitations. The representative workflow involved model creation in Materials Studio, MD simulations in LAMMPS (or NAMD for biomolecular systems), and analysis of LAMMPS outputs using Matlab scripts.

*LAMMPS cleavage energies*: One example is provided for a 2-layer cleavage energy calculation. The LAMMPS inputs subfolder includes two input scripts for 2-layer surfaces with 50/50 splits of –F and –OH termination and the corresponding bulk system. The scripts can be copied for different surface terminations. MATLAB analysis subfolder contains data and tested analysis scripts to calculate the cleavage energy for 6 systems: for 100% –F, 100% –OH, and 50/50 splits of –F and –OH of 1-layer and two-layer surfaces.

*LAMMPS contact angle simulations:* One example is provided for 20%–OH/80% -F terminated surfaces in water (labeled SETFORCE) and diiodomethane (labeled DIM), with input scripts and combined liquid droplet-solid systems in LAMMPS .data format (small droplet for demonstration only). The input scripts generate density profiles (PROFILE3) that can be read using the provided MATLAB analysis scripting for two of the examples: 47% OH for water and DIM. The workflow and scripts can be used for other surface terminations.

*MXene Liquid Interfaces:* An example is provided for MXene with 47% –OH termination of the surfaces in water with input scripts and liquid-saturated systems in LAMMPS .data format. The input scripts generate density profiles that can be read using the provided MATLAB analysis scripts to generate graphs (Stack3x3_H2Osys_OH_47.m). Further models of liquid interfaces with water and diiodomethane can be found in the folder “Unit Cell, Surface, and Defect Models/MXene Surface-Liquid Models OH, F “.

*Elastic Constants:* Four examples are provided, linearly deforming 100% –F terminated surfaces in vacuum in tension and compression, with input scripts and systems in LAMMPS .data format. The input scripts generate temp1 files that can be read using the provided MATLAB analysis scripts that generate the stress-strain curves.

*Equilibrium Cell and Density*: Two examples are provided of 2 runs of orthogonal and triclinic 50/50 surfaces lattices in vacuum in LAMMPS .data format, which are run to equilibrium and densities and lattice parameters checked in supplied MATLAB scripts.

*MXene-Dopamine Simulations*: 2 examples of input scripts are provided, for one system without and one system with constraint scaling. The cell size stayed the same in both systems, so only input and output names must be changed. Python scripts used to track dopamine height and orientation over time from dcd files of single and multiple dopamine systems are provided.

*Nanoindentation*: Two example scripts for nanoindentation simulations are provided for non-reactive IFF simulations (used to obtain stress/strain curves) and for reactive IFF-R simulations (used to determine fracture depths and mechanisms). MATLAB analysis scripts are provided for the IFF simulations that plot elastic curves. Data extrapolated from experimental AFM references provided in the main text is provided here and read in by MATLAB script. Note: The IFFR input script overwrites bonds provided in the .data files to include reactive Morse bonds.

*Nanoscale Thermal Conductivity:* One example is provided for 100% -F surface termination using the Fourier law to achieve equilibrated thermal gradients to calculate the thermal conductivity both in-plane and out-of-plane. Data files and output files are included along with Matlab analysis scripts that process the outputs and calculate slopes and thermal conductivities.

*Vibration Spectra Calculations*: Four examples are provided to carry out 512 replicates of lattice dynamics to obtain power spectra for averaging into IR/Raman spectra. The LAMMPS .data file is provided. Once replicate data has been generated, it is collated by a MATLAB script RAMAN_FINAL_K1_A43_WA1.m, which generates the excel table EXPORT_RAMAN_FINAL_K1_A43_WA1.csv. The table headings can be manually modified (stripped of header columns) to make EXPORT_RAMAN_FINAL_K1_A43_WA1_modified.csv. Finally, this modified csv file is read in by a python script that uses a FFT algorithm to plot the resolved power spectra.

*Shear:* Two examples are provided for the static shear deformation workflow (used to obtain shear modulus) and the dynamic shear slip workflow (used to obtain shear strength). For the static shear deformation workflow, degrees of deformation are manually specified in the given script, as shown in the examples of for 1.25 and 1.5 Angstrom shear for 100% –F termination. Each deform condition is read with its own version of the MATLAB analysis script. In the case of dynamic shear slip, many velocity profiles are generated, shown for one replicate in the output folder. These velocities were compiled and plotted using the provided MATLAB script.

*Surface Seeding Scripts*: MATLAB scripting is provided to help seed surfaces to a desired concentration of OH groups. Currently, the script is setup to convert –F groups to –OH groups, but this could be reversed if a new template molecule was provided. The template molecule, “hydroxyl.car/.mdf” is read in by the script and applied to an input file (also in .car/.mdf format) randomly, until the desired –OH% is produced on the surface.
